# HIV drug resistance during antiretroviral therapy scale-up in Uganda, 2012–19: a population-based, longitudinal study

**DOI:** 10.1016/j.lanmic.2025.101218

**Published:** 2025-12

**Authors:** Michael A Martin, Steven James Reynolds, Brian T Foley, Fred Nalugoda, Thomas C Quinn, Steven A Kemp, Margaret Nakalanzi, Edward Nelson Kankaka, Godfrey Kigozi, Robert Ssekubugu, Ravindra K Gupta, Lucie Abeler-Dörner, Joseph Kagaayi, Oliver Ratmann, Christophe Fraser, Ronald Moses Galiwango, David Bonsall, M Kate Grabowski, Rakai Health Sciences Program Members, Larry W. Chang, Ronald M. Galiwango, M. Kate Grabowski, Ronald H. Gray, Jade C. Jackson, Joseph Kagaayi, Edward Nelson Kankaka, Godfrey Kigozi, Oliver Laeyendecker, Thomas C. Quinn, Steven J. Reynolds, John Santelli, David Serwadda, Nelson K. Sewankambo, Joseph Ssekasanvu, Robert Ssekubugu, Victor Ssempijja, Maria J. Wawer, Dorean Nabukalu, Anthony Ndyanabo, Hadijja Nakawooya, Jessica Nakukumba, Grace N. Kigozi, Betty S. Nantume, Nampijja Resty, Jedidah Kambasu, Margaret Nalugemwa, Regina Nakabuye, Lawrence Ssebanobe, Justine Nankinga, Adrian Kayiira, Gorreth Nanfuka, Ruth Ahimbisibwe, Stephen Tomusange, Sarah Kalibbali, Margaret Nakalanzi, Joseph Ouma Otobi, Denis Ankunda, Joseph Lister Ssembatya, John Baptist Ssemanda, Robert Kairania, Emmanuel Kato, Alice Kisakye, James Batte, James Ludigo, Abisagi Nampijja, Steven Watya, Kighoma Nehemia, Margaret Anyokot, Joshua Mwinike, George Kibumba, Paschal Ssebowa, George Mondo, Francis Wasswa, Agnes Nantongo, Rebecca Kakembo, Josephine Galiwango, Geoffrey Ssemango, Andrew D. Redd, Caitlin E. Kennedy, Jennifer Wagman, Philip Kreniske, PANGEA-HIV Members, Lucie Abeler-Dörner, Helen Ayles, David Bonsall, Rory Bowden, Vincent Calvez, Myron Cohen, Ann Dennis, Tulio de Oliveira, Max Essex, Sarah Fidler, Dan Frampton, Christophe Fraser, M. Kate Grabowski, Tanya Golubchik, Ravindra Gupta, Richard Hayes, Joshua Herbeck, Anne Hoppe, Joseph Kagaayi, Pontiano Kaleebu, Paul Kellam, Cissy Kityo, Andrew Leigh Brown, Jairam Lingappa, Sikhulile Moyo, Vladimir Novitsky, Thumbi Ndung’u, Nick Paton, Deenan Pillay, Thomas Quinn, Andrew Rambaut, Oliver Ratmann, Janet Seeley, Deogratius Ssemwanga, Frank Tanser, Maria Wawer

**Affiliations:** a Department of Pathology, Johns Hopkins School of Medicine, Baltimore, MD, USA; b Department of Epidemiology, Johns Hopkins Bloomberg School of Public Health, Baltimore, MD, USA; c Rakai Health Sciences Program, Kalisizo, Uganda; d Division of Infectious Disease, Department of Medicine, Johns Hopkins School of Medicine, Baltimore, MD, USA; e Division of Intramural Research, National Institute of Allergy and Infectious Diseases, National Institutes of Health, Bethesda, MD, USA; f Theoretical Biology and Biophysics, Los Alamos National Laboratory, Los Alamos, NM, USA; g Department of Medicine, University of Cambridge, Cambridge, UK; h Africa Health Research Institute, KwaZulu-Natal, South Africa; i Pandemic Sciences Institute, Nuffield Department of Medicine, University of Oxford, Oxford, UK; j Makerere University School of Public Health, Kampala, Uganda; k Department of Mathematics, Imperial College London, London, UK; l Centre for Human Genetics, Nuffield Department of Medicine, University of Oxford, Oxford, UK

## Abstract

**Background:**

With scale-up of antiretroviral therapy (ART) in sub-Saharan Africa, increasing pretreatment HIV drug resistance has been reported; however, the broader effect of ART expansion on population-level resistance patterns remains insufficiently quantified. We aimed to estimate the longitudinal prevalence of drug resistance and resistance-conferring mutations.

**Methods:**

This study used data collected as part of the Rakai Community Cohort Study (RCCS), an open population-based census and cohort study conducted in southern Uganda. At each survey round, residents aged 15–49 years are invited to participate and receive a structured questionnaire that obtains sociodemographic, behavioural, and health information, including self-reported past and current ART use. Voluntary HIV testing is conducted using a rapid test algorithm and a venous blood sample. People with HIV provide samples for viral load quantification and deep sequencing. We analysed RCCS survey, HIV viral load, and deep sequencing (which was used to predict resistance) data from five survey rounds. The key outcomes were the population prevalence of viraemic people with HIV with non-nucleoside reverse transcriptase inhibitor (NNRTI), nucleoside reverse transcriptase inhibitor (NRTI), protease inhibitor, or multiclass resistance among all participants (regardless of HIV serostatus) in the 2015 and 2017 surveys. Prevalence of class-specific resistance and resistance-conferring substitutions were estimated using robust log-Poisson regression.

**Findings:**

Between Aug 10, 2011, and Nov 4, 2020, there were 43 361 participants in the RCCS and 7923 (18·27%) people with HIV. Over five survey rounds, 93 622 participant visits occurred, among which 17 460 (18·65%) were from people with HIV. Over the analysis period, the median age of study participants remained similar (28 years [22–35] in 2012 and 29 years [21–38] in 2019). Sufficient data were available to reliably genotype 4072 (90·03%) of 4523 participant visits from 3407 people with HIV for at least one drug. Overall population prevalence of resistance contributed by viraemic pretreatment people with HIV decreased between 2012 and 2017 from 0·56% (95% CI 0·42–0·75) to 0·25% (0·18–0·33) for NNRTI and from 0·24% (0·15–0·37) to 0·05% (0·02–0·10) for NRTI (prevalence ratio 0·44 [0·29–0·68] for NNRTI and 0·21 [0·09–0·47] for NRTI). Between 2012 and 2017, NNRTI resistance among viraemic pretreatment people with HIV increased from 4·86% (3·69–6·42) to 9·61% (7·27–12·7; prevalence ratio 1·98 [1·34–2·91]). The prevalence of NNRTI and NRTI resistance was substantially higher among viraemic treatment-experienced people with HIV (51·49% [46·24–57·34] for NNRTI and 36·46% [30·06–44·22] for NRTI in 2017) than among pretreatment people with HIV. NNRTI and NRTI resistance was predominantly attributable to rtK103N and rtM184V. inT97A was observed at a similar prevalence among viraemic treatment-experienced (9·96% [6·41–15·48]) and viraemic pretreatment (10·56% [8·01–13·93]) people with HIV; no major dolutegravir resistance mutations were observed.

**Interpretation:**

Despite rising NNRTI resistance among pretreatment people with HIV, overall population prevalence of pretreatment HIV drug-resistant viraemia decreased due to increasing ART uptake and viral suppression. This finding underscores the crucial role of achieving and maintaining high ART coverage in reducing transmission of drug-resistant HIV. The high prevalence of mutations conferring resistance to components of first-line ART regimens among viraemic people with HIV is potentially concerning.

**Funding:**

National Institutes of Health, Johns Hopkins University Center for AIDS Research, Bill & Melinda Gates Foundation, and the US Centers for Disease Control and Prevention.

## Introduction

Antiretroviral therapy (ART) suppresses HIV replication in people with HIV,^1^ which slows disease progression^2^ and prevents viral transmission.^3^ With increased ART coverage and scale-up of other interventions, HIV incidence has fallen by approximately 40% globally since 2010.^4^

**Research in context**

**Evidence before this study**

We searched PubMed for studies published in English between Jan 1, 2004 (the beginning of antiretroviral therapy [ART] availability in sub-Saharan Africa), and Aug 31, 2024, using the search terms “HIV”, “resistance”, “longitudinal”, “cohort”, and “population”. We identified 50 studies and excluded 34 that were not based in sub-Saharan Africa. Five studies primarily focused on infection with other pathogens (eg, hepatitis B virus and *Mycobacterium tuberculosis*), two on insulin resistance, one on sequencing methods, one on host susceptibility to HIV infection, and seven only included people living with HIV enrolled at clinics. In light of rapid treatment scale-up and falling HIV incidence, the implications of increased resistance among pretreatment people with HIV on population prevalence of resistance among all people (regardless of HIV serostatus) cannot be quantified without longitudinal data.

**Added value of this study**

Despite an increase in the prevalence of non-nucleoside reverse transcriptase inhibitor (NNRTI) resistance among viraemic pretreatment people with HIV between 2012 and 2017 in Uganda (prevalence ratio 1·98 [95% CI 1·34–2·91]), the overall prevalence of viraemic pretreatment resistance in the entire population decreased (prevalence ratio 0·44 [0·29–0·68]) alongside increasing ART uptake and viral suppression among people with HIV. Most resistance in the later surveys was contributed by treatment-experienced people with HIV. We observed a substantial burden of resistance mutations in viraemic treatment-experienced people with HIV to the NNRTI (eg, rtK103N [25·68%], rtY181C [14·67%], and rtG190A [12·34%]) and NRTI (eg, rtM184V [33·89%] and rtK65R [12·07%]) components of dolutegravir-based and cabotegravir-based regimens. The integrase strand transfer inhibitor (INSTI) resistance mutation inT97A had a similar prevalence among viraemic treatment-experienced (9·96% [6·41–15·48]) and viraemic pretreatment (10·56% [8·01–13·93]) people with HIV. To our knowledge, these are the first longitudinal population-based estimates of temporal trends in the prevalence of drug resistance during ART expansion in a high-burden setting.

**Implications of all the available evidence**

ART scale-up has reduced the burden of pretreatment HIV drug-resistant viraemia by improving viral load suppression at the population level; however, the expansion of HIV treatment in sub-Saharan Africa has increased the prevalence of drug resistance mutations among viraemic pretreatment people with HIV. The high prevalence of NNRTI resistance in this group has prompted a shift to first-line regimens including dolutegravir (an INSTI) in combination with NRTIs. Given the high prevalence of mutations conferring resistance to components of current first-line regimens, ongoing surveillance of unsuccessful treatment and drug resistance remains crucial in high-burden settings, particularly if ART coverage declines.

Viral resistance to ART threatens the clinical and public health impact of treatment scale-up.^5^^,^^6^ Resistance can be acquired from a susceptible virus by within-individual evolution after treatment initiation, which is more common when treatment adherence is intermittent,^7^ but can occur despite high adherence.^8^ In sub-Saharan Africa, the epicentre of the HIV epidemic,^4^ most patients who remain viraemic despite receiving treatment harbour resistance to at least one component of first-line regimens.^1^ Viruses harbouring resistance mutations can be transmitted to seronegative individuals, leading to a five-times increase in unsuccessful treatment compared to individuals with HIV who have no resistance mutations.^9^

Until 2018, first-line ART regimens were a combination of nucleoside reverse transcriptase inhibitors (NRTIs) and non-NRTIs (NNRTIs).^10^ During ART scale-up, the prevalence of NNRTI resistance among pretreatment people with HIV initiating care increased globally, reaching 10% by 2016 in east Africa.^11^ These findings have been corroborated by cross-sectional studies and WHO surveys,^1^^,^^12^ and prompted a shift to dolutegravir (an integrase strand-transfer inhibitor [INSTI]) in combination with NRTIs (eg, tenofovir disoproxil fumarate and lamivudine) for first-line ART. Additionally, long-lasting injectable INSTIs (eg, cabotegravir with the NNRTI rilpivirine) are being rolled out in sub-Saharan Africa.^13^

Most data on the prevalence of HIV drug resistance in sub-Saharan Africa are derived from people with HIV who seek care in clinics or hospitals.^1^^,^^11^^,^^12^ As a result, clinic-based studies do not capture those with HIV who have never been diagnosed or have been lost to care, reducing their ability to quantify changes in population-level prevalence of drug-resistant viraemia. Moreover, accurately estimating the burden of drug-resistant HIV requires data from people with and without HIV to determine the overall population-level risk of exposure to the resistant virus. This is particularly important as the prevalence of resistance might not decline proportionally with reductions in overall viraemia and HIV incidence. Since viraemic people with HIV, regardless of treatment history, can transmit HIV to seronegative individuals, tracking population-level resistance trends is essential for assessing ongoing transmission risk.^1^^,^^14^

General population-based studies involving all individuals, regardless of HIV serostatus, can address these shortcomings. This design also allows for estimation of the prevalence of resistance among all participants and the relative contributions from subgroups—eg, pretreatment and treatment-experienced people with HIV. For example, a 2024 cross-sectional population-based study in KwaZulu-Natal (South Africa) found that fewer than 1% of viraemic people with HIV harboured INSTI resistance before dolutegravir treatment, but observed rtM184V (lamivudine resistance) in 32·6%, rtK65R (tenofovir disoproxil fumarate resistance) in 12·0%, and rtK70E (tenofovir disoproxil fumarate resistance) in 6·2% of treatment-experienced people with HIV.^15^ However, this study did not quantify the population prevalence of resistance among all study participants and, given the cross-sectional design, was unable to assess temporal trends in resistance among people with HIV and the general population. Longitudinal population-based cohort designs enable precise monitoring of resistance evolution and a dynamic evaluation of the risks posed to ART regimens. This design is particularly useful in the context of rapidly changing population sizes of viraemic pretreatment and treatment-experienced people with HIV observed in the past two decades during expansion of treatment and prevention programmes.^4^

We analysed HIV deep sequencing data collected as part of a general population-based cohort study in southern Uganda, the Rakai Community Cohort Study (RCCS), spanning a 9-year period of intense ART scale-up and declines in HIV incidence.^4^ Our deep-sequencing protocol allowed for the identification of minor within-individual drug resistance mutations, which can be selected for upon treatment initiation but are missed by consensus sequencing.^16^ We aimed to estimate the longitudinal prevalence of NNRTI, NRTI, and protease inhibitor (PI) resistance among the entire study population and among pretreatment or treatment-experienced people with HIV and the prevalence of resistance-conferring mutations.

## Methods

### Study design and participants

The RCCS, conducted by the Rakai Health Sciences Program (Kalisizo, Uganda), is an open population-based census and cohort study conducted at approximately 18–24-month intervals (appendix 1 pp 3–4) in agrarian (HIV prevalence 9–26%),^17^ semi-urban trading (HIV prevalence 11–21%),^17^ and Lake Victoria fishing (HIV prevalence 38–43%)^17^ communities in four districts in southern Uganda (Kyotera, Lyantonde, Rakai, and Masaka).^18^ At each survey round, households are included in the census and residents aged 15–49 years are invited to participate. Participants can contribute to each survey round in which they are eligible. Participants are administered a structured questionnaire that obtains sociodemographic, behavioural, and health information, including self-reported past and current ART use (appendix 2 pp 1–38). Voluntary HIV testing is conducted using a rapid test algorithm^19^ and a venous blood sample from participants with HIV is taken at each survey round for viral quantification and deep sequencing.

The RCCS received ethics approval from the Uganda Virus Research Institute’s Research and Ethics Committee (GC/127/08/12/137), the Uganda National Council for Science and Technology (HS540), and the Johns Hopkins School of Medicine (IRB00217467). Participants provided written informed consent (or assent for those younger than 18 years) at each survey round. This longitudinal analysis of RCCS data received ethics approval from the Johns Hopkins School of Medicine (IRB00291604).

We focused on RCCS survey, HIV viral load, and viral deep sequencing data from five survey rounds in 2012 (conducted between Aug 10, 2011, and May 29, 2013), 2014 (conducted between July 8, 2013, and Jan 28, 2015), 2015 (conducted between Feb 23, 2015, and Sept 2, 2016), 2017 (conducted between Oct 3, 2016, and May 21, 2018), and 2019 (conducted between June 19, 2018, and Nov 4, 2020). Viral load and deep sequencing data were not routinely collected in the RCCS before the 2012 survey. For participants in these rounds, we additionally included self-reported previous and current ART use from 14 survey rounds conducted between Nov 5, 1994, and June 21, 2011. Participants with serologically confirmed HIV infection were considered as pretreatment during a given round if they reported never having taken ART during that round and all previous rounds. Participants with HIV were considered as treatment-experienced during a given round if they reported having taken ART in that round or any earlier rounds. Recommended first-line ART regimens are shown in appendix 1 (p 5). Survey rounds are herein referred to by the year of the median date over which the survey was conducted (appendix 1 pp 3–4).

### Procedures

HIV viral load was measured in serum or plasma samples using the m2000 RealTime HIV-1 Quantitative PCR assay (Abbott Laboratories, Chicago, IL, USA) at the Rakai Health Sciences Program. Viral load measurements were conducted primarily among people with HIV in fishing communities in the 2012 survey round and for all people with HIV in later survey rounds. Viral loads of at least 1000 copies per mL were considered viraemic. Missing viral load measurements from pretreatment people with HIV in the 2012 round were imputed (appendix 1 pp 6–8). For missing viral load measurements in subsequent survey rounds, the observations were dropped.

Full-length HIV deep sequencing was conducted by the Phylogenetics and Networks for Generalised HIV Epidemics in Africa consortium (PANGEA-HIV; appendix 1 pp 6, 26).^20^^,^^21^ For the 2012 and 2014 surveys, and six of 2015 survey participant visits, sequencing on Illumina MiSeq and HiSeq platforms using a next-generation amplicon-based approach^22^ was attempted for participants who self-reported never having received ART and had a missing viral load or were viraemic. Nearly all viraemic participant visits from the 2015 to 2019 surveys on which sequencing was attempted, regardless of treatment status, were sequenced using veSeq-HIV, a next-generation metagenomic enrichment protocol. A random convenience sample of those archived during the 2012 and 2014 surveys from individuals who were newly diagnosed or not receiving ART (or both) that were not previously sequenced were also sequenced using this approach.

A validated pipeline, drmSEQ, was used to identify amino acid substitutions associated with reduced susceptibility to ART supported by at least ten PCR-deduplicated reads and at least 5% of reads covering a given site and predict intermediate-level or high-level drug resistance (appendix 1 pp 6, 16) and drug class resistance based on the Stanford HIV Drug Resistance Database (version 9.1).^23^^,^^24^

### Outcomes

The key outcomes were the prevalence of viraemic people with HIV with NNRTI, NRTI, PI, or multiclass resistance among all participants (regardless of HIV serostatus), which we refer to as the population prevalence, in the 2015 and 2017 surveys (appendix 1 pp 14–15). This timeframe was selected as viral sequencing was not routinely conducted for treatment-experienced people with HIV before 2015 and only data for a subset of participant visits in 2019 were available.

We also estimated the population prevalence of resistance attributed to viraemic treatment-experienced people with HIV in 2015 and 2017 and by viraemic pretreatment people with HIV between 2012 and 2019.

### Statistical analysis

Prevalence of each study outcome was estimated using Poisson regression with a log-link and robust (sandwich) SEs with survey round as a predictor variable fit with general estimating equations using geepack (version 1.3.11) to account for repeated measures and Emmeans (version 1.10.4). To provide additional context, we also estimated the prevalence of HIV, viraemic HIV (2014 and later due to missing viral load data), viraemic pretreatment HIV, and viraemic treatment-experienced HIV (2014 and later) among all study participants. We further estimated the prevalence of NNRTI, NRTI, and PI resistance and all resistance-conferring viral mutations among viraemic treatment-experienced people with HIV in 2015 and 2017 and among viraemic pretreatment people with HIV between 2012 and 2019. Given the greater prevalence of viraemia in fishing communities, among men, and among those younger than 35 years in the RCCS,^17^^,^^18^^,^^25^ we evaluated the association between these variables, using a categorical variable for age (15–24, 25–34, and 35–49 years), and resistance outcomes in adjusted analyses. Stratified estimates were generated for significantly associated covariates. We used inverse probability weighting to account for missing sequencing data among viraemic participants. 95% CIs and p values (α=0·05) were calculated using the Wald method. χ^2^ and Mann–Whitney *U* p values were calculated using the stats package in R. Data analysis and visualisation were done using tidyverse (version 2.0.0), ggplot2 (version 3.5.1), cowplot (version 1.1.3), patchwork (version 1.2.0), and ggpattern (version 1.1.1). Readxl (version 1.4.3) and haven (version 2.5.4.9) were used to parse data files (appendix 1 pp 6–19). All statistical analyses were conducted in R (version 4.4.1).

### Role of the funding source

The funders of the study had no role in study design, data collection, data analysis, data interpretation, or writing of the report.

## Results

Between Aug 10, 2011, and Nov 4, 2020, there were 43 361 participants in the RCCS and 7923 (18·27%) people with HIV. Over five survey rounds, 93 622 participant visits occurred, among which 17 460 (18·65%) were from people with HIV (table 1; appendix 1 p 20). 23 359 (53·87%) of 43 361 participants were female and 20 002 (46·13%) were male. The median age at first participation was 25 years (IQR 19–33). Over the analysis period, the median age remained similar (28 years [22–35] in 2012 and 29 years [21–38] in 2019; appendix 1 p 21); however, the age of people with HIV increased (from 32 [27–37] in 2012 to 36 years [30–42] in 2019; p<0·0001; appendix 1 p 22).

**Table 1:** Number of participant visits in each survey round stratified by HIV status

	Number of participant visits	Participant visits with HIV	Viraemic participant visits with HIV	Viraemic pretreatment participant visits with HIV	Viraemic treatment-experienced participant visits with HIV
Overall	93 622	17 460 (18·65%)	3671	4915	741
Survey round					
2012	17 167	3498 (20·38%)	··	1985	··
2014	17 992	3388 (18·83%)	1464 (43·21%)	1318 (90·03%)	146 (9·97%)
2015	19 336	3615 (18·70%)	1026 (28·38%)	822 (80·12%)	204 (19·88%)
2017	19 803	3636 (18·36%)	728 (20·02%)	502 (68·96%)	226 (31·04%)
2019	19 324	3323 (17·20%)	453 (13·63%)	288 (63·58%)	165 (36·42%)

Dates listed for each survey round represent the year of the median date over which the survey was conducted. Percentages in each category represent the percentage of participant visits in that category relative to the preceding category. Viral load data were not routinely collected for treatment-experienced people with HIV in the 2012 survey.

Viral load measurements were available for 1959 (56·00%) of 3498 people with HIV in the 2012 survey and 13 962 (99·67%) of 14 008 people with HIV in later surveys. After imputation of missing viral loads, 1985 (75·7%) of 2622 pretreatment people with HIV in the 2012 survey were classified as viraemic. Among participant visits from people with HIV in 2014–19, 3671 (26·29%) of 13 962 were contributed by viraemic people with HIV, and of those, 2930 (79·81%) were pretreatment people with HIV.

**Figure 1:**
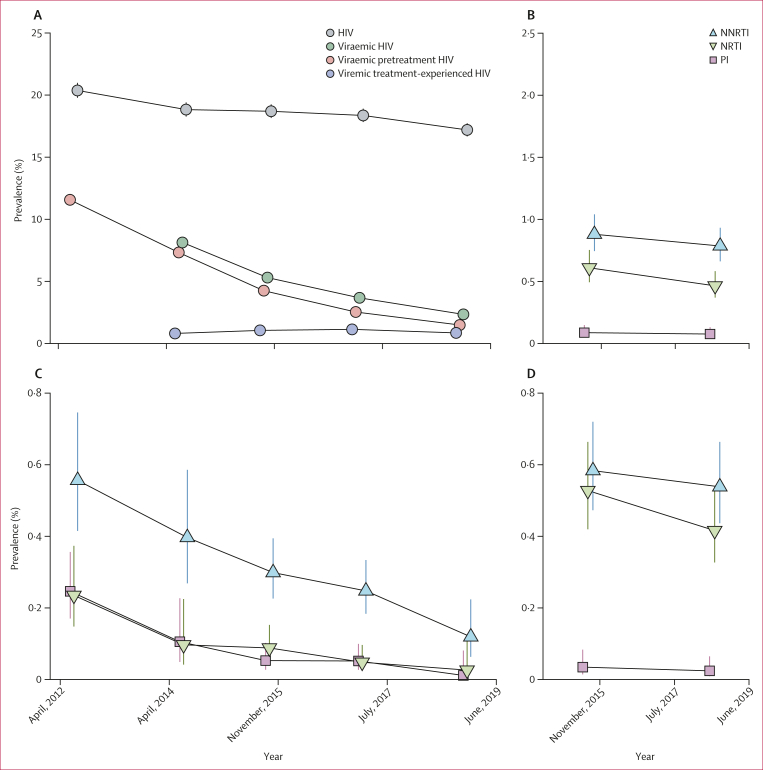
Longitudinal trends in HIV seroprevalence and population prevalence of viraemic HIV drug resistance in Rakai Community Cohort Study participants, 2012–19 Bars indicate 95% CIs. (A) Estimated prevalence of people with HIV, viraemic HIV, viraemic pretreatment HIV, and viraemic treatment-experienced HIV in each survey round. Due to missing viral load data, prevalence of viraemic HIV and viraemic treatment-experienced HIV were not estimated in the 2012 survey. Only some 95% CIs extend beyond the data point. Estimated prevalence of all people with viraemic resistance (B), viraemic pretreatment resistance (C), and viraemic treatment-experienced resistance (D) to NNRTI, NRTI, or PI. NNRTI=non-nucleoside reverse transcriptase inhibitor. NRTI=nucleoside reverse transcriptase inhibitor. PI=protease inhibitor.

Concurrent with an increase in the proportion of treatment-experienced people with HIV, the population prevalence of HIV viraemia decreased substantially from 8·14% (95% CI 7·75–8·55) in 2014 to 2·34% (2·14–2·57) in 2019. These declines were driven by an almost nine times decrease in population prevalence of viraemic pretreatment people with HIV over the study period (prevalence ratio 0·13 [0·11–0·15]) as population prevalence of viraemic treatment-experienced people with HIV remained stable at around 1%, reaching 0·85% (0·73–0·99) in 2019 (figure 1A; appendix 1 pp 23–24).

Deep sequencing identification of drug resistance mutations was attempted on 4523 (79·48%) of 5691 viraemic participant visit samples (appendix 1 pp 25–26). Sequencing for the 2019 survey was conducted only for samples collected until May 17, 2019 (171 [38%] of 453 viraemic participant visits). Among these samples, sufficient data were available to reliably genotype 4072 (90·03%) of 4523 participant visits from 3407 people with HIV for at least one drug (appendix 1 pp 27–34). Given the low numbers of intermediate-level or high-level INSTI resistance (appendix 1 pp 35–36), we did not estimate its prevalence but did estimate the prevalence of common INSTI resistance-conferring mutations, including those conferring low-level resistance.

In 2017, the population prevalence of viraemic resistance among all participants, regardless of HIV serostatus, was 0·79% (95% CI 0·66–0·93) for NNRTI, 0·46% (0·37–0·58) for NRTI, and 0·08% (0·04–0·13) for PI. These levels were stable compared with 2015 (figure 1B; appendix 1 p 37). In stratified analyses, population prevalence of NNRTI and NRTI resistance was consistently significantly higher in fishing communities than trading or agrarian communities over the analysis period (appendix 1 pp 38–40). For example, the 2017 prevalence of NNRTI resistance in fishing communities was 1·71% (1·35–2·16) versus 0·47% (0·34–0·66) in agrarian and 0·53% (0·36–0·76) in trading communities. NNRTI and NRTI resistance was also significantly lower among individuals aged 15–24 years compared with older age groups (p<0·0001 among those aged 25–34 years and p=0·00018 among those aged 35–49 years for NNRTI and p<0·0001 among both age groups for NRTI; appendix 1 pp 38–40). For example, the 2017 prevalence of NNRTI resistance was 0·33% (0·21–0·51) among those aged 15–24 years and 1·34% (1·06–1·68) among those aged 25–34 years.

**Figure 2:**
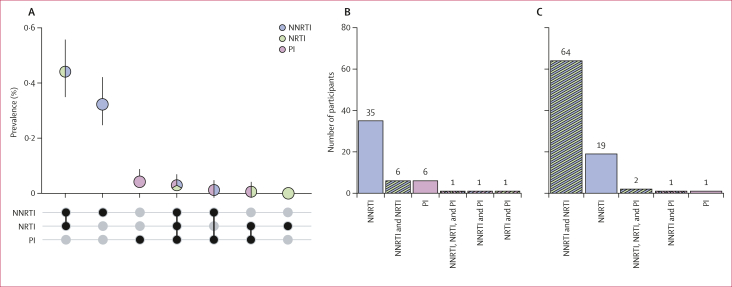
Multiclass resistance in Rakai Community Cohort Study participants, 2017 Bars indicate 95% CIs. (A) Estimated prevalence of monoresistance and multiclass resistance. (B) Multiclass resistance profiles for 50 visits of participants with viraemic pretreatment HIV with genotype data for all NNRTIs, NRTIs, and PIs and resistance to at least one of these drug classes. (C) Multiclass resistance profiles for 87 visits of participants with viraemic treatment-experienced HIV with genotype data for all NNRTIs, NRTIs, and PIs and resistance to at least one of these drug classes. NNRTI=non-nucleoside reverse transcriptase inhibitor. NRTI=nucleoside reverse transcriptase inhibitor. PI=protease inhibitor.

Overall population prevalence of resistance contributed by viraemic pretreatment people with HIV decreased between 2012 and 2017 from 0·56% (95% CI 0·42–0·75) to 0·25% (0·18–0·33) for NNRTI and from 0·24% (0·15–0·37) to 0·05% (0·02–0·10) for NRTI (prevalence ratio 0·44 [0·29–0·68] for NNRTI and 0·21 [0·09–0·47] for NRTI; figure 1C; appendix 1 pp 41–42). By 2017, viraemic treatment-experienced people with HIV contributed 68·46% (59·49–75·44) of all NNRTI and 89·61% (79·92–94·62) of all NRTI resistance (appendix 1 p 43). Resistance profiles varied considerably by treatment status (figure 2; appendix 1 p 44). Among viraemic pretreatment people with HIV with available genotypes for NNRTIs, NRTIs, and PIs, most were NNRTI monoresistant in 2017 (35 [70·00%] of 50). Whereas among viraemic treatment-experienced people with HIV with any resistance, dual-class NNRTI and NRTI resistance was the most common profile in 2017 (64 [73·56%] of 87).

**Figure 3:**
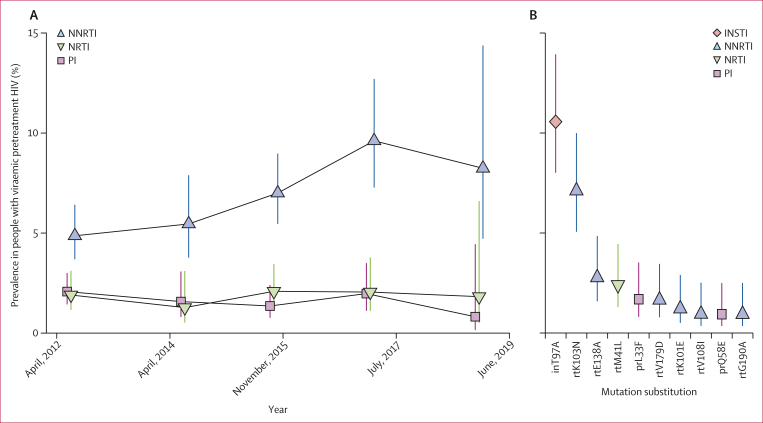
Longitudinal trends in HIV drug resistance in Rakai Community Cohort Study participants, 2012–19 Bars indicate 95% CIs. (A) Estimated prevalence of NNRTI, NRTI, and PI resistance among people with viraemic pretreatment HIV. (B) Prevalence in the 2017 survey of the ten most frequently occurring substitutions in people with viraemic pretreatment HIV. INSTI=integrase strand transfer inhibitor. NNRTI=non-nucleoside reverse transcriptase inhibitor. NRTI=nucleoside reverse transcriptase inhibitor. PI=protease inhibitor.

Between 2012 and 2017, NNRTI resistance among viraemic pretreatment people with HIV increased from 4·86% (95% CI 3·69–6·42) to 9·61% (7·27–12·7; prevalence ratio 1·98 [1·34–2·91]; figure 3A; appendix 1 pp 42, 45). This finding did not vary by sex, age, community type, or sequencing approach (appendix 1 pp 46–48). The prevalence of NRTI and PI resistance remained stable and lower than 2·1% over the same time period.

Among viraemic pretreatment people with HIV, the most prevalent resistance mutation was inT97A (figure 3B; appendix 1 pp 49–52), which is an INSTI resistance mutation (particularly for elvitegravir^26^) detected in approximately 10% of viraemic pretreatment people with HIV between 2012 and 2017. The most common NNRTI resistance mutation was rtK103N, found in 7·11% (95% CI 5·06–10·00) of viraemic pretreatment people with HIV in 2017, a significant increase compared with 2012 (prevalence ratio 4·26 [2·11–8·59]). The NNRTI resistance mutation rtE138A, which is associated with 2·5-times lower susceptibility to rilpivirine,^27^ was present in 2·77% (1·59–4·85) of viraemic pretreatment people with HIV in 2017 and its prevalence remained stable since 2012 (prevalence ratio 1·37 [0·57–3·30]). NRTI resistance mutations were rare compared with NNRTI mutations. Genotypes associated with intermediate-level or high-level INSTI resistance were identified in 16 viraemic pretreatment visits with 13 participants harbouring the mutation inE92G, which confers resistance to elvitegravir, a drug not routinely used in Uganda (appendix 1 p 5). Mutations conferring intermediate-level or high-level resistance to dolutegravir were not observed (appendix p 49).

**Figure 4:**
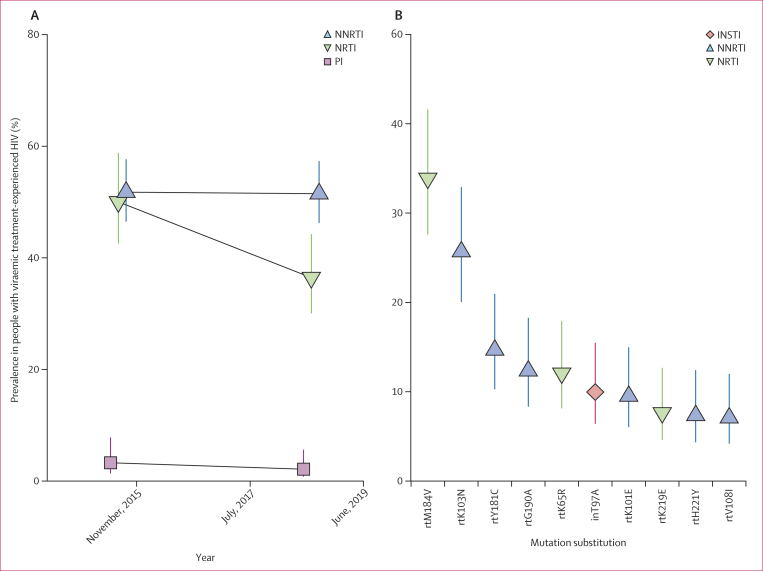
Longitudinal trends in HIV drug resistance in the Rakai Community Cohort Study participants with viraemic treatment-experienced HIV, 2015–17 Bars indicate 95% CIs. (A) Estimated prevalence of NNRTI, NRTI, and PI resistance in treatment-experienced people with viraemic HIV. (B) Prevalence of the ten most frequently occurring drug-resistance mutations in people with viraemic treatment-experienced HIV in the 2017 survey round. INSTI=integrase strand transfer inhibitor. NNRTI=non-nucleoside reverse transcriptase inhibitor. NRTI=nucleoside reverse transcriptase inhibitor. PI=protease inhibitor.

The prevalence of NNRTI and NRTI resistance was substantially higher among treatment-experienced people with HIV (51·49% [95% CI 46·24–57·34] for NNRTI and 36·46% [30·06–44·22] for NRTI in 2017) than among pretreatment people with HIV (figure 4A; appendix 1 p 53). Among treatment-experienced people, NNRTI resistance was less common among men than in women (prevalence ratio 0·74 [0·58–0·93]), and NRTI resistance was elevated among participants aged 25–34 (prevalence ratio 1·62 [1·03–2·56] versus 15–24 years; appendix 1 pp 54–55). 2·13% (0·81–5·63) of viraemic treatment-experienced people with HIV in 2017 harboured PI resistance.

Compared with pretreated people, we observed more NRTI resistance-conferring mutations among treatment-experienced people with HIV (appendix 1 pp 49, 52, 56). NRTI resistance among viraemic treatment-experienced people with HIV in 2017 was most frequently due to the rtM184V (33·89% [95% CI 27·61–41·59]), rtK65R (12·07% [8·14–17·9]), and rtK219E (7·64% [4·6–12·67]) substitutions, which were rare among pretreatment people with HIV. The most prevalent NNRTI-associated substitution among viraemic treatment-experienced people with HIV was also rtK103N (25·68% [20·03–32·92]); however, rtY181C (14·67% [10·27–20·95]) and rtG190A (12·34% [8·33–18·27]) were also frequently observed. inT97A was observed at a similar prevalence among viraemic treatment-experienced (9·96% [6·41–15·48]) and viraemic pretreatment (10·56% [8·01–13·93]) people with HIV. Four participant visit samples contributed by viraemic treatment-experienced people with HIV harboured INSTI resistance mutations, which were each observed once (inG163K, inG163R, inR263K, and inS147G) and not associated with dolutegravir resistance (appendix p 49).

## Discussion

In this study, we report on trends in HIV drug resistance from a longitudinal, population-based cohort in southern Uganda between 2012 and 2019, a period marked by the substantial expansion of ART programmes. Despite an increase in the prevalence of NNRTI resistance among pretreatment people with HIV, we observed an overall decline in the population prevalence of pretreatment HIV drug-resistant viraemia among all participants, alongside increasing ART uptake and viral suppression among people with HIV. By the end of the analysis period in 2017, the population prevalence of NNRTI resistance was 0·79% and NRTI resistance was 0·46%, with most resistance stemming from dual-class NNRTI and NRTI resistance in viraemic treatment-experienced people with HIV. Resistance to NRTIs and PIs among viraemic pretreatment people with HIV, and consequently multiclass resistance, remained low despite a substantial burden of NRTI resistance in viraemic treatment-experienced people with HIV. We observed a high prevalence of the INSTI resistance mutation inT97A. Overall, these findings provide important insights into the evolving dynamics of HIV resistance during ART scale-up in a high-burden setting of east Africa and might help to guide future surveillance and HIV epidemic control efforts in the region.

Consistent with previous studies, we observed an increase in the prevalence of NNRTI resistance among viraemic pretreatment people with HIV, supporting the 2018 shift to dolutegravir-based regimens.^1^^,^^11^^,^^12^ However, a key finding from our population-based analysis, concurrent with the rise in NNRTI resistance among pretreatment people with HIV, was a substantial decline in the overall prevalence of pretreatment people with HIV among all study participants, probably driven by increased treatment initiation and declining HIV incidence.^18^^,^^28^^,^^29^ This decrease in the prevalence of pretreatment people with HIV has outpaced the rise in NNRTI resistance among pretreatment people with HIV, resulting in a reduction in the population prevalence of pretreatment people with HIV with NNRTI resistance over the study period. By the end of the survey period, most viraemic individuals with NNRTI-resistant HIV were those with previous treatment experience.

We find a lower burden of NNRTI and NRTI resistance among viraemic treatment-experienced people with HIV in this study than that seen in clinic-based studies,^1^^,^^12^ probably because our population-based design includes people with HIV who remain viraemic since they are not actively engaged in care, despite previous treatment exposure. Clinic-based studies might disproportionately enrol people who remain viraemic due to suboptimal adherence or have advanced stages of disease, and thus are more likely to have drug resistance, whereas population-based sampling includes people lost to clinic-based care and who are no longer using treatment altogether.

NNRTI and NRTI therapies remain important components of the current and future ART landscape. Current first-line dolutegravir-regimens incorporate two NRTIs (eg, tenofovir disoproxil fumarate and lamivudine), and unsuccessful dolutegravir treatment is more likely among those with NRTI resistance.^30^^,^^31^ We observe rtM184V (>200 times lower susceptibility to lamivudine^23^^,^^24^) mutations in 33·89% and rtK65R (five times lower susceptibility to tenofovir disoproxil fumarate^23^^,^^24^) mutations in 12·07% of viraemic treatment-experienced people with HIV, whereas these mutations were rarely observed among pretreatment people with HIV. The distinct resistance profiles between these groups might be due to reduced transmission efficiency associated with resistance mutations or reversion of resistance in the absence of treatment following transmission. Further, we identify a number of mutations associated with reduced susceptibility to rilpivirine (eg, rtE138A, rtY181C, and rtG190A), an NNRTI given in combination with cabotegravir as part of long-lasting injectable therapies, in viraemic treatment-experienced people with HIV.

As this study predates the scale-up of dolutegravir, we do not observe major dolutegravir resistance-conferring mutations. We show that around 10% of viraemic participants harboured inT97A, which is a polymorphic mutation most common in subtype A and in isolation confers two-times resistance to elvitegravir but not to other INSTIs.^23^^,^^24^ The observed prevalence of inT97A in this study is an order of magnitude higher than in a population-based cohort in South Africa^15^ and about a two times higher prevalence than globally sampled INSTI-naive people with HIV.^23^^,^^24^ Further, we observe a significant increase in the prevalence of inT97A among viraemic pretreatment people with HIV in the 2019 survey round. As inT97A is repeatedly selected for in people who had unsuccessful dolutegravir therapy^32^ and can substantially increase dolutegravir resistance in combination with other mutations (eg, inG140S and inQ148H),^33^ we recommend continued monitoring.

There are important limitations of our study. Due to unknown HIV serostatus among non-participating residents of RCCS communities, our results might not be generalisable to this population. Individuals aged 15–19 years, men, and residents of trading communities are less likely to participate in RCCS surveys.^18^ Further, only self-reported treatment status was available, which might have led to the misclassification of some participants. A previous study in this cohort showed that 11% of self-reported ART-naive participants had antiviral medication present in their blood.^34^ Given the significant differences observed in the mutational profiles of pretreatment versus treatment-experienced people with HIV and the consistency of these results with estimates of the fitness impact of mutations in the absence of treatment,^35^ we expect minimal misclassification bias. In the absence of sequencing data from source-recipient pairs, we are unable to categorise the identified pretreatment resistance as transmitted drug resistance. Although data on individual-level ART regimens are not routinely collected through the RCCS, first-line therapy in this setting is highly consistent across individuals. Since this study is based on sequencing of viral RNA, we could identify resistance only among viraemic people with HIV. Consequently, our population prevalence estimates are an underestimate, as some people with resistant HIV might be suppressed via second-line therapy or were transiently suppressed after treatment initiation.

Despite the population-based study design, viral load data and sequencing data were only available for a subset of participants due to budgetary and logistical constraints. We consequently restrict analyses to survey rounds in which sufficient data are available to generate reliable inferences and use imputation to account for missing viral load data. Despite this missingness, deep sequencing data were available for 4072 participant visits, which is considerably more than a 2024 population-based study in South Africa (n=1097),^15^ clinic-based studies in sub-Saharan Africa (n=972),^12^ and WHO surveys in Uganda (n=372).^1^ Further, we used detailed demographic data on survey participants to account for the role of possible biases in sequencing data availability; however, we cannot rule out any residual biases in our estimates.

In summary, this study adds crucial context to our understanding of the HIV epidemic in southern Uganda and to the impact of treatment expansion on the population burden of HIV resistance. We show that increased viral suppression due to ART scale-up has reduced the population prevalence of pretreatment resistance and that most resistance is contributed by treatment-experienced people with HIV, which might inform interventions aimed at reducing transmitted HIV resistance. The high prevalence of NNRTI and NRTI resistance among treatment-experienced people with HIV and of inT97A among all viraemic people with HIV is potentially concerning in light of the roll-out of dolutegravir with tenofovir disoproxil fumarate plus lamivudine and cabotegravir plus rilpivirine regimens in sub-Saharan Africa. These findings stress the importance of continued viral sequence-based monitoring of resistance mutations among people with HIV, particularly those with previous treatment exposure, during the roll-out of novel HIV ART regimens and possible reductions in HIV treatment coverage.

## Data sharing

Code to reproduce all analyses and visualisations and de-identified resistance and metadata (https://github.com/m-a-martin/rccs_hiv_resistance_r15_r19). HIV consensus sequences are available from Zenodo (https://doi.org/10.5281/zenodo.10075814) and the PANGEA-HIV repository (https://github.com/PANGEA-HIV/PANGEA-Sequences). In accordance with UNAIDS guidelines, deep-sequencing reads can be requested from PANGEA-HIV at pangea.data.enquiries@ndm.ox.ac.uk. Additional cohort data can be requested from the Rakai Health Sciences Program at datarequests@rhsp.org. Further details on drmSEQ are available through PANGEA-HIV (pangea.data.enquiries@ndm.ox.ac.uk).

## Declaration of interests

We declare no competing interests.
