# Supplementary material for: HIV drug resistance during antiretroviral therapy scale-up in Uganda, 2012–19: a population-based, longitudinal study
Source: Lancet Microbe. 2025 Dec;6(12):None. doi: 10.1016/j.lanmic.2025.101218 (PMC12722182; doi:10.1016/j.lanmic.2025.101218)
Supplement: Supplementary appendix 1 [file mmc1.pdf]

THE LANCET
Microbe

Supplementary appendix 1

This appendix formed part of the original submission and has been peer reviewed. We post it as supplied by the authors.

Supplement to: Martin MA, Reynolds SJ, Foley BT, et al. HIV drug resistance during antiretroviral therapy scale-up in Uganda, 2012–19: a population-based, longitudinal study. *Lancet Microbe* 2025. https://doi.org/10.1016/j.lanmic.2025.101218

Appendix

Table of Contents

P1…Table of contents
P2…Consortia Collaborators
P3…Supplementary Table 1: Rakai Community Cohort Study interview date ranges
P4…Supplementary Figure 1: Rakai Community Cohort Study interview dates
P5…Supplementary Table 2: Recommended ART regimen among RCCS participants by year
P6-13…Supplementary methods
P14-15…Supplementary Table 3: Summary of outcome measures
P16…Supplementary Table 4: Drugs to which resistance was predicted
P17-19…Supplementary Table 5: Sampling probability model for the probability that genotyping data is available among all viraemic PLHIV
P20…Supplementary Figure 2: Number of repeat visits among RCCS participants stratified by viremia and treatment-status
P21…Supplementary Table 6: Demographics of study participants by survey round
P22…Supplementary Table 7: Demographics of participants living with HIV by survey round
P23…Supplementary Table 8: Prevalence of PLHIV, viraemic PLHIV, viraemic pretreatment PLHIV, and viraemic treatment-experienced PLHIV
P24…Supplementary Table 9: Number of participant-visits contributed by PLHIV with history of treatment
P25…Supplementary Table 10: Number of viraemic participant-visits from which deep-sequence based identification of drug resistance mutations was attempted.
P26…Supplementary Table 11: Technology used to generate sequence data by survey round
P27…Supplementary Table 12: Number of viraemic participant-visits with attempted deep-sequence based identification of drug resistance mutations that had successful genotyping for at least one drug
P28… Supplementary Table 13: Deep-sequencing quality summary statistics among participant-visits with successful genotyping for at least one drug
P29…Supplementary Table 14: Number of viraemic participant-visits with attempted deep-sequence based identification of drug resistance mutations that had successful genotyping for all INSTIs
P30…Supplementary Table 15: Number of viraemic participant-visits with attempted deep-sequence based identification of drug resistance mutations that had successful genotyping for all NNRTIs
P31…Supplementary Table 16: Number of viraemic participant-visits with attempted deep-sequence based identification of drug resistance mutations that had successful genotyping for all NRTIs
P32…Supplementary Table 17: Number of viraemic participant-visits with attempted deep-sequence based identification of drug resistance mutations that had successful genotyping for all PIs
P33…Supplementary Table 18: Number of viraemic participant-visits with attempted deep-sequence based identification of drug resistance mutations that had successful genotyping for all NRTIs, NNRTIs, and PIs
P34…Supplementary Table 19: Number of viraemic participant-visits with attempted deep-sequence based identification of drug resistance mutations that had successful genotyping for all drugs
P35-36…Supplementary Table 20: Number of viraemic participant-visits that had resistant genotypes for each drug class
P37…Supplementary Table 21: Population prevalence of viraemic NNRTI, NRTI, and PI resistance by survey round
P38…Supplementary Table 22: Association between age, community type, and sex and the population prevalence of viraemic NNRTI, NRTI and PI resistance adjusted by survey round
P39-40…Supplementary Table 23: Population prevalence of viraemic NNRTI, NRTI, and PI resistance stratified by survey round and age, community type, and sex
P41…Supplementary Table 24: Population prevalence of viraemic pretreatment NNRTI, NRTI, and PI resistance by survey round
P42…Supplementary Figure 3: Sensitivity of results to 2012 survey round viral load missingness
P43…Supplementary Table 25: Population prevalence of viraemic treatment-experienced NNRTI, NRTI, and PI resistance by survey round
P44… Supplementary Table 26: Population prevalence of viraemic multi-class resistance by survey round
P45…Supplementary Table 27: Prevalence of NNRTI, NRTI, and PI resistance among viraemic pretreatment PLHIV by survey round
P46-47…Supplementary Table 28: Association between age, community type, and sex and the prevalence of NNRTI, NRTI and PI resistance among viraemic pretreatment PLHIV adjusted by survey round
P48…Supplementary Table 29: Prevalence of NRTI resistance among viraemic pretreatment PLHIV stratified by sex
P49…Supplementary Table 30: Count of amino acid mutations observed among viraemic PLHIV
P50-51…Supplementary Table 31: Prevalence of amino acid mutations among viraemic pretreatment PLHIV by survey round
P52…Supplementary Figure 4: Within-host frequency of select resistance conferring amino acid mutations
P53…Supplementary Table 32 Prevalence of NNRTI, NRTI, and PI resistance among viraemic treatment-experienced PLHIV by survey round
P54…Supplementary Table 33: Association between age, community type, and sex and the prevalence of NNRTI, NRTI and PI resistance among viraemic treatment-experienced PLHIV adjusted by survey round
P55…Supplementary Table 34 Prevalence of NNRTI, NRTI, and PI resistance among viraemic treatment-experienced PLHIV by survey round and age, community type, and sex
P56…Supplementary Table 35: Prevalence of amino acid mutations among viraemic treatment-experienced PLHIV
P57…Supplementary references

Consortia Members

Rakai Health Sciences Program

Larry W. Chang[2,3,4,13], Ronald M. Galiwango[3], M. Kate Grabowski[1,2,3], Ronald H. Gray[1], Jade C. Jackson[1], Joseph Kagaayi[3,10], Edward Nelson Kankaka[3], Godfrey Kigozi[3], Oliver Laeyendecker[3,4,5], Thomas C. Quinn[3,4,5], Steven J. Reynolds[3,4,5], John Santelli[14], David Serwadda[3,15], Nelson K. Sewankambo[3,16], Joseph Ssekasanvu[2], Robert Ssekubugu[3], Victor Ssempijja[3,17], Maria J. Wawer[2,3], Dorean Nabukalu[3], Anthony Ndyanabo[3], Hadijja Nakawooya[3] Jessica Nakukumba[3], Grace N. Kigozi[3], Betty S. Nantume[3], Nampijja Resty[3], Jedidah Kambasu[3], Margaret Nalugemwa[3], Regina Nakabuye[3], Lawrence Ssebanobe[3], Justine Nankinga[3], Adrian Kayiira[3], Gorreth Nanfuka[3], Ruth Ahimbisibwe[3], Stephen Tomusange[3], Sarah Kalibbali[3], Margaret Nakalanzi[3], Joseph Ouma Otobi[3], Denis Ankunda[3], Joseph Lister Ssembatya[3], John Baptist Ssemanda[3], Robert Kairania[3], Emmanuel Kato[3], Alice Kisakye[3], James Batte[3], James Ludigo[3], Abisagi Nampijja[3], Steven Watya[3], Kighoma Nehemia[3], Sr. Margaret Anyokot[3], Joshua Mwinike[3], George Kibumba[3], Paschal Ssebowa[3], George Mondo[3], Francis Wasswa[3], Agnes Nantongo[3], Rebecca Kakembo[3], Josephine Galiwango[3], Geoffrey Ssemango[3], Andrew D. Redd[3,4,5], Caitlin E. Kennedy[3,13], Jennifer Wagman[18], Philip Kreniske[19,20]

PANGEA-HIV

Lucie Abeler-Dörner[9], Helen Ayles[21,22], David Bonsall[12], Rory Bowden[23,24,], Vincent Calvez[25], Myron Cohen[26,27,28], Ann Dennis[27], Tulio de Oliveira[29,30], Max Essex[31], Sarah Fidler[32], Dan Frampton[33], Christophe Fraser[9], M. Kate Grabowski[1,2,3], Tanya Golubchik[34], Ravindra Gupta[7,8], Richard Hayes[35], Joshua Herbeck[36], Anne Hoppe[37,38], Joseph Kagaayi[3,10], Pontiano Kaleebu[22,39], Paul Kellam[32], Cissy Kityo[40], Andrew Leigh Brown[41], Jairam Lingappa[41,42,43], Sikhulile Moyo[31,44], Vladimir Novitsky[45], Thumbi Ndung'u[33,46,47,48], Nick Paton[49], Deenan Pillay[33], Thomas C. Quinn[3,4,5], Andrew Rambaut[41], Oliver Ratmann[11], Janet Seeley[50], Deogratius Ssemwanga[39], Frank Tanser[48,51,52], Maria Wawer[2,3]

With thanks to George MacIntyre-Cockett[13] and Laura Thomson[53].

[1]Department of Pathology, Johns Hopkins School of Medicine, Baltimore, MD, USA; [2]Department of Epidemiology, Johns Hopkins Bloomberg School of Public Health, Baltimore, MD, USA; [3]Rakai Health Sciences Program, Kalisizo, Uganda; [4]Division of Infectious Disease, Department of Medicine, Johns Hopkins School of Medicine, Baltimore, MD, USA; [5]Division of Intramural Research, National Institute of Allergy and Infectious Diseases, National Institutes of Health, Bethesda, MD, USA; [6]Theoretical Biology and Biophysics, Los Alamos National Laboratory, Los Alamos, New Mexico, USA; [7]Department of Medicine, University of Cambridge, Cambridge, UK; [8]Africa Health Research Institute, KwaZulu-Natal, South Africa; [9]Pandemic Sciences Institute, Nuffield Department of Medicine, University of Oxford, Oxford, UK; [10]Makerere University School of Public Health, Kampala, Uganda; [11]Department of Mathematics, Imperial College London, London, England, United Kingdom; [12]Centre for Human Genetics, Nuffield Department of Medicine, University of Oxford, Oxford, UK; [13]Department of International Health, Johns Hopkins Bloomberg School of Public Health, Baltimore, MD, USA; [14]Department of Population and Family Health, Mailman School of Public Health, Columbia University, New York, NY, USA; [15]Department of Disease Control and Environmental Health Makerere University School of Public Health, Kampala, Uganda; [16]College of Health Sciences, Makere University School of Medicine, Kampala, Uganda; [17]Clinical Monitoring Research Program Directorate, Frederick National Laboratory for Cancer Research, Frederick, MD, USA; [18]Department of Community Health Sciences, Fielding School of Public Health, University of California Los Angeles, Los Angeles, USA; [19]Institute for Implementation Science in Population Health, CUNY Graduate School of Public Health and Health Policy, New York, NY, USA; [20]Department of Community Health and Social Sciences, CUNY Graduate School of Public Health and Health Policy, New York, NY, USA; [21]Zambart, University of Zambia, Ridgeway Campus, Lusaka, Zambia; [22]Department of Infectious Disease Epidemiology and International Health, The London School of Hygiene & Tropical Medicine, London, United Kingdom; [23]The Walter and Eliza Hall Institute of Medical Research, Melbourne, VIC, Australia; [24]Department of Medical Biology, University of Melbourne, Parkville, VIC, Australia; [25]Sorbonne University, INSERM, Pierre Louis Institute of Epidemiology and Public Health, AP-HP, Hôpitaux Universitaires Pitié Salpêtrière – Charles Foix, Laboratoire de Virologie, Paris, France; [26]Department of Epidemiology, Gillings School of Global Public Health, The University of North Carolina, Chapel Hill, NC, USA; [27]Division of Infectious Diseases, School of Medicine, The University of North Carolina, Chapel Hill, NC, USA; [28]Department of Microbiology and Immunology, School of Medicine, The University of North Carolina, Chapel Hill, NC, USA; [29]Centre for Epidemic Response and Innovation, School for Data Science and Computational Thinking, Stellenbosch University, Stellenbosch, South Africa; [30]KwaZulu Natal Research and Innovation Sequencing Platform, University of KwaZulu Natal, Durban, South Africa; [31]Botswana Harvard AIDS Institute Partnership, Gaborone, Botswana; [32]Department of Infectious Diseases, University College London, London, UK; [33]Division of Infection and Immunity, University College London, London, UK; [34]The Sydney Infectious Diseases Institute, School of Medical Sciences, University of Sydney, Sydney, NSW, Australia; [35]Department of Population Health, NYU Grossman School of Medicine, New York, New York, USA; [36]Gates Foundation, Seattle, WA, USA; [37]FIND, Geneva, Switzerland; [38]Elizabeth Glaser Pediatric AIDS Foundation, Geneva, Switzerland; [39]Medical Research Council/Uganda Virus Research Institute & London School of Hygiene and Tropical Medicine Uganda Unit, Entebbe, Uganda; [40]Joint Clinical Research Centre/Kampala CRS, Kampala, Uganda; [41]Institute of Evolutionary Biology, The University of Edinburgh, Edinburgh, UK; [41]Department of Global Health, University of Washington, Seattle, WA, USA; [42]Department of Medicine, University of Washington, Seattle, WA, USA; [43]Department of Pediatrics, University of Washington, Seattle, WA, USA; [44]Harvard T.H. Chan School of Public Health, Boston, MA, USA; [45]Department of Medicine, Alpert Medical School of Brown University, Providence, RI, USA; [46]Ragon Institute of Mass General, MIT and Harvard, Cambridge, MA, USA; [47]HIV Pathogenesis Programme, the Doris Duke Medical Research Institute, UKZn, Durban, South Africa; [48]Africa Health Research Institute, Durban, South Africa; [49]Yong Loo Lin School of Medicine, Singapore, Singapore; [50]Department of Global Health and Development, London School of Hygiene and Tropical Medicine, London, UK; [51]Centre for Epidemic Response and Innovation, School of Data Science and Computational Thinking, Stellenbosch University, Stellenbosch, South Africa; [52]South African Centre for Epidemiological Modelling and Analysis, School of Data Science and Computational Thinking, Stellenbosch University, Stellenbosch, South Africa; [53]Big Data Institute, Li Ka Shing Centre for Health Information and Discovery, Nuffield Department of Medicine, University of Oxford, Oxford, UK.

Supplementary Table 1: Rakai Community Cohort Study survey dates

Round	Survey start date	Survey mid date	Survey end date
1	1994-11-05	1995-03-20	1995-07-18
2	1995-01-12	1996-01-10	1996-05-01
3	1996-01-12	1996-11-20	1997-04-23
4	1997-06-06	1997-11-21	1998-05-12
5	1997-08-05	1998-08-06	1999-03-15
6	1999-04-06	1999-09-07	2000-02-09
7	2000-03-20	2000-08-08	2001-02-19
8	2001-04-03	2001-10-23	2002-05-31
9	2002-07-15	2003-01-28	2003-08-01
10	2003-09-26	2004-04-23	2004-11-23
11	2005-01-13	2005-10-13	2006-06-30
12	2006-03-31	2007-06-28	2008-04-24
13	2008-06-17	2009-03-17	2009-12-04
14	2010-01-18	2010-09-24	2`011-06-21
15	2011-08-10	2012-04-02	2013-05-29
16	2013-07-08	2014-04-07	2015-01-28
17	2015-02-23	2015-11-03	2016-09-02
18	2016-10-03	2017-07-17	2018-05-21
19	2018-06-19	2019-06-20	2020-11-04

Start, mid (median), and end dates for each RCCS survey round. Viral load and sequencing data used in this study were collected during R15-19. R19 sequence data was generated for participant visits through 2019-05-17.

Supplementary Figure 1: Rakai Community Cohort Study interview dates

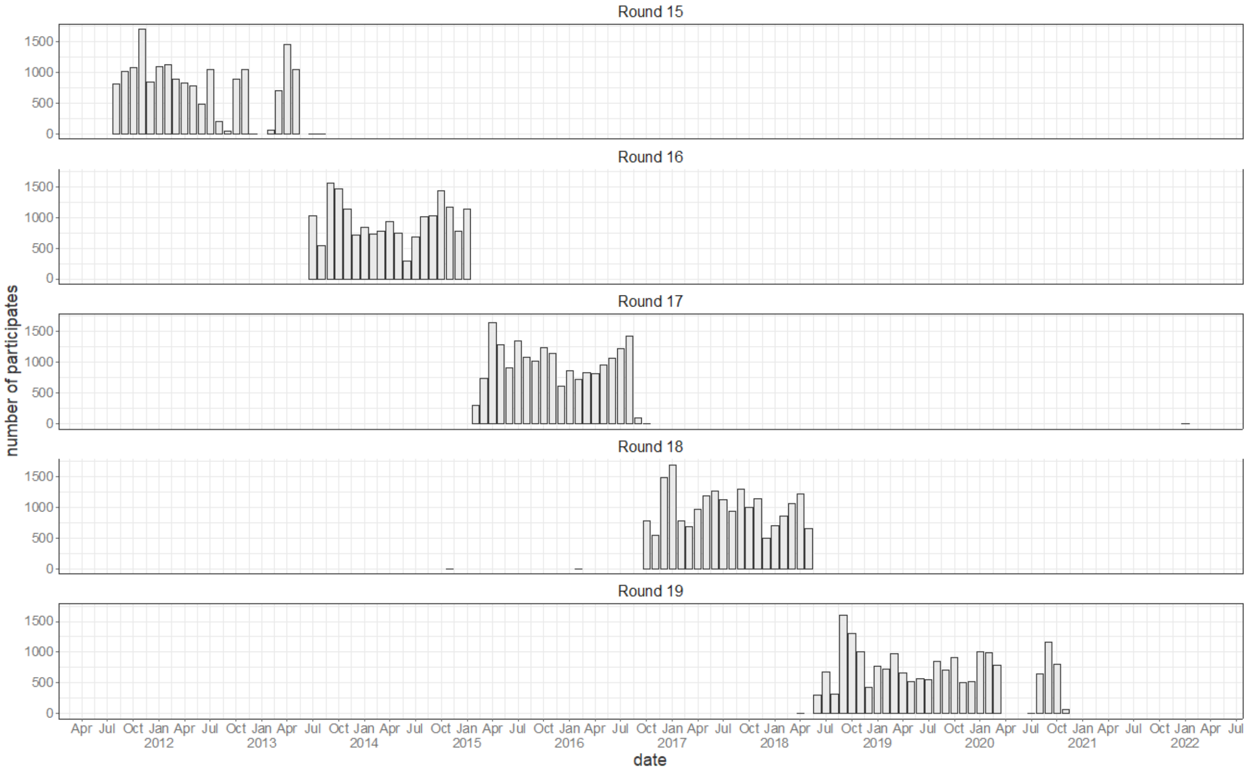

Number of interviews conducted each month, stratified by Rakai Community Cohort Study round.

Supplementary Table 2: Recommended ART regimen among RCCS participants by year

Year	First line regimen	Second line regimen
	**Preferred**	**Preferred**
	TDF/3TC + NVP	AZT + 3TC + ATV/r
	TDF/3TC + EFV	AZT + 3TC + LPV/r
2012		
	**Alternative**	**Alternative**
	AZT/3TC + NVP	TDF + 3TC + ATV/r
	AZT/3TC + EFV	TDF + 3TC + LPV/r
	**Preferred**	**Preferred**
	TDF+3TC+EFV	2 NRTIs and ritonavir-boosted atazanavir (ATV/r)
		e.g.
2016	**Alternative (only if EFV is contraindicated)**	After failing on TDF + 3TC or ABC+3TC based regimen: AZT+3TC / ATV/r
	TDF+3TC+DTG	
		Αφτερ φαιλινγ ον AZT+3TX βασεδ ρεγιμεν: ΤΔΦ + 3ΤΧ/ ΑΤς/ρ
	**Preferred**	**Preferred**
	TDF+3TC+DTG	AZT+3TC+ATV/r
	**Alternative**	**Alternative**
	TDF+3TC+EFV, ABC+3TC+DTG	AZT+3TC+LPV/r
		AZT+3TC+DTG
2018	**Adult women and adolescent girls of childbearing potential who are pregnant, intend to get pregnant or not on effective contraception**	
	TDF+3TC+EFV	
	**Alternative**	
	TDF+3TC+ATVr or ABC+3TC+EFV	
	**Preferred**	**Preferred**
	TDF + 3TC + DTG	AZT+3TC+ATV/r
		TDF+3TC+ATV/r
	**Alternatives**	
2022	TDF + 3TC + EFV400	**Alternative**
	ABC + 3TC +DTG	**AZT+3TC+LPV/r**
	ABC +3TC +EFV400	**TDF+3TC+LPV/r**
	TDF +3TC + ATV/r or ABC	
	3TC + ATV/r	

Supplementary Methods

HIV deep sequencing

As described elsewhere,[1] for samples taken at participant visits during the 2012 and 2014 survey rounds RNA extraction was performed at University College London Hospital (London, United Kingdom) using the QIAsymphony SP workstation with the QIAsymphony DSP Virus/Pathogen Kit (Qiagen, Hilden, Germany). cDNA from four overlapping amplicons covering the HIV-1 genome was generated using in a one-step reverse-transcription protocol using universal primers.[2] Following gel electrophoresis confirmation of amplification samples were sequenced at the Wellcome Trust Sanger Institute (Hinxton, United Kingdom) on Illumina MiSeq and HiSeq platforms.

For the remaining survey rounds and select participant-visits from earlier rounds, sequencing was performed using the veSeq-HIV protocol. VeSeq-HIV uses oligo-nucleotide bait enrichment of HIV from pooled metagenomic libraries prepared without virus-specific PCR. RNA extraction was performed as above at the Oxford Genomics Centre (Oxford, United Kingdom) followed by library preparation with the SMARTer Stranded Total RNA-Seq kit v2 – Pico Input Mammalian (Clontech, TakaRa Bio). Streptavidin-conjugated beads were used to isolate fragments shorter than 400 nucleotides and PCR amplification and purification was performed with the Agencourt AMPure XMP (Beckman Coulter) following the veSEQ-HIV protocol.[3] The Oxford Genomics Centre generated 350 to 600 base pair reads using the Illumina NovaSeq 6000 instrument.

Our high-throughput implementation of the veSEQ-HIV protocol incorporates a HIV plasma negative control with each batch of 81 samples, as well as a serial dilution of HXB2 cultured virus diluted in pooled human plasma from donors testing negative for HIV ($1e^5$, $1e^4, 1e^3, 2e^2$) as a quantitative reference and for quality control. The veSEQ-HIV protocol minimizes contamination by double-indexing samples and controls before PCR amplification, at an early stage of our simplified single-tube library preparation. Within-run contaminations that occur post-PCR are, therefore, eliminated by the demultiplexing. Contaminations occurring pre-PCR are monitored by detection of HIV reads in the- negative plasma control, and by detection of HXB2 reads in any sample that is not a quantitative standard. Between-run contamination events are monitored using the phyloscanner pipeline, which characterizes within- and between- sample phylogenies of the viral quasi-species, and which identifies contaminants as reads divergent from the sample consensus and identical to reads from other samples from previous sequencing runs. Contaminant read counts by these detection methods never exceeded 5 reads at any single point on the HIV genome, and therefore never exceeded our thresholds for calling drug resistant mutations, even for low viral load samples. Sequencing quality was monitored from total counts of HIV reads detected in the quantitative controls, their PCR duplication rates and median insert sizes. Where contaminations were detected either by the presence of HIV reads in the negative control, or HXB2 reads present in the samples, sequencing runs were repeated.

Sequencing reads were filtered with trimmomatic[4] to remove any reads with less than 50 bases, remove leading and trailing bases if their quality score was below 20, and cut reads when the average quality score in a 4 base sliding window was less than 20. Reads were filtered to remove *pol* hypermutated sequences and non-HIV *pol* sequences. PCR duplicate reads were removed using Picard MarkDuplicates.[5]

Identification of drug resistance mutations

PCR deduplicated reads were locally aligned to 142 HIV subtype references using blastx.[6] A manually-curated codon-restricted multiple-alignment of the references was used to reconcile coordinates with HXB2 (GenBank: K03455.1). Only mutations supported by a ≥10 PCR-deduplicated reads and by ≥5% of reads spanning the corresponding site were considered.[7] The same thresholds demonstrated comparable sensitivity to a gold standard assay in a validation study.[8] Amino acid substitutions were scored according to the Stanford University HIV Drug Resistance Database.[9–11] Scores were summed to predict susceptibility to 25 HIV drugs. A score ≥30 (intermediate/high-level) for a given drug was categorized as resistant. Resistance was not predicted if ≥50% of the relevant positions for a given drug had <10 reads. Samples in which there was insufficient sequencing coverage for ≥1 drug within a class were not assigned a resistance categorization for that class. Samples with resistance to ≥1 drug within a class were categorized as resistant.

Data attributes

For each participant-visit i, we assign the following attributes:

- S_i: Categorical variable indicating which survey round participant-visit i was contributed towards
- H_i: Binary variable indicating whether participant-visit i was contributed by a person living with HIV (PLHIV) as determined by a validated rapid-test algorithm (*Methods*).
- Q_i: Binary variable indicating whether a viral load measurement is available for participant-visit i. Among samples with $Q_i = 1$, viral loads were measured using the Abbott real-time m2000 assay (Abbott Laboratories).
- V_i: binary variable indicating whether participant-visit i is both seropositive and viraemic. Among participant-visits with an available viral load measurement, viremia was defined as ≥ 1000 copies/mL. Viral load measurements were routinely conducted for PLHIV in the 2014 through 2019 survey rounds and missing data is minimal (see: *Results*). In the 2012 survey round, viral load measurements were only available for residents of Fishing communities. We impute missing 2012 survey viral loads among pretreatment PLHIV (*see below).* Treatment-experienced participant-visits contributed to the 2012 survey round are not considered. Assumed to be 0 for all i with $H_i = 0$ or $Q_i = 0$.
- T_i: binary variable indicating whether participant-visit i is treatment experienced. Treatment-experienced is defined as having reported being on treatment during the survey round that participant visit i was contributed to or any earlier survey rounds.
- R_i^c: binary variable indicating whether participant-visit i has available resistance prediction to drug class c (NNRTI, NRTI, PI, joint NNRTI/NRTI/PI, and joint INSTI/NNRTI/NRTI/PI). Additionally, R_i^{any} indicates available resistance prediction to at least one drug (i.e. exclude sequenced samples with insufficient coverage to predict resistance for all included drugs). Assumed to be 0 for all i with $V_i = 0$ or $H_i = 0$.
- D_i^c: binary variable indicating whether participant-visit i has predicted viraemic resistance to drug class c. Assumed to be 0 for all i with $V_i = 0$ or $H_i = 0$.
- $M_{m,i}$: binary vector for each participant-visit indicating presence/absence of each resistance conferring mutation m.

We note that individual participants can contribute up to one participant-visit per survey round, conditional on being a resident of a participating community during that survey.

Imputation of missing round 2012 pretreatment viral loads

Viral load measurements were conducted for only a subset of participants in the 2012 survey round. We here focus on participant-visits contributed by viraemic PLHIV. Consequently, to avoid discarding a large proportion of 2012 participant-visits we impute V_i for all pretreatment i contributed to the 2012 survey round with $Q_i = 0$. As we do not attempt to estimate the prevalence of viraemic treatment-experienced resistance in the 2012 survey round we do not do any imputation with for those i with $Q_i = 0, T_i = 1$.

Specifically, because sequencing success depends on sample viral load[3], we impute missing V_i based on observed V_i among 2012 survey participants with viral load measurements conditional on availability of resistance prediction to at least one drug (R_i^{any}).

We first calculate:

$$P(V = 1 | R^{any} = 1, Q = 1, S = 2012) = \frac{\sum_{i \in 2012} R_i^{any} V_i Q_i}{\sum_{i \in 2012} V_i Q_i}$$

$$P(V = 1 | R^{any} = 0, Q = 1, S = 2012) = \frac{\sum_{i \in 2012} (\sim R_i^{any}) V_i Q_i}{\sum_{i \in 2012} V_i Q_i}$$

Where $\sim R_i^{any}$ indicates "not R_i^{any}".

And then impute missing viral loads as:

$$\left(V_i | R_i^{any} = 1, Q_i = 0, S = 2012\right) \sim \text{Bernoulli}(P(V = 1 | R^{any} = 1, Q = 1, S = 2012))$$

$$\left(V_i \middle| R_i^{any} = 0, Q_i = 0, S = 2012\right) \sim \text{Bernoulli}(P(V = 1 | R^{any} = 0, Q = 1, S = 2012))$$

Sampling probabilities
For all outcome measures that relied on sequence-based resistance predictions we used inverse probability weighting to account for the fact that sequence data was not available for all participant-visits contributed by viraemic PLHIV and that resistance prediction was unsuccessful for some sequenced participant-visits. First, among all viraemic participant-visits ($V_i = 1$) we estimate the probability that resistance prediction is available for each drug class (c, NNRTI, NRTI, PI individually and composite NNRTI&NRTI&PI (NNP for short) and INSTI&NNRTI&NRTI&PI (INNP for short)) based on epidemiological covariates (X_i). This analysis is stratified by survey round, s.

We define $\pi_i^{s,c}$ as the probability that resistance prediction to class c is available for participant-visit i conditional on i being contributed to survey round s and $V_i = 1$. In mathematical notation: $\pi_i^{s,c} = P(R^c = 1 | X_i, V_i = 1, H_i = 1, S_i = s)$. We estimate $\pi_i^{s,c}$ and all β using robust Poisson regression[12] for each survey round (s):

$$\log\left(\frac{\pi_i^{s,c}}{1 - \pi_i^{s,c}}\right) = \beta_0^{s,c} + \beta_1^{s,c} X_i^{\text{treatment-experienced}} + \beta_2^{s,c} X_i^{\text{missing viral load}} + \beta_3^{s,c} X_i^{\text{viral load}} + \beta_4^{rsc} X_i^{\text{trading}} + \beta_5^{s,c} X_i^{\text{fishing}} + \beta_6^{s,c} X_i^{\text{25-34 yrs.}} + \beta_7^{s,c} X_i^{\text{35-49 yrs.}} + \beta_8^{s,c} X_i^{\text{male}} + \varepsilon_i$$

Where X_i^j are binary indicator variables with the exception of $X_i^{viral\ load}$, which is the $\log_{10}$ viral load (copies/mL) for participant-visit i and is 0 for all i with missing viral loads ($X_i^{\text{missing viral load}} = 1$). Sampling probability model estimates are provided in table S5 (appendix pp 14-16).

Sampling weights are calculated based on sampling probabilities for each outcome of interest depending on the population under study, as described below.

Outcome measures
A summary of primary outcome measures and corresponding figure references is provided in Supplementary Table 3 (appendix pp 13-14). Outcomes below are listed in order of their appearance in the *Results* section.

We estimate each quantity using a robust Poisson regression with survey round as a predictor variable using general estimating equations (GEE) to account for repeated measures within participants. The correlation structure that minimizes Quasi Information Criterion (QIC) for each outcome was chosen. Estimates stratified by epidemiological covariates used the correlation structure from the corresponding non-stratified analysis.

Population prevalence of all PLHIV, viraemic PLHIV, viraemic pretreatment PLHIV, and viraemic treatment-experienced PLHIV among study participants
Here, the population under study is all contributed participant-visits. The quantities of interest are the prevalence of all PLHIV, all viraemic PLHIV, all viraemic pretreatment PLHIV, and viraemic treatment-experienced PLHIV among study participants in each survey round s. In mathematical notation, we estimate the following quantities (q) for all s:

- Prevalence of all PLHIV: $P(H = 1 | S = s)$
- Prevalence of viraemic PLHIV: $P(V = 1, H = 1 | S = s)$
- Prevalence of viraemic pretreatment PLHIV: $P(T = 0, V = 1, H = 1 | S = s)$
- Prevalence of viraemic treatment-experienced PLHIV: $P(T = 1, V = 1, H = 1 | S = s)$

Due to missing viral loads, particularly among treatment-experienced PLHIV, we did not attempt to estimate the prevalence of viraemic PLHIV or the prevalence of viraemic treatment-experienced PLHIV in the 2012 survey round.

As these outcomes did not depend on the availability to sequence-based resistance prediction, inverse probability weighting was not employed.

Population prevalence of viraemic NNRTI, NRTI, and PI resistance among study participants

Here, the population under study is all contributed participant-visits. The quantities of interest are the prevalence of NNRTI, NRTI, and PI resistance among study participants in each survey round s. We estimate the following quantities for all s:

- Prevalence of viraemic NNRTI resistance: $P(D^{NNRTI} = 1, V = 1, H = 1 | S = s)$
- Prevalence of viraemic NRTI resistance: $P(D^{NRTI} = 1, V = 1, H = 1 | S = s)$
- Prevalence of viraemic PI resistance: $P(D^{PI} = 1, V = 1, H = 1 | S = s)$

Due to missing sequence-data among treatment-experienced PLHIV in the 2012, 2014, and 2019 survey rounds this analysis was restricted to the 2015 and 2017 rounds. Bivariate models were used to evaluate whether age category, sex, community type, or treatment status was associated with each outcome. Significant covariates were further analyzed in stratified analysis.

Viraemic participant-visits lacking resistance prediction were dropped and viraemic participant-visits with resistance prediction were upweighted accordingly using inverse probability weighting. Specifically, participant-visits in round s were assigned weight for resistance class c ($w_i^{s,c}$) according to:

$$w_i^{s,c} = \begin{cases} 1, & H_i = 0 \\ 1, & V_i = 0, H_i = 1 \\ 0, & R_i^c = 0, V_i = 1, H_i = 1 \\ \left(\sum_{j \in s} V_j H_j\right) \dfrac{\frac{1}{\pi_i^{s,c}}}{\sum_{j \in s}\left(R_j^c \frac{1}{\pi_j^{s,c}}\right)}, & R_i^c = 1, V_i = 1, H_i = 1 \end{cases}$$

Where $i \in s$ indicates all participant-visits contributed to round s. Normalizing by the sum of all weights contributed to round s and multiplying by the total number of viraemic PLHIV in round s ensures that the sum of the weights is equal to the total number of viraemic PLHIV in round s ($\sum_{i \in s} w_i^{s,c} = \sum_{i \in s} V_i H_i$).

Population prevalence of viraemic pretreatment resistance among study participants

Here, the population under study is all contributed participant-visits. The quantity of interest is the prevalence of NNRTI, NRTI, and PI viraemic pretreatment resistance among study participants in each survey round s. We estimate the following quantities for all s:

- Prevalence of viraemic pretreatment NNRTI resistance: $P(D^{NNRTI} = 1, T = 0,\ V = 1, H = 1 | S = s)$
- Prevalence of viraemic pretreatment NRTI resistance: $P(D^{NRTI} = 1, T = 0,\ V = 1, H = 1 | S = s)$
- Prevalence of viraemic pretreatment PI resistance: $P(D^{PI} = 1, T = 0,\ V = 1, H = 1 | S = s)$

Viraemic pretreatment participant-visits lacking resistance prediction were dropped and viraemic pretreatment participant-visits with resistance prediction were upweighted accordingly using inverse probability weighting. Viraemic treatment-experienced participant-visits were assigned a weight of 1. Specifically, participant-visits in round s were assigned weight for resistance class c ($w_i^{s,c}$) according to:

$$w_i^{s,c} = \begin{cases} 1, & H_i = 0 \\ 1, & V_i = 0, H_i = 1 \\ 1, & T_i = 1, V_i = 1, H_i = 1 \\ 0, & R_i^c = 0, T_i = 0, V_i = 1, H_i = 1 \\ \left(\sum_{j \in s} ((\sim T_j) V_j H_j)\right) \dfrac{\frac{1}{\pi_i^{s,c}}}{\sum_{j \in s}\left((\sim T_j) R_j^c \frac{1}{\pi_j^{s,c}}\right)}, & R_i^c = 1, T_i = 0, V_i = 1, H_i = 1 \end{cases}$$

Where $\sim T_j$ is 1 when $T_j = 0$.

Population prevalence of viraemic treatment-experienced resistance among study participants
Here, the population under study is all contributed participant-visits. The quantity of interest is the prevalence of NNRTI, NRTI, and PI viraemic treatment-experienced resistance among study participants in each survey round s. We estimate the following quantities for all s:

- Prevalence of viraemic treatment-experienced NNRTI resistance:
  $P(D^{NNRTI} = 1, T = 1, V = 1, H = 1 | S = s)$
- Prevalence of viraemic treatment-experienced NRTI resistance:
  $P(D^{NRTI} = 1, T = 1, V = 1, H = 1 | S = s)$
- Prevalence of viraemic treatment-experienced PI resistance:
  $P(D^{PI} = 1, T = 1, V = 1, H = 1 | S = s)$

Due to missing sequence-data among treatment-experienced PLHIV in the 2012, 2014, and 2019 survey rounds this analysis was restricted to the 2015 and 2017 rounds.

Viraemic treatment-experienced participant-visits lacking resistance prediction were dropped and viraemic treatment-experienced participant-visits with resistance prediction were upweighted accordingly using inverse probability weighting. Viraemic treatment-experienced participant-visits were assigned a weight of 1. Specifically, participant-visits in round s were assigned weight for resistance class c ($w_i^{s,c}$) according to:

$$w_i^{s,c} = \begin{cases} 1, & H_i = 0 \\ 1, & V_i = 0, H_i = 1 \\ 1, & T_i = 0, V_i = 1, H_i = 1 \\ 0, & R_i^c = 0, T_i = 1, V_i = 1, H_i = 1 \\ \left(\sum_{j \in s} T_j V_j H_j\right) \dfrac{\frac{1}{\pi_i^{s,c}}}{\sum_{j \in s}\left(T_j R_j^c \frac{1}{\pi_j^{s,c}}\right)}, & R_i^c = 1, T_i = 1, V_i = 1, H_i = 1 \end{cases}$$

Population prevalence of viraemic multi-class resistance among study participants
Here, the population under study is all contributed participant-visits. The quantity of interest is the prevalence of combinations of multi-class NNRTI, NRTI, and PI resistance among study participants in each survey round s. We estimate the following quantities for all s:

- Prevalence of viraemic NNRTI mono-resistance:
  $P(D^{NNRTI} = 1, D^{NRTI} = 0, D^{PI} = 0, V = 1, H = 1 | S = s)$
- Prevalence of viraemic NRTI mono-resistance:
  $P(D^{NNRTI} = 0, D^{NRTI} = 1, D^{PI} = 0, V = 1, H = 1 | S = s)$
- Prevalence of viraemic PI mono-resistance:
  $P(D^{NNRTI} = 0, D^{NRTI} = 0, D^{PI} = 1, V = 1, H = 1 | S = s)$
- Prevalence of viraemic NNRTI/NRTI dual-resistance:
  $P(D^{NNRTI} = 1, D^{NRTI} = 1, D^{PI} = 0, V = 1, H = 1 | S = s)$
- Prevalence of viraemic NNRTI/PI dual-resistance:
  $P(D^{NNRTI} = 1, D^{NRTI} = 0, D^{PI} = 1, V = 1, H = 1 | S = s)$
- Prevalence of viraemic NRTI/PI dual-resistance:
  $P(D^{NNRTI} = 0, D^{NRTI} = 1, D^{PI} = 1, V = 1, H = 1 | S = s)$
- Prevalence of viraemic triple-resistance:
  $P(D^{NNRTI} = 1, D^{NRTI} = 1, D^{PI} = 1, V = 1, H = 1 | S = s)$

Due to missing sequence-data among treatment-experienced PLHIV in the 2012, 2014, and 2019 survey rounds this analysis was restricted to the 2015 and 2017 rounds.

Viraemic participant-visits lacking resistance prediction were dropped and viraemic participant-visits with resistance prediction were upweighted accordingly using inverse probability weighting. For all quantities, we use the sampling probabilities calculated based on the availability of resistance prediction to NNRTIs, NRTI and PIs, $\pi_i^{s,NNP}$. Specifically, participant-visits in round s were assigned weights ($w_i^{s,NNP}$) according to:

$$w_i^{s,NNP} = \begin{cases} 1, & H_i = 0 \\ 1, & V_i = 0, H_i = 1 \\ 0, & R_i^{NNP} = 0, V_i = 1, H_i = 1 \\ \left(\sum_{j\in s} V_i H_i\right) \dfrac{\frac{1}{\pi_i^{s,NNP}}}{\sum_{j\in s}\left(R_j^{NNP}\frac{1}{\pi_j^{s,NNP}}\right)}, & R_i^{NNP} = 1, V_i = 1, H_i = 1 \end{cases}$$

Prevalence of resistance among viraemic pretreatment participants
Here, the population under study is viraemic pretreatment participant-visits (all i with $T_i = 0, V_i = 1, H_i = 1$). The quantity of interest is the prevalence of NNRTI, NRTI, and PI resistance among viraemic pretreatment participants in each survey round s. We estimate the following quantities for all s:

- Prevalence of NNRTI resistance among viraemic pretreatment participant-visits: $P(D^{NNRTI} = 1 \,| T = 0, V = 1, H = 1, S = s)$
- Prevalence of NRTI resistance among viraemic pretreatment participant-visits: $P(D^{NRTI} = 1 \,| T = 0, V = 1, H = 1, S = s)$
- Prevalence of PI resistance among viraemic pretreatment participant-visits: $P(D^{PI} = 1 \,| T = 0, V = 1, H = 1, S = s)$

We additionally estimate each quantity stratified by sex, age category, and community type. Bivariate models were used to evaluate whether age category, sex, or community type was associated with each outcome. Significant covariates were further analyzed in stratified analysis.

Viraemic pretreatment participant-visits lacking resistance prediction were dropped and viraemic pretreatment participant-visits with resistance prediction were upweighted accordingly using inverse probability weighting. Specifically, participant-visits in round s were assigned weight for resistance class c ($w_i^{s,c}$) according to:

$$w_i^{s,c} = \begin{cases} 0, & H_i = 0 \\ 0, & V_i = 0, H_i = 1 \\ 0, & T_i = 1, V_i = 1, H_i = 1 \\ 0, & R_i^c = 0, T_i = 0, V_i = 1, H_i = 1 \\ \left(\sum_{i\in s}\left((\sim T_i) V_i H_i\right)\right) \dfrac{\frac{1}{\pi_i^{s,c}}}{\sum_{i\in s}\left((\sim T_i) R_i^c \frac{1}{\pi_i^{s,c}}\right)}, & R_i^c = 1, T_i = 0, V_i = 1, H_i = 1 \end{cases}$$

Prevalence of resistance conferring mutations among viraemic pretreatment participants
Here, the population under study is viraemic pretreatment participant-visits (all i with $T_i = 0, V_i = 1, H_i = 1$). The quantity of interest is the prevalence of each resistance mutations m among viraemic pretreatment participants in each survey round s. For each mutation, we estimate the following quantity for all s:

- Prevalence of m among viraemic pretreatment participant-visits: $P(M_m = 1 | T = 0, V = 1, H = 1, S = s)$

Viraemic pretreatment participant-visits lacking resistance prediction were dropped and viraemic pretreatment participant-visits with resistance prediction were upweighted accordingly using inverse probability weighting. For all mutations, we use the sampling probabilities calculated based on the availability of resistance prediction to

INSTIs, NNRTIs, NRTIs, and PIs, $\pi_i^{s,INNP}$. Specifically, participant-visits in round s were assigned weights ($w_i^{s,INNP}$) according to:

$$w_i^{s,INNP} = \begin{cases} 0, & H_i = 0 \\ 0, & V_i = 0, H_i = 1 \\ 0, & T_i = 1, V_i = 1, H_i = 1 \\ 0, & R_i^{INNP} = 0, T_i = 0, V_i = 1, H_i = 1 \\ \left(\sum_{j \in s} \left((\sim T_j) V_j H_j\right)\right) \dfrac{\frac{1}{\pi_i^{s,INNP}}}{\sum_{j \in s}\left((\sim T_j) R_j^{INNP} \frac{1}{\pi_j^{s,INNP}}\right)}, & R_i^{INNP} = 1, T_i = 0, V_i = 1, H_i = 1 \end{cases}$$

Prevalence of resistance among viraemic treatment-experienced participants
Here, the population under study is viraemic treatment-experienced participant-visits (all i with $T_i = 1, V_i = 1, H_i = 1$). The quantity of interest is the prevalence of NNRTI, NRTI, and PI resistance among viraemic treatment-experienced participants in each survey round s. We estimate the following quantities for all s:

- Prevalence of NNRTI resistance among viraemic treatment-experienced participant-visits: $P(D^{NNRTI} = 1 \mid T = 1, V = 1, H = 1, S = s)$
- Prevalence of NRTI resistance among viraemic treatment-experienced participant-visits: $P(D^{NRTI} = 1 \mid T = 1, V = 1, H = 1, S = s)$
- Prevalence of PI resistance among viraemic treatment-experienced participant-visits: $P(D^{PI} = 1 \mid T = 1, V = 1, H = 1, S = s)$

Due to missing sequence-data among treatment-experienced PLHIV in the 2012, 2014, and 2019 survey rounds this analysis was restricted to the 2015 and 2017 rounds. Bivariate models were used to evaluate whether age category, sex, or community type was associated with each outcome. Significant covariates were further analyzed in stratified analysis.

Viraemic treatment-experienced participant-visits lacking resistance prediction were dropped and viraemic treatment-experienced participant-visits with resistance prediction were upweighted accordingly using inverse probability weighting. Specifically, participant-visits in round s were assigned weight for resistance class c ($w_i^{s,c}$) according to:

$$w_i^{s,c} = \begin{cases} 0, & H_i = 0 \\ 0, & V_i = 0, H_i = 1 \\ 0, & T_i = 0, V_i = 1, H_i = 1 \\ 0, & R_i^c = 0, T_i = 1, V_i = 1, H_i = 1 \\ \left(\sum_{j \in s} \left(T_j V_j H_j\right)\right) \dfrac{\frac{1}{\pi_i^{s,c}}}{\sum_{j \in s}\left(T_j R_j^c \frac{1}{\pi_j^{s,c}}\right)}, & R_i^c = 1, T_i = 1, V_i = 1, H_i = 1 \end{cases}$$

Prevalence of resistance conferring mutations among viraemic treatment-experienced participants
Here, the population under study is viraemic treatment-experienced participant-visits (all i with $T_i = 1, V_i = 1, H_i = 1$). The quantity of interest is the prevalence of each resistance mutation m among viraemic pretreatment participants in each survey round s. For each mutation, we estimate the following quantity for all s:

- Prevalence of m among viraemic treatment-experienced participant-visits:
  $P(M_m = 1 | T = 1, V = 1, H = 1, S = s)$

Due to missing sequence-data among treatment-experienced PLHIV in the 2012, 2014, and 2019 survey rounds this analysis was restricted to the 2015 and 2017 rounds.

Viraemic treatment-experienced participant-visits lacking resistance prediction were dropped and viraemic treatment-experienced participant-visits with resistance prediction were upweighted accordingly using inverse probability weighting. For all mutations, we use the sampling probabilities calculated based on the availability of resistance prediction to INSTIs, NNRTIs, NRTIs, and PIs, $\pi_i^{s,INNP}$. Specifically, participant-visits in round s were assigned weights ($w_i^{s,INNP}$) according to:

$$w_i^{s,INNP} = \begin{cases} 0, & H_i = 0 \\ 0, & V_i = 0, H_i = 1 \\ 0, & T_i = 0, V_i = 1, H_i = 1 \\ 0, & R_i^{INNP} = 0, T_i = 1, V_i = 1, H_i = 1 \\ \left(\sum_{j \in s} \left(T_j V_j H_j\right)\right) \dfrac{\frac{1}{\pi_i^{s,INNP}}}{\sum_{j \in s} \left(T_j R_j^{INNP} \frac{1}{\pi_j^{s,INNP}}\right)}, & R_i^{INNP} = 1, T_i = 1, V_i = 1, H_i = 1 \end{cases}$$

Statistical Methods

Statistical analyses were conducted in R v.4.4.1.[13] Prevalence was estimated using Poisson regression with a log-link and robust (sandwich) standard errors[12] fit with general estimating equations using geepack v.1.3.11 to account for repeated measures and Emmeans v. 1.10.4. [14,15] Data analysis and visualization was done using tidyverse v.2.0.0,[16] ggplot2 v.3.5.1,[17] cowplot v.1.1.3,[18] patchwork v. 1.2.0,[19] and ggpattern v.1.1.1.[20] Readxl v.1.4.3[21] and haven v.2.5.4.9[22] were used to parse data files.

Supplementary Table 3: Summary of outcome measures

Measure	Model outcome (Numerator)	Population (Denominator)	Pseudo-population (IPW population)	Model predictors	Sub-group analyses	Corresponding figure
population prevalence of PLHIV in RCCS	HIV seropositive	all RCCS participants	all RCCS participants	survey round (2012 [REF], 2014, 2015, 2017, 2019)	..	Fig 1A
population prevalence of viraemic PLHIV in RCCS	viraemic and HIV seropositive	all RCCS participants	all RCCS participants	survey round (2014 [REF], 2015, 2017, 2019)	..	Fig 1A
population prevalence of viraemic pretreatment PLHIV in RCCS	pretreatment, viraemic, and HIV seropositive	all RCCS participants	all RCCS participants	survey round (2012 [REF], 2014, 2015, 2017, 2019)	..	Fig 1A
population prevalence of viraemic treatment-experienced PLHIV in RCCS	treatment-experienced, viraemic, and HIV seropositive	all RCCS participants	all RCCS participants	survey round (2014 [REF], 2015, 2017, 2019)	..	Fig 1A
population prevalence of viraemic NNRTI, NRTI, and PI resistance	viraemic NNRTI, NRTI, and PI resistance	viraemic PLHIV with resistance prediction to all NNRTIs, NRTIs, or PIs; non-viraemic PLHIV; participants not living with HIV	all RCCS participants	survey round (2015 [REF] and 2017)	Age category ([15,24] [REF], [25,34], and [35,49])<br>Community type (agrarian [REF], fishing, and trading)<br>Sex (female [REF], male)	Fig 1B
population prevalence of viraemic pretreatment NNRTI, NRTI, and PI resistance	viraemic pretreatment NNRTI, NRTI, and PI resistance	viraemic pretreatment PLHIV with resistance prediction to all NNRTIs, NRTIs, or PIs; non-viraemic pretreatment PLHIV; treatment-experienced PLHIV; participants not living with HIV	all RCCS participants	survey round (2012 [REF], 2014, 2015, 2017, 2019)	..	Fig 1C
population prevalence of viraemic treatment-experienced NNRTI, NRTI, and PI resistance	viraemic treatment-experienced NNRTI, NRTI, and PI resistance	viraemic treatment-experienced PLHIV with resistance prediction to all all NNRTIs, NRTIs, or PIs; non-viraemic treatment-experienced PLHIV; pretreatment PLHIV; participants not living with HIV	all RCCS participants	survey round (2015 [REF] and 2017)	..	Fig 1D
population prevalence of multi-class resistance	viraemic NNRTI, NRTI, PI, NNRTI/NRTI, NNRTI/PI, NRTI/PI, and NNRTI/NRTI/PI resistance	viraemic PLHIV with resistance prediction to all NNRTIs, NRTIs, and PIs; non-viraemic PLHIV; participants not living with HIV	all RCCS participants	survey round (2015 [REF] and 2017)	..	Fig 2A

prevalence of NNRTI, NRTI, and PI resistance among viraemic pretreatment PLHIV	viraemic NNRTI, NRTI, and PI resistance	viraemic pretreatment PLHIV with resistance prediction to all NNRTIs, NRTIs, or PIs.	viraemic pretreatmen t PLHIV	survey round (2012 [REF], 2014, 2015, 2017, 2019)	Age category ([15,24] [REF], [25,34], and [35,49]) Community type (agrarian [REF], fishing, and trading) Sequencing technology (amplicon v. veSeq-HIV) Sex (female [REF], male)	Fig 3A
prevalence of resistance mutations among viraemic pretreatment PLHIV	mutation presence	viraemic pretreatment PLHIV with resistance prediction to all INSTIs, NNRTIs, NRTIs, and PIs.	viraemic pretreatmen t PLHIV	survey round (2012 [REF], 2014, 2015, 2017, 2019)	..	Fig 3B
prevalence of NNRTI, NRTI, and PI resistance among viraemic treatment-experienced PLHIV	viraemic NNRTI, NRTI, and PI resistance	viraemic treatment-experienced PLHIV with resistance prediction to all NNRTI, NRTI, or PIs.	viraemic treatment-experienced PLHIV	survey round (2015 [REF] and 2017)	Age category ([15,24] [REF], [25,34], and [35,49]) Community type (agrarian [REF], fishing, and trading) Sex (female [REF], male)	Fig 4A
prevalence of resistance mutations among viraemic treatment-experienced PLHIV	mutation presence	viraemic treatment-experienced PLHIV with resistance prediction to all INSTIs, NNRTIs, NRTIs, and PIs.	viraemic treatment-experienced PLHIV	survey round (2015 [REF] and 2017)	..	Fig 4B

Measure refers to how a given analysis is referred to in text, figures, and tables. The model outcome is the participant-visit level characteristic which is being modeled in each analysis. The population is the data among which a given outcome is modeled. Pseudo-population is the population which is being approximated using IPW. Model predictors are the variables included in each model. Interaction included with model predictors in bivariate analyses. PLHIV = people living with HIV. NNRTI = non-nucleoside reverse transcriptase inhibitors. NRTI = nucleoside reverse transcriptase inhibitors. PI = protease inhibitors. INSTI = integrase strand transfer inhibitors. IPW = inverse probability weighting.

Supplementary Table 4: Drugs to which resistance was predicted

Generic name	Abbreviation	Class
bictegravir	BIC	INSTI
cabotegravir	CAB	INSTI
dolutegravir	DTG	INSTI
elvitegravir	EVG	INSTI
raltegravir	RAL	INSTI
doravirine	DOR	NNRTI
efavirenz	EFV	NNRTI
Etravirine	ETR	NNRTI
nevirapine	NVP	NNRTI
rilpivirine	RPV	NNRTI
abacavir	ABC	NRTI
didanosine	DDI	NRTI
emtricitabine	FTC	NRTI
lamivudine	3TC	NRTI
stavudine	D4T	NRTI
tenofovir	TDF	NRTI
zidovudine	AZT	NRTI
atazanavir	ATV	PI
darunavir	DRV	PI
fosamprenavir	FPV	PI
indinavir	IDV	PI
lopinavir	LPV	PI
nelfinavir	NFV	PI
saquinavir	SQV	PI
tipranavir	TPV	PI

INSTI = integrase strand transfer inhibitor. NNRTI = non-nucleoside reverse transcriptase inhibitor. NRTI = nucleoside reverse transcriptase inhibitor. PI = protease inhibitor.

Supplementary Table 5: Sampling probability model for the probability that genotyping data is available among all viraemic PLHIV

		NNRTI		NRTI		PI		NNRTI/NRTI/PI		INSTI/NRTI/PI	
Survey round	Variable	Coeff. (95% CI)	*p*-value	Coeff. (95% CI)	*p*-value	Coeff. (95% CI)	*p*-value	Coeff. (95% CI)	*p*-value	Coeff. (95% CI)	*p*-value
2012	(Intercept)	0.04 (0.02, 0.06)	<0.0001	0.03 (0.02, 0.05)	<0.0001	0.13 (0.09, 0.17)	<0.0001	0.03 (0.02, 0.04)	<0.0001	0 (0, 0.01)	<0.0001
	Age category										
	[15,24]	ref	ref	ref	ref	ref	ref	ref	ref	ref	ref
	[25,34]	1 (0.89, 1.12)	1	1.01 (0.91, 1.13)	0.83	1.02 (0.94, 1.1)	0.65	0.99 (0.88, 1.12)	0.89	0.94 (0.77, 1.15)	0.55
	[35,49]	0.98 (0.86, 1.11)	0.74	1 (0.89, 1.14)	0.94	1.03 (0.94, 1.12)	0.54	0.97 (0.85, 1.11)	0.68	0.92 (0.74, 1.15)	0.47
	Community type										
	Agrarian	ref	ref	ref	ref	ref	ref	ref	ref	ref	ref
	Fishing	0.85 (0.72, 0.99)	0.038	0.9 (0.77, 1.06)	0.2	1.07 (0.95, 1.2)	0.24	0.85 (0.72, 1)	0.056	0.91 (0.66, 1.24)	0.54
	Trading	0.96 (0.85, 1.09)	0.5	0.97 (0.86, 1.1)	0.66	1 (0.92, 1.1)	0.93	0.96 (0.84, 1.09)	0.54	1.01 (0.81, 1.25)	0.94
	Sex										
	F	ref	ref	ref	ref	ref	ref	ref	ref	ref	ref
	M	1.05 (0.96, 1.15)	0.25	1.06 (0.98, 1.16)	0.16	1.04 (0.98, 1.11)	0.17	1.07 (0.97, 1.17)	0.17	1.07 (0.92, 1.25)	0.38
	Missing VL	14.74 (9.73, 22.35)	<0.0001	16.35 (10.91, 24.49)	<0.0001	5.23 (3.93, 6.96)	<0.0001	18.49 (12.03, 28.43)	<0.0001	125.82 (60.52, 261.57)	<0.0001
	Log_{10} VL	1.88 (1.72, 2.04)	<0.0001	1.91 (1.76, 2.07)	<0.0001	1.46 (1.38, 1.55)	<0.0001	1.97 (1.8, 2.15)	<0.0001	2.89 (2.51, 3.34)	<0.0001
2014	(Intercept)	0.02 (0.01, 0.04)	<0.0001	0.02 (0.01, 0.03)	<0.0001	0.05 (0.03, 0.08)	<0.0001	0.02 (0.01, 0.03)	<0.0001	0 (0, 0.01)	<0.0001
	Age category										
	[15,24]	ref	ref	ref	ref	ref	ref	ref	ref	ref	ref
	[25,34]	0.74 (0.62, 0.89)	0.0013	0.76 (0.63, 0.92)	0.0039	0.75 (0.64, 0.87)	3e-04	0.75 (0.62, 0.91)	0.0034	0.76 (0.58, 0.98)	0.032
	[35,49]	0.67 (0.55, 0.83)	0.0002	0.73 (0.59, 0.9)	0.0028	0.7 (0.58, 0.83)	<0.0001	0.7 (0.57, 0.87)	0.001	0.73 (0.55, 0.97)	0.028
	Community type										
	Agrarian	ref	ref	ref	ref	ref	ref	ref	ref	ref	ref
	Fishing	0.69 (0.58, 0.82)	<0.0001	0.77 (0.65, 0.92)	0.0033	0.74 (0.64, 0.87)	0.00019	0.75 (0.63, 0.9)	0.0019	0.54 (0.42, 0.68)	<0.0001
	Trading	0.99 (0.82, 1.2)	0.91	1.06 (0.87, 1.29)	0.56	1.11 (0.94, 1.32)	0.22	1.06 (0.87, 1.3)	0.55	0.99 (0.77, 1.26)	0.92
	Sex										
	F	ref	ref	ref	ref	ref	ref	ref	ref	ref	ref
	M	0.85 (0.72, 0.99)	0.037	0.86 (0.73, 1.01)	0.058	0.9 (0.78, 1.03)	0.14	0.86 (0.73, 1.01)	0.071	0.87 (0.7, 1.07)	0.18
	Log_{10} VL	2.11 (1.9, 2.35)	<0.0001	2.14 (1.93, 2.39)	<0.0001	1.78 (1.61, 1.96)	<0.0001	2.2 (1.97, 2.45)	<0.0001	2.81 (2.46, 3.22)	<0.0001
2015	(Intercept)	0.42 (0.35, 0.52)	<0.0001	0.29 (0.23, 0.36)	<0.0001	0.4 (0.32, 0.49)	<0.0001	0.24 (0.19, 0.31)	<0.0001	0.24 (0.18, 0.3)	<0.0001
	Age category										
	[15,24]	ref	ref	ref	ref	ref	ref	ref	ref	ref	ref
	[25,34]	0.97 (0.9, 1.04)	0.32	0.91 (0.84, 0.98)	0.019	0.95 (0.89, 1.02)	0.13	0.93 (0.85, 1.01)	0.096	0.94 (0.86, 1.03)	0.18
	[35,49]	0.97 (0.89, 1.04)	0.38	0.95 (0.87, 1.03)	0.21	0.98 (0.91, 1.06)	0.65	0.97 (0.89, 1.07)	0.58	0.98 (0.9, 1.08)	0.72

	Community type										
	Agrarian	ref	ref	ref	ref	ref	ref	ref	ref	ref	ref
	Fishing	1.03 (0.96, 1.09)	0.42	1.01 (0.94, 1.09)	0.74	1.01 (0.95, 1.08)	0.65	1 (0.93, 1.07)	0.93	1 (0.93, 1.07)	0.92
	Trading	1.01 (0.94, 1.09)	0.76	1.06 (0.97, 1.15)	0.18	1.04 (0.96, 1.12)	0.35	1.05 (0.96, 1.14)	0.32	1.03 (0.94, 1.13)	0.55
	Sex										
	F	ref	ref	ref	ref	ref	ref	ref	ref	ref	ref
	M	0.97 (0.91, 1.02)	0.23	0.97 (0.91, 1.04)	0.38	0.94 (0.89, 1)	0.041	0.96 (0.9, 1.03)	0.22	0.96 (0.89, 1.02)	0.2
	Treatment										
	Treatment-experienced	ref	ref	ref	ref	ref	ref	ref	ref	ref	ref
	Pretreatment	1.07 (1, 1.16)	0.061	1.05 (0.97, 1.14)	0.2	1.1 (1.02, 1.19)	0.014	1.1 (1.01, 1.2)	0.036	1.1 (1.01, 1.21)	0.032
	Log_{10} VL	1.17 (1.12, 1.22)	<0.0001	1.28 (1.22, 1.34)	<0.0001	1.19 (1.14, 1.24)	<0.0001	1.31 (1.25, 1.38)	<0.0001	1.32 (1.25, 1.39)	<0.0001
2017	(Intercept)	0.76 (0.62, 0.92)	0.0049	0.64 (0.51, 0.79)	<0.0001	0.76 (0.62, 0.92)	0.0048	0.63 (0.51, 0.79)	<0.0001	0.63 (0.51, 0.79)	<0.0001
	Age category										
	[15,24]	ref	ref	ref	ref	ref	ref	ref	ref	ref	ref
	[25,34]	0.98 (0.91, 1.05)	0.5	0.94 (0.88, 1.02)	0.14	0.96 (0.9, 1.04)	0.31	0.95 (0.88, 1.02)	0.15	0.95 (0.88, 1.02)	0.15
	[35,49]	0.93 (0.86, 1.02)	0.12	0.9 (0.82, 0.98)	0.021	0.93 (0.86, 1.01)	0.1	0.9 (0.82, 0.98)	0.018	0.9 (0.82, 0.98)	0.018
	Community type										
	Agrarian	ref	ref	ref	ref	ref	ref	ref	ref	ref	ref
	Fishing	1.03 (0.96, 1.11)	0.44	1.05 (0.97, 1.14)	0.2	1.04 (0.97, 1.12)	0.3	1.05 (0.97, 1.14)	0.19	1.05 (0.97, 1.14)	0.19
	Trading	1.03 (0.94, 1.11)	0.55	1.04 (0.95, 1.13)	0.42	1.04 (0.96, 1.13)	0.36	1.03 (0.94, 1.13)	0.53	1.03 (0.94, 1.13)	0.53
	Sex										
	F	ref	ref	ref	ref	ref	ref	ref	ref	ref	ref
	M	0.97 (0.92, 1.04)	0.42	0.97 (0.91, 1.04)	0.36	0.97 (0.91, 1.03)	0.37	0.97 (0.9, 1.03)	0.3	0.97 (0.9, 1.03)	0.3
	Treatment										
	Treatment-experienced	ref	ref	ref	ref	ref	ref	ref	ref	ref	ref
	Pretreatment	1.07 (0.99, 1.15)	0.089	1.04 (0.97, 1.13)	0.26	1.06 (0.99, 1.14)	0.12	1.05 (0.97, 1.14)	0.2	1.05 (0.97, 1.14)	0.2
	Log10 VL	1.02 (0.98, 1.07)	0.26	1.07 (1.02, 1.12)	0.0053	1.03 (0.98, 1.07)	0.24	1.07 (1.02, 1.12)	0.0052	1.07 (1.02, 1.12)	0.0052
2019	(Intercept)	1.21 (0.48, 3.06)	0.69	1.04 (0.4, 2.65)	0.94	1.52 (0.61, 3.75)	0.37	1.04 (0.4, 2.65)	0.94	1.04 (0.4, 2.65)	0.94
	Age category										
	[15,24]	ref	ref	ref	ref	ref	ref	ref	ref	ref	ref
	[25,34]	0.94 (0.67, 1.34)	0.75	0.98 (0.69, 1.41)	0.92	0.93 (0.66, 1.29)	0.65	0.98 (0.69, 1.41)	0.92	0.98 (0.69, 1.41)	0.92
	[35,49]	0.96 (0.66, 1.4)	0.83	0.97 (0.66, 1.44)	0.9	0.94 (0.66, 1.36)	0.76	0.97 (0.66, 1.44)	0.9	0.97 (0.66, 1.44)	0.9
	Community type										
	Agrarian	ref	ref	ref	ref	ref	ref	ref	ref	ref	ref
	Fishing	1.3 (0.95, 1.77)	0.1	1.25 (0.91, 1.72)	0.18	1.37 (1.01, 1.86)	0.042	1.25 (0.91, 1.72)	0.18	1.25 (0.91, 1.72)	0.18

Trading	0.92 (0.64, 1.33)	0.67	0.93 (0.65, 1.34)	0.7	0.92 (0.64, 1.32)	0.64	0.93 (0.65, 1.34)	0.7	0.93 (0.65, 1.34)	0.7
Sex										
F	ref	ref	ref	ref	ref	ref	ref	ref	ref	ref
M	0.89 (0.68, 1.18)	0.42	0.89 (0.67, 1.18)	0.42	0.91 (0.69, 1.19)	0.49	0.89 (0.67, 1.18)	0.42	0.89 (0.67, 1.18)	0.42
Log10 VL	0.78 (0.64, 0.96)	0.017	0.81 (0.66, 0.99)	0.038	0.75 (0.61, 0.91)	0.0035	0.81 (0.66, 0.99)	0.038	0.81 (0.66, 0.99)	0.038

Estimates were generated using robust log-Poisson regression specific to each survey round (row sections) and outcome measure (columns). 2012, 2014, and 2019 survey rounds include only pretreatment PLHIV. 95% confidence intervals indicate the Wald confidence interval around the mean value in each category. *p*-values that coefficients are different from 0 at the $\alpha = 0.05$ level were calculated using the Wald method. PLHIV = people living with HIV. NNRTI = non-nucleoside reverse transcriptase inhibitors. NRTI = nucleoside reverse transcriptase inhibitors. PI = protease inhibitors.

Supplementary Figure 2: Supplementary Figure 2: Number of repeat visits among RCCS participants stratified by viremia and treatment-status

Viral load data was not routinely collected for treatment-experienced participants in the 2012 survey round and therefore the “viraemic” and “viraemic treatment-experienced” panels include only data from the 2014 survey round and later.

Supplementary Table 6: Demographics of study participants by survey round

	Survey round				
	**2012**	**2014**	**2015**	**2017**	**2019**
Overall	17167	17992	19336	19803	19324
Age (median [Q1-Q3])	28 [22-35]	29 [21-36]	29 [22-36]	29 [21-37]	29 [21-38]
Age Category					
[15,24]	6026 (35.1%)	6401 (35.58%)	6818 (35.26%)	6974 (35.22%)	6691 (34.63%)
[25,34]	6429 (37.45%)	6291 (34.97%)	6414 (33.17%)	6370 (32.17%)	5929 (30.68%)
[35,49]	4712 (27.45%)	5300 (29.46%)	6104 (31.57%)	6459 (32.62%)	6704 (34.69%)
Community type					
Agrarian	7877 (45.88%)	8197 (45.56%)	8808 (45.55%)	8747 (44.17%)	8848 (45.79%)
Fishing	3876 (22.58%)	3930 (21.84%)	4238 (21.92%)	4756 (24.02%)	4081 (21.12%)
Trading	5414 (31.54%)	5865 (32.6%)	6290 (32.53%)	6300 (31.81%)	6395 (33.09%)
Sex					
F	9240 (53.82%)	9672 (53.76%)	10452 (54.05%)	10552 (53.28%)	10448 (54.07%)
M	7927 (46.18%)	8320 (46.24%)	8884 (45.95%)	9251 (46.72%)	8876 (45.93%)

Percentages represent the proportion of total participant-visits in each survey round belonging to each sub-category.

Supplementary Table 7: Demographics of participants living with HIV by survey round

	Survey round				
	2012	2014	2015	2017	2019
Overall	3498	3388	3615	3636	3323
Age (median [Q1-Q3])	32 [27-37]	33 [27-39]	34 [28-39]	34 [29-40]	36 [30-42]
Age Category					
[15,24]	531 (15.18%)	478 (14.11%)	453 (12.53%)	372 (10.23%)	264 (7.94%)
[25,34]	1663 (47.54%)	1469 (43.36%)	1481 (40.97%)	1453 (39.96%)	1205 (36.26%)
[35,49]	1304 (37.28%)	1441 (42.53%)	1681 (46.5%)	1811 (49.81%)	1854 (55.79%)
Community type					
Agrarian	1091 (31.19%)	1081 (31.91%)	1141 (31.56%)	1072 (29.48%)	1067 (32.11%)
Fishing	1599 (45.71%)	1519 (44.83%)	1597 (44.18%)	1736 (47.74%)	1500 (45.14%)
Trading	808 (23.1%)	788 (23.26%)	877 (24.26%)	828 (22.77%)	756 (22.75%)
Sex					
F	2169 (62.01%)	2103 (62.07%)	2297 (63.54%)	2242 (61.66%)	2125 (63.95%)
M	1329 (37.99%)	1285 (37.93%)	1318 (36.46%)	1394 (38.34%)	1198 (36.05%)

Percentages represent the proportion of total participant-visits in each survey round belonging to each sub-category.

Supplementary Table 8: Prevalence of PLHIV, viraemic PLHIV, viraemic pretreatment PLHIV, and viraemic treatment-experienced PLHIV by survey round

	PLHIV					Viraemic PLHIV			
Survey round	*n*	Obs (%)	Prev. % (95% CI)	Prev. ratio (95% CI)	*p*-value	Obs (%)	Prev. % (95% CI)	Prev. ratio (95% CI)	*p*-value
2012	17167	3498 (20.38%)	20.38 (19.78, 20.99)	ref	ref				
2014	17992	3388 (18.83%)	18.83 (18.27, 19.41)	0.92 (0.9, 0.95)	<0.0001	1464 (8.14%)	8.14 (7.75, 8.55)	ref	ref
2015	19336	3615 (18.7%)	18.7 (18.15, 19.25)	0.92 (0.89, 0.95)	<0.0001	1026 (5.31%)	5.31 (5, 5.63)	0.65 (0.61, 0.69)	<0.0001
2017	19803	3636 (18.36%)	18.36 (17.83, 18.91)	0.9 (0.87, 0.93)	<0.0001	728 (3.68%)	3.68 (3.42, 3.95)	0.45 (0.42, 0.49)	<0.0001
2019	19324	3323 (17.2%)	17.2 (16.67, 17.74)	0.84 (0.81, 0.87)	<0.0001	453 (2.34%)	2.34 (2.14, 2.57)	0.29 (0.26, 0.32)	<0.0001

	Viraemic pretreatment PLHIV					Viraemic treatment-experienced PLHIV			
Survey round	*n*	Obs (%)	Prev. % (95% CI)	Prev. ratio (95% CI)	*p*-value	Obs (%)	Prev. % (95% CI)	Prev. ratio (95% CI)	*p*-value
2012	17167	1985 (11.56%)	11.56 (11.09, 12.05)	ref	ref				
2014	17992	1318 (7.33%)	7.33 (6.95, 7.72)	0.63 (0.6, 0.67)	<0.0001	146 (0.81%)	0.81 (0.69, 0.95)	ref	ref
2015	19336	822 (4.25%)	4.25 (3.98, 4.55)	0.37 (0.34, 0.4)	<0.0001	204 (1.06%)	1.06 (0.92, 1.21)	1.3 (1.09, 1.56)	0.0041
2017	19803	502 (2.53%)	2.53 (2.33, 2.76)	0.22 (0.2, 0.24)	<0.0001	226 (1.14%)	1.14 (1, 1.3)	1.41 (1.16, 1.71)	0.00058
2019	19324	288 (1.49%)	1.49 (1.33, 1.67)	0.13 (0.11, 0.15)	<0.0001	165 (0.85%)	0.85 (0.73, 0.99)	1.05 (0.85, 1.31)	0.64

Estimates were generated using log-Poisson regression with robust standard errors with survey round as a predictor variable. Generalized estimating equations with correlation structure selection by Quasi Information Criterion for each outcome (all independent). Confidence intervals indicate the Wald confidence interval around the mean value in each category. p-values that coefficients are different from 0 at the $\alpha = 0.05$ level were calculated using the Wald method. PLHIV = people living with HIV.

Supplementary Table 9: Number of participant-visits contributed by PLHIV with history of treatment

	PLHIV	
	**Participant-visits**	**Treatment-experienced**
Overall	17460	10745 (61.54%)
Survey round		
2012	3498	876 (25.04%)
2014	3388	1674 (49.41%)
2015	3615	2451 (67.8%)
2017	3636	2911 (80.06%)
2019	3323	2833 (85.25%)

Percentages represent the proportion of total participant-visits contributed by PLHIV in each survey round with self-reported history of antiretroviral therapy.

Supplementary Table 10: Number of viraemic participant-visits from which deep-sequence based identification of drug resistance mutations was attempted

	Viraemic PLWHIV			Viraemic pretreatment PLWHIV			Viraemic treatment-experienced PLWHIV		
	Participant-visits	Deep-sequence based DRM attempted (%)	*p*-value	Participant-visits	Deep-sequence based DRM attempted (%)	*p*-value	Participant-visits	Deep-sequence based DRM attempted (%)	*p*-value
Overall	5691	4523 (79.48%)		4915	4094 (83.3%)		776	429 (55.28%)	
Age Category			0.019			0.076			0.82
[15,24]	1111	950 (85.51%)		997	884 (88.67%)		114	66 (57.89%)	
[25,34]	2811	2235 (79.51%)		2453	2034 (82.92%)		358	201 (56.15%)	
[35,49]	1769	1338 (75.64%)		1465	1176 (80.27%)		304	162 (53.29%)	
Community type			0.55			0.26			0.069
Agrarian	1621	1287 (79.4%)		1417	1190 (83.98%)		204	97 (47.55%)	
Fishing	2860	2246 (78.53%)		2454	1998 (81.42%)		406	248 (61.08%)	
Trading	1210	990 (81.82%)		1044	906 (86.78%)		166	84 (50.6%)	
Sex			0.14			0.055			0.77
F	3008	2440 (81.12%)		2564	2198 (85.73%)		444	242 (54.5%)	
M	2683	2083 (77.64%)		2351	1896 (80.65%)		332	187 (56.33%)	
Survey round			0.0005			0.0005			0.0005
2012	2020	1971 (97.57%)		1985	1969 (99.19%)		35	2 (5.71%)	
2014	1464	798 (54.51%)		1318	794 (60.24%)		146	4 (2.74%)	
2015	1026	948 (92.4%)		822	764 (92.94%)		204	184 (90.2%)	
2017	728	635 (87.23%)		502	445 (88.65%)		226	190 (84.07%)	
2019	453	171 (37.75%)		288	122 (42.36%)		165	49 (29.7%)	
Viral load (log10 copies/mL)			0.0005			0.0005			0.76
(3,4]	2100	1647 (78.43%)		1747	1445 (82.71%)		353	202 (57.22%)	
(4,5]	2231	1696 (76.02%)		1898	1515 (79.82%)		333	181 (54.35%)	
(5,Inf]	598	428 (71.57%)		508	382 (75.2%)		90	46 (51.11%)	
missing	762	752 (98.69%)		762	752 (98.69%)				

Percentages show the percentage of all participant-visits within a category (columns) that have successful genotyping for at least one drug. *P*-values calculated using χ^2 goodness-of-fit test with Monte Carlo simulation. Viraemic PLHIV is a sum of viraemic pretreatment PLHIV and viraemic treatment-experienced PLHIV. Viral load measurements were available for only a subset of PLHIV in the 2012 survey roads. Missing viral load measurements for pretreatment PLHIV in the 2012 survey round were imputed. All other participant-visits with missing viral load measurements were dropped. PLHIV = people living with HIV.

Supplementary Table 11: Sequencing approach used to generate sequence data by survey round

	Survey round				
	2012	2014	2015	2017	2019
Overall	2005	799	948	636	171
Sequencing approach					
amplicon	1867 (94.72%)	607 (76.07%)	6 (0.63%)		
veSeq-HIV	104 (5.28%)	191 (23.93%)	942 (99.37%)	635 (100%)	171 (100%)

Supplementary Table 12: Number of viraemic participant-visits with attempted deep-sequence based identification of drug resistance mutations that had successful genotyping for at least one drug

	Viraemic PLWHIV			Viraemic pretreatment PLWHIV			Viraemic treatment-experienced PLWHIV		
	**Participant-visits**	**Genotype data for ≥1 drug (%)**	***p*-value**	**Participant-visits**	**Genotype data for ≥1 drug (%)**	***p*-value**	**Participant-visits**	**Genotype data for ≥1 drug (%)**	***p*-value**
Overall	4523	4072 (90.03%)		4094	3655 (89.28%)		429	417 (97.2%)	
Age Category			0.83			0.84			0.98
[15,24]	950	840 (88.42%)		884	775 (87.67%)		66	65 (98.48%)	
[25,34]	2235	2020 (90.38%)		2034	1824 (89.68%)		201	196 (97.51%)	
[35,49]	1338	1212 (90.58%)		1176	1056 (89.8%)		162	156 (96.3%)	
Community type			0.83			0.84			0.98
Agrarian	1287	1172 (91.06%)		1190	1077 (90.5%)		97	95 (97.94%)	
Fishing	2246	2023 (90.07%)		1998	1781 (89.14%)		248	242 (97.58%)	
Trading	990	877 (88.59%)		906	797 (87.97%)		84	80 (95.24%)	
Sex			0.37			0.31			0.88
F	2440	2168 (88.85%)		2198	1931 (87.85%)		242	237 (97.93%)	
M	2083	1904 (91.41%)		1896	1724 (90.93%)		187	180 (96.26%)	
Survey round			0.0015			0.0005			0.97
2012	1971	1694 (85.95%)		1969	1693 (85.98%)		2	1 (50%)	
2014	798	657 (82.33%)		794	653 (82.24%)		4	4 (100%)	
2015	948	921 (97.15%)		764	744 (97.38%)		184	177 (96.2%)	
2017	635	630 (99.21%)		445	443 (99.55%)		190	187 (98.42%)	
2019	171	170 (99.42%)		122	122 (100%)		49	48 (97.96%)	
Viral load (log10 copies/mL)			0.0005			0.0005			0.91
(3,4]	1647	1359 (82.51%)		1445	1167 (80.76%)		202	192 (95.05%)	
(4,5]	1696	1630 (96.11%)		1515	1451 (95.78%)		181	179 (98.9%)	
(5,Inf]	428	421 (98.36%)		382	375 (98.17%)		46	46 (100%)	
missing	752	662 (88.03%)		752	662 (88.03%)				
Sequencing approach			0.0005			0.0005			0.83
amplicon	2480	2106 (84.92%)		2474	2101 (84.92%)		6	5 (83.33%)	
veSEQ-HIV	2043	1966 (96.23%)		1620	1554 (95.93%)		423	412 (97.4%)	

Excludes participant-visits on which deep-sequence based identification of drug resistance mutations was not attempted. Percentages show the percentage of all participant-visits within a category (columns) that have successful genotyping for at least one drug. P-values calculated using χ^2 goodness-of-fit test with Monte Carlo simulation. Viraemic PLHIV is a sum of viraemic pretreatment PLHIV and viraemic treatment-experienced PLHIV. Viral load measurements were available for only a subset of PLHIV in the 2012 survey roads. Missing viral load measurements for pretreatment PLHIV in the 2012 survey round were imputed. All other participant-visits with missing viral load measurements were dropped. PLHIV = people living with HIV.

Supplementary Table 13: Deep-sequencing quality summary statistics among participant-visits with successful genotyping for at least one drug

	Quantile				
	**0.025**	**0.25**	**0.5**	**0.75**	**0.975**
HIV reads	2025	32681	97973	236689	2210972.5
Mapped reads	316	4893	17912	49666	168641
Insert size (quantile)					
0.05	81	125	134	153	178
0.5	185.5	240	267	366	419
0.95	467.5	517	567	949	1104
Duplicate reads (%)	32.42	57.67	74.65	91.07	95.75
Length	1287	3544	7215	8938	9040

Supplementary Table 14: Number of viraemic participant-visits with attempted deep-sequence based identification of drug resistance mutations that had successful genotyping for all INSTIs

	Viraemic PLWHIV			Viraemic pretreatment PLWHIV			Viraemic treatment-experienced PLWHIV		
	**Participant-visits**	**Genotype data for all INSTIs (%)**	***p*-value**	**Participant-visits**	**Genotype data for all INSTIs (%)**	***p*-value**	**Participant-visits**	**Genotype data for all INSTIs (%)**	***p*-value**
Overall	4523	2578 (57%)		4094	2168 (52.96%)		429	410 (95.57%)	
Age Category			0.21			0.41			0.96
[15,24]	950	527 (55.47%)		884	463 (52.38%)		66	64 (96.97%)	
[25,34]	2235	1248 (55.84%)		2034	1054 (51.82%)		201	194 (96.52%)	
[35,49]	1338	803 (60.01%)		1176	651 (55.36%)		162	152 (93.83%)	
Community type			0.008			0.0005			0.86
Agrarian	1287	794 (61.69%)		1190	700 (58.82%)		97	94 (96.91%)	
Fishing	2246	1205 (53.65%)		1998	965 (48.3%)		248	240 (96.77%)	
Trading	990	579 (58.48%)		906	503 (55.52%)		84	76 (90.48%)	
Sex			0.01			0.0025			0.89
F	2440	1326 (54.34%)		2198	1093 (49.73%)		242	233 (96.28%)	
M	2083	1252 (60.11%)		1896	1075 (56.7%)		187	177 (94.65%)	
Survey round			0.0005			0.0005			0.56
2012	1971	537 (27.25%)		1969	537 (27.27%)		2	0 (0%)	
2014	798	335 (41.98%)		794	333 (41.94%)		4	2 (50%)	
2015	948	909 (95.89%)		764	735 (96.2%)		184	174 (94.57%)	
2017	635	627 (98.74%)		445	441 (99.1%)		190	186 (97.89%)	
2019	171	170 (99.42%)		122	122 (100%)		49	48 (97.96%)	
Viral load (log10 copies/mL)			0.0005			0.0005			0.86
(3,4]	1647	816 (49.54%)		1445	628 (43.46%)		202	188 (93.07%)	
(4,5]	1696	1152 (67.92%)		1515	976 (64.42%)		181	176 (97.24%)	
(5,Inf]	428	362 (84.58%)		382	316 (82.72%)		46	46 (100%)	
missing	752	248 (32.98%)		752	248 (32.98%)				
Sequencing approach			0.0005			0.0005			0.3
amplicon	2480	629 (25.36%)		2474	626 (25.3%)		6	3 (50%)	
veSEQ-HIV	2043	1949 (95.4%)		1620	1542 (95.19%)		423	407 (96.22%)	

Excludes participant-visits on which deep-sequence based identification of drug resistance mutations was not attempted. Percentages show the percentage of all participant-visits within a category (columns) that have successful genotyping for all INSTIs. *P*-values calculated using χ^2 goodness-of-fit test with Monte Carlo simulation. Viraemic PLHIV is a sum of viraemic pretreatment PLHIV and viraemic treatment-experienced PLHIV. Viral load measurements were available for only a subset of PLHIV in the 2012 survey roads. Missing viral load measurements for pretreatment PLHIV in the 2012 survey round were imputed. All other participant-visits with missing viral load measurements were dropped. PLHIV = people living with HIV. DRM = drug resistance mutations. INSTI = integrase strand transfer inhibitor.

Supplementary Table 15: Number of viraemic participant-visits with attempted deep-sequence based identification of drug resistance mutations that had successful genotyping for all NNRTIs

	Viraemic PLWHIV			Viraemic pretreatment PLWHIV			Viraemic treatment-experienced PLWHIV		
	Participant-visits	Genotype data for all NNRTIs (%)	*p*-value	Participant-visits	Genotype data for all NNRTIs (%)	*p*-value	Participant-visits	Genotype data for all NNRTIs (%)	*p*-value
Overall	4523	3050 (67.43%)		4094	2656 (64.88%)		429	394 (91.84%)	
Age Category			0.78			0.89			0.94
[15,24]	950	629 (66.21%)		884	566 (64.03%)		66	63 (95.45%)	
[25,34]	2235	1502 (67.2%)		2034	1317 (64.75%)		201	185 (92.04%)	
[35,49]	1338	919 (68.68%)		1176	773 (65.73%)		162	146 (90.12%)	
Community type			0.22			0.088			0.96
Agrarian	1287	912 (70.86%)		1190	822 (69.08%)		97	90 (92.78%)	
Fishing	2246	1481 (65.94%)		1998	1252 (62.66%)		248	229 (92.34%)	
Trading	990	657 (66.36%)		906	582 (64.24%)		84	75 (89.29%)	
Sex			0.009			0.005			1
F	2440	1574 (64.51%)		2198	1352 (61.51%)		242	222 (91.74%)	
M	2083	1476 (70.86%)		1896	1304 (68.78%)		187	172 (91.98%)	
Survey round			0.0005			0.0005			0.84
2012	1971	972 (49.32%)		1969	971 (49.31%)		2	1 (50%)	
2014	798	425 (53.26%)		794	422 (53.15%)		4	3 (75%)	
2015	948	865 (91.24%)		764	705 (92.28%)		184	160 (86.96%)	
2017	635	623 (98.11%)		445	439 (98.65%)		190	184 (96.84%)	
2019	171	165 (96.49%)		122	119 (97.54%)		49	46 (93.88%)	
Viral load (log10 copies/mL)			0.0005			0.0005			0.41
(3,4]	1647	895 (54.34%)		1445	723 (50.03%)		202	172 (85.15%)	
(4,5]	1696	1356 (79.95%)		1515	1179 (77.82%)		181	177 (97.79%)	
(5,Inf]	428	393 (91.82%)		382	348 (91.1%)		46	45 (97.83%)	
missing	752	406 (53.99%)		752	406 (53.99%)				
Sequencing approach			0.0005			0.0005			0.56
amplicon	2480	1215 (48.99%)		2474	1211 (48.95%)		6	4 (66.67%)	
veSEQ-HIV	2043	1835 (89.82%)		1620	1445 (89.2%)		423	390 (92.2%)	

Excludes participant-visits on which deep-sequence based identification of drug resistance mutations was not attempted. Percentages show the percentage of all participant-visits within a category (columns) that have successful genotyping for all NNRTIs. *P*-values calculated using χ^2 goodness-of-fit test with Monte Carlo simulation. Viraemic PLHIV is a sum of viraemic pretreatment PLHIV and viraemic treatment-experienced PLHIV. Viral load measurements were available for only a subset of PLHIV in the 2012 survey roads. Missing viral load measurements for pretreatment PLHIV in the 2012 survey round were imputed. All other participant-visits with missing viral load measurements were dropped. PLHIV = people living with HIV. NNRTI = non-nucleoside reverse transcriptase inhibitor.

Supplementary Table 16: Number of viraemic participant-visits with attempted deep-sequence based identification of drug resistance mutations that had successful genotyping for all NRTIs

	Viraemic PLWHIV			Viraemic pretreatment PLWHIV			Viraemic treatment-experienced PLWHIV		
	Participant-visits	Genotype data for all NRTIs (%)	*p*-value	Participant-visits	Genotype data for all NRTIs (%)	*p*-value	Participant-visits	Genotype data for all NRTIs (%)	*p*-value
Overall	4523	3009 (66.53%)		4094	2626 (64.14%)		429	383 (89.28%)	
Age Category			0.55			0.71			0.96
[15,24]	950	620 (65.26%)		884	559 (63.24%)		66	61 (92.42%)	
[25,34]	2235	1472 (65.86%)		2034	1294 (63.62%)		201	178 (88.56%)	
[35,49]	1338	917 (68.54%)		1176	773 (65.73%)		162	144 (88.89%)	
Community type			0.53			0.39			0.94
Agrarian	1287	884 (68.69%)		1190	795 (66.81%)		97	89 (91.75%)	
Fishing	2246	1473 (65.58%)		1998	1254 (62.76%)		248	219 (88.31%)	
Trading	990	652 (65.86%)		906	577 (63.69%)		84	75 (89.29%)	
Sex			0.004			0.001			1
F	2440	1543 (63.24%)		2198	1327 (60.37%)		242	216 (89.26%)	
M	2083	1466 (70.38%)		1896	1299 (68.51%)		187	167 (89.3%)	
Survey round			0.0005			0.0005			0.71
2012	1971	998 (50.63%)		1969	997 (50.63%)		2	1 (50%)	
2014	798	420 (52.63%)		794	417 (52.52%)		4	3 (75%)	
2015	948	817 (86.18%)		764	665 (87.04%)		184	152 (82.61%)	
2017	635	612 (96.38%)		445	430 (96.63%)		190	182 (95.79%)	
2019	171	162 (94.74%)		122	117 (95.9%)		49	45 (91.84%)	
Viral load (log10 copies/mL)			0.0005			0.0005			0.097
(3,4]	1647	844 (51.24%)		1445	684 (47.34%)		202	160 (79.21%)	
(4,5]	1696	1356 (79.95%)		1515	1179 (77.82%)		181	177 (97.79%)	
(5,Inf]	428	397 (92.76%)		382	351 (91.88%)		46	46 (100%)	
missing	752	412 (54.79%)		752	412 (54.79%)				
Sequencing approach			0.0005			0.0005			1
amplicon	2480	1266 (51.05%)		2474	1261 (50.97%)		6	5 (83.33%)	
veSEQ-HIV	2043	1743 (85.32%)		1620	1365 (84.26%)		423	378 (89.36%)	

Excludes participant-visits on which deep-sequence based identification of drug resistance mutations was not attempted. Percentages show the percentage of all participant-visits within a category (columns) that have successful genotyping for all NRTIs. P-values calculated using χ^2 goodness-of-fit test with Monte Carlo simulation. Viraemic PLHIV is a sum of viraemic pretreatment PLHIV and viraemic treatment-experienced PLHIV. Viral load measurements were available for only a subset of PLHIV in the 2012 survey roads. Missing viral load measurements for pretreatment PLHIV in the 2012 survey round were imputed. All other participant-visits with missing viral load measurements were dropped. PLHIV = people living with HIV. NRTI = nucleoside reverse transcriptase inhibitors

Supplementary Table 17: Number of viraemic participant-visits with attempted deep-sequence based identification of drug resistance mutations that had successful genotyping for all PIs

	Viraemic PLWHIV			Viraemic pretreatment PLWHIV			Viraemic treatment-experienced PLWHIV		
	**Participant-visits**	**Genotype data for all PIs (%)**	***p*-value**	**Participant-visits**	**Genotype data for all PIs (%)**	***p*-value**	**Participant-visits**	**Genotype data for all PIs (%)**	***p*-value**
Overall	4523	3520 (77.82%)		4094	3127 (76.38%)		429	393 (91.61%)	
Age Category			0.57			0.62			0.94
[15,24]	950	722 (76%)		884	659 (74.55%)		66	63 (95.45%)	
[25,34]	2235	1730 (77.4%)		2034	1548 (76.11%)		201	182 (90.55%)	
[35,49]	1338	1068 (79.82%)		1176	920 (78.23%)		162	148 (91.36%)	
Community type			0.71			0.83			0.92
Agrarian	1287	1005 (78.09%)		1190	914 (76.81%)		97	91 (93.81%)	
Fishing	2246	1764 (78.54%)		1998	1536 (76.88%)		248	228 (91.94%)	
Trading	990	751 (75.86%)		906	677 (74.72%)		84	74 (88.1%)	
Sex			0.031			0.018			0.92
F	2440	1834 (75.16%)		2198	1611 (73.29%)		242	223 (92.15%)	
M	2083	1686 (80.94%)		1896	1516 (79.96%)		187	170 (90.91%)	
Survey round			0.0005			0.0005			0.71
2012	1971	1362 (69.1%)		1969	1361 (69.12%)		2	1 (50%)	
2014	798	502 (62.91%)		794	499 (62.85%)		4	3 (75%)	
2015	948	863 (91.03%)		764	706 (92.41%)		184	157 (85.33%)	
2017	635	624 (98.27%)		445	439 (98.65%)		190	185 (97.37%)	
2019	171	169 (98.83%)		122	122 (100%)		49	47 (95.92%)	
Viral load (log10 copies/mL)			0.0005			0.0005			0.26
(3,4]	1647	1079 (65.51%)		1445	910 (62.98%)		202	169 (83.66%)	
(4,5]	1696	1505 (88.74%)		1515	1327 (87.59%)		181	178 (98.34%)	
(5,Inf]	428	412 (96.26%)		382	366 (95.81%)		46	46 (100%)	
missing	752	524 (69.68%)		752	524 (69.68%)				
Sequencing approach			0.0005			0.0005			1
amplicon	2480	1674 (67.5%)		2474	1669 (67.46%)		6	5 (83.33%)	
veSEQ-HIV	2043	1846 (90.36%)		1620	1458 (90%)		423	388 (91.73%)	

Excludes participant-visits on which deep-sequence based identification of drug resistance mutations was not attempted. Percentages show the percentage of all participant-visits within a category (columns) that have successful genotyping for all PIs. P-values calculated using χ^2 goodness-of-fit test with Monte Carlo simulation. Viraemic PLHIV is a sum of viraemic pretreatment PLHIV and viraemic treatment-experienced PLHIV. Viral load measurements were available for only a subset of PLHIV in the 2012 survey roads. Missing viral load measurements for pretreatment PLHIV in the 2012 survey round were imputed. All other participant-visits with missing viral load measurements were dropped. PLWHIV = people living with HIV. PI = protease inhibitor.

Supplementary Table 18: Number of viraemic participant-visits with attempted deep-sequence based identification of drug resistance mutations that had successful genotyping for all NNRTIs, NRTIs, and PIs

	Viraemic PLWHIV			Viraemic pretreatment PLWHIV			Viraemic treatment-experienced PLWHIV		
	Participant-visits	Genotype data for all NNRTIs, NRTIs, and PIs (%)	*p*-value	Participant-visits	Genotype data for all NNRTIs, NRTIs, and PIs (%)	*p*-value	Participant-visits	Genotype data for all NNRTIs, NRTIs, and PIs (%)	*p*-value
Overall	4523	2910 (64.34%)		4094	2536 (61.94%)		429	374 (87.18%)	
Age Category			0.64			0.83			0.96
[15,24]	950	602 (63.37%)		884	543 (61.43%)		66	59 (89.39%)	
[25,34]	2235	1424 (63.71%)		2034	1251 (61.5%)		201	173 (86.07%)	
[35,49]	1338	884 (66.07%)		1176	742 (63.1%)		162	142 (87.65%)	
Community type			0.29			0.16			0.9
Agrarian	1287	865 (67.21%)		1190	777 (65.29%)		97	88 (90.72%)	
Fishing	2246	1412 (62.87%)		1998	1197 (59.91%)		248	215 (86.69%)	
Trading	990	633 (63.94%)		906	562 (62.03%)		84	71 (84.52%)	
Sex			0.0055			0.0035			1
F	2440	1491 (61.11%)		2198	1280 (58.23%)		242	211 (87.19%)	
M	2083	1419 (68.12%)		1896	1256 (66.24%)		187	163 (87.17%)	
Survey round			0.0005			0.0005			0.46
2012	1971	932 (47.29%)		1969	931 (47.28%)		2	1 (50%)	
2014	798	403 (50.5%)		794	400 (50.38%)		4	3 (75%)	
2015	948	802 (84.6%)		764	658 (86.13%)		184	144 (78.26%)	
2017	635	611 (96.22%)		445	430 (96.63%)		190	181 (95.26%)	
2019	171	162 (94.74%)		122	117 (95.9%)		49	45 (91.84%)	
Viral load (log10 copies/mL)			0.0005			0.0005			0.061
(3,4]	1647	805 (48.88%)		1445	652 (45.12%)		202	153 (75.74%)	
(4,5]	1696	1323 (78.01%)		1515	1147 (75.71%)		181	176 (97.24%)	
(5,Inf]	428	390 (91.12%)		382	345 (90.31%)		46	45 (97.83%)	
missing	752	392 (52.13%)		752	392 (52.13%)				
Sequencing approach			0.0005			0.0005			0.68
amplicon	2480	1183 (47.7%)		2474	1179 (47.66%)		6	4 (66.67%)	
veSEQ-HIV	2043	1727 (84.53%)		1620	1357 (83.77%)		423	370 (87.47%)	

Excludes participant-visits on which deep-sequence based identification of drug resistance mutations was not attempted. Percentages show the percentage of all participant-visits within a category (columns) that have successful genotyping for all NNRTIs, NRTIs, and PIs. *P*-values calculated using χ^2 goodness-of-fit test with Monte Carlo simulation. Viraemic PLHIV is a sum of viraemic pretreatment PLHIV and viraemic treatment-experienced PLHIV. Viral load measurements were available for only a subset of PLHIV in the 2012 survey roads. Missing viral load measurements for pretreatment PLHIV in the 2012 survey round were imputed. All other participant-visits with missing viral load measurements were dropped. PLHIV = people living with HIV. NNRTI = non-nucleoside reverse transcriptase inhibitors. NRTI = nucleoside reverse transcriptase inhibitors. PI = protease inhibitors.

Supplementary Table 19: Number of viraemic participant-visits with attempted deep-sequence based identification of drug resistance mutations that had successful deep-sequence based genotyping for all drugs

	Viraemic PLWHIV			Viraemic pretreatment PLWHIV			Viraemic treatment-experienced PLWHIV		
	**Participant-visits**	**Genotype data for all drugs (%)**	***p*-value**	**Participant-visits**	**Genotype data for all drugs (%)**	***p*-value**	**Participant-visits**	**Genotype data for all drugs (%)**	***p*-value**
Overall	4523	2323 (51.36%)		4094	1952 (47.68%)		429	371 (86.48%)	
Age Category			0.39			0.62			0.96
[15,24]	950	479 (50.42%)		884	420 (47.51%)		66	59 (89.39%)	
[25,34]	2235	1126 (50.38%)		2034	953 (46.85%)		201	173 (86.07%)	
[35,49]	1338	718 (53.66%)		1176	579 (49.23%)		162	139 (85.8%)	
Community type			0.089			0.012			0.81
Agrarian	1287	706 (54.86%)		1190	618 (51.93%)		97	88 (90.72%)	
Fishing	2246	1105 (49.2%)		1998	890 (44.54%)		248	215 (86.69%)	
Trading	990	512 (51.72%)		906	444 (49.01%)		84	68 (80.95%)	
Sex			0.0015			0.0005			0.92
F	2440	1170 (47.95%)		2198	962 (43.77%)		242	208 (85.95%)	
M	2083	1153 (55.35%)		1896	990 (52.22%)		187	163 (87.17%)	
Survey round			0.0005			0.0005			0.2
2012	1971	482 (24.45%)		1969	482 (24.48%)		2	0 (0%)	
2014	798	269 (33.71%)		794	267 (33.63%)		4	2 (50%)	
2015	948	799 (84.28%)		764	656 (85.86%)		184	143 (77.72%)	
2017	635	611 (96.22%)		445	430 (96.63%)		190	181 (95.26%)	
2019	171	162 (94.74%)		122	117 (95.9%)		49	45 (91.84%)	
Viral load (log10 copies/mL)			0.0005			0.0005			0.056
(3,4]	1647	656 (39.83%)		1445	504 (34.88%)		202	152 (75.25%)	
(4,5]	1696	1090 (64.27%)		1515	916 (60.46%)		181	174 (96.13%)	
(5,Inf]	428	356 (83.18%)		382	311 (81.41%)		46	45 (97.83%)	
missing	752	221 (29.39%)		752	221 (29.39%)				
Sequencing approach			0.0005			0.0005			0.19
amplicon	2480	597 (24.07%)		2474	595 (24.05%)		6	2 (33.33%)	
veSEQ-HIV	2043	1726 (84.48%)		1620	1357 (83.77%)		423	369 (87.23%)	

Excludes participant-visits on which deep-sequence based identification of drug resistance mutations was not attempted. Percentages show the percentage of all participant-visits within a category (columns) that have successful genotyping for all INSTIs, NNRTIs, NRTIs, and PIs. *P*-values calculated using χ^2 goodness-of-fit test with Monte Carlo simulation. Viraemic PLHIV is a sum of viraemic pretreatment PLHIV and viraemic treatment-experienced PLHIV. Viral load measurements were available for only a subset of PLHIV in the 2012 survey roads. Missing viral load measurements for pretreatment PLHIV in the 2012 survey round were imputed. All other participant-visits with missing viral load measurements were dropped. PLHIV = people living with HIV. INSTI = integrase strand transfer inhibitor. PI = protease inhibitor. NNRTI = non-nucleoside reverse transcriptase inhibitors. NRTI = nucleoside reverse transcriptase inhibitors. PI = protease inhibitors.

Supplementary Table 20: Number of viraemic participant-visits with resistant genotypes

Treatment-status	Class	Resistance	all	Survey Round 2012	2014	2015	2017	2019
All	INSTI	All	2578	537	335	909	627	170
		Susceptible	2546 (98.76%)	530 (98.7%)	322 (96.12%)	901 (99.12%)	623 (99.36%)	170 (100%)
		Low	13 (0.5%)	3 (0.56%)	1 (0.3%)	7 (0.77%)	2 (0.32%)	0 (0%)
		Intermediate/high	19 (0.74%)	4 (0.74%)	12 (3.58%)	1 (0.11%)	2 (0.32%)	0 (0%)
	NNRTI	All	3050	972	425	865	623	165
		Susceptible	2564 (84.07%)	891 (91.67%)	381 (89.65%)	698 (80.69%)	469 (75.28%)	125 (75.76%)
		Low	106 (3.48%)	33 (3.4%)	16 (3.76%)	30 (3.47%)	24 (3.85%)	3 (1.82%)
		Intermediate/high	380 (12.46%)	48 (4.94%)	28 (6.59%)	137 (15.84%)	130 (20.87%)	37 (22.42%)
	NRTI	All	3009	998	420	817	612	162
		Susceptible	2743 (91.16%)	962 (96.39%)	406 (96.67%)	711 (87.03%)	525 (85.78%)	139 (85.8%)
		Low	63 (2.09%)	17 (1.7%)	8 (1.9%)	19 (2.33%)	13 (2.12%)	6 (3.7%)
		Intermediate/high	203 (6.75%)	19 (1.9%)	6 (1.43%)	87 (10.65%)	74 (12.09%)	17 (10.49%)
	PI	All	3520	1362	502	863	624	169
		Susceptible	3407 (96.79%)	1316 (96.62%)	482 (96.02%)	842 (97.57%)	603 (96.63%)	164 (97.04%)
		Low	47 (1.34%)	17 (1.25%)	12 (2.39%)	7 (0.81%)	8 (1.28%)	3 (1.78%)
		Intermediate/high	66 (1.88%)	29 (2.13%)	8 (1.59%)	14 (1.62%)	13 (2.08%)	2 (1.18%)
Pretreatment	INSTI	All	2168	537	333	735	441	122
		Susceptible	2142 (98.8%)	530 (98.7%)	320 (96.1%)	731 (99.46%)	439 (99.55%)	122 (100%)
		Low	10 (0.46%)	3 (0.56%)	1 (0.3%)	4 (0.54%)	2 (0.45%)	0 (0%)
		Intermediate/high	16 (0.74%)	4 (0.74%)	12 (3.6%)	0 (0%)	0 (0%)	0 (0%)
	NNRTI	All	2656	971	422	705	439	119
		Susceptible	2383 (89.72%)	890 (91.66%)	378 (89.57%)	628 (89.08%)	381 (86.79%)	106 (89.08%)
		Low	94 (3.54%)	33 (3.4%)	16 (3.79%)	27 (3.83%)	15 (3.42%)	3 (2.52%)
		Intermediate/high	179 (6.74%)	48 (4.94%)	28 (6.64%)	50 (7.09%)	43 (9.79%)	10 (8.4%)
	NRTI	All	2626	997	417	665	430	117
		Susceptible	2518 (95.89%)	961 (96.39%)	403 (96.64%)	633 (95.19%)	411 (95.58%)	110 (94.02%)
		Low	60 (2.28%)	17 (1.71%)	8 (1.92%)	19 (2.86%)	11 (2.56%)	5 (4.27%)
		Intermediate/high	48 (1.83%)	19 (1.91%)	6 (1.44%)	13 (1.95%)	8 (1.86%)	2 (1.71%)
	PI	All	3127	1361	499	706	439	122
		Susceptible	3027 (96.8%)	1315 (96.62%)	479 (95.99%)	690 (97.73%)	423 (96.36%)	120 (98.36%)
		Low	44 (1.41%)	17 (1.25%)	12 (2.4%)	7 (0.99%)	7 (1.59%)	1 (0.82%)
		Intermediate/high	56 (1.79%)	29 (2.13%)	8 (1.6%)	9 (1.27%)	9 (2.05%)	1 (0.82%)
Treatment-experienced	INSTI	All	410		2	174	186	48
		Susceptible	404 (98.54%)		2 (100%)	170 (97.7%)	184 (98.92%)	48 (100%)
		Low	3 (0.73%)		0 (0%)	3 (1.72%)	0 (0%)	0 (0%)
		Intermediate/high	3 (0.73%)		0 (0%)	1 (0.57%)	2 (1.08%)	0 (0%)
	NNRTI	All	394	1	3	160	184	46
		Susceptible	181 (45.94%)	1 (100%)	3 (100%)	70 (43.75%)	88 (47.83%)	19 (41.3%)
		Low	12 (3.05%)	0 (0%)	0 (0%)	3 (1.88%)	9 (4.89%)	0 (0%)
		Intermediate/high	201 (51.02%)	0 (0%)	0 (0%)	87 (54.37%)	87 (47.28%)	27 (58.7%)
	NRTI	All	383	1	3	152	182	45
		Susceptible	225 (58.75%)	1 (100%)	3 (100%)	78 (51.32%)	114 (62.64%)	29 (64.44%)
		Low	3 (0.78%)	0 (0%)	0 (0%)	0 (0%)	2 (1.1%)	1 (2.22%)

		Intermediate/high	155 (40.47%)	0 (0%)	0 (0%)	74 (48.68%)	66 (36.26%)	15 (33.33%)
	PI	All	393	1	3	157	185	47
		Susceptible	380 (96.69%)	1 (100%)	3 (100%)	152 (96.82%)	180 (97.3%)	44 (93.62%)
		Low	3 (0.76%)	0 (0%)	0 (0%)	0 (0%)	1 (0.54%)	2 (4.26%)
		Intermediate/high	10 (2.54%)	0 (0%)	0 (0%)	5 (3.18%)	4 (2.16%)	1 (2.13%)

INSTI = integrase strand transfer inhibitor. PI = protease inhibitor. NNRTI = non-nucleoside reverse transcriptase inhibitors. NRTI = nucleoside reverse transcriptase inhibitors. PI = protease inhibitors.

Supplementary Table 21: Population prevalence of viraemic NNRTI, NRTI, and PI resistance by survey round

	NNRTI					NRTI					PI				
**Survey round**	***n***	**Obs (%)**	**Prev. (95% CI)**	**Prev. ratio (95% CI)**	***p*-value**	***n***	**Obs (%)**	**Prev. (95% CI)**	**Prev. ratio (95% CI)**	***p*-value**	***n***	**Obs (%)**	**Prev. (95% CI)**	**Prev. ratio (95% CI)**	***p*-value**
2015	19175	137 (0.71%)	0.88 (0.74, 1.04)	ref	ref	19127	87 (0.45%)	0.61 (0.49, 0.75)	ref	ref	19173	14 (0.07%)	0.09 (0.05, 0.15)	ref	ref
2017	19698	130 (0.66%)	0.79 (0.66, 0.93)	0.89 (0.71, 1.12)	0.32	19687	74 (0.38%)	0.46 (0.37, 0.58)	0.76 (0.57, 1.01)	0.063	19699	13 (0.07%)	0.08 (0.04, 0.13)	0.87 (0.42, 1.81)	0.71

Estimates were generated using Poisson regression with robust standard errors with survey round as a predictor variable. Generalized estimating equations with correlation structure selection by Quasi Information Criterion value (NNRTI: independent, NRTI independent, PI: independent) were used to account for repeat participants across study rounds. 95% confidence intervals indicate the Wald confidence interval around the mean value in each category. *p*-values that coefficients are different from 0 at the $\alpha = 0.05$ level were calculated using the Wald method. NNRTI = non-nucleoside reverse transcriptase inhibitors. NRTI = nucleoside reverse transcriptase inhibitors. PI = protease inhibitors.

Supplementary Table 22: Association between age, community type, and sex and the population prevalence of viraemic NNRTI, NRTI and PI resistance adjusted by survey round

		NNRTI		NRTI		PI	
Covariate	Variable	Coeff. % (95% CI)	*p*-value	Coeff. % (95% CI)	*p*-value	Coeff. % (95% CI)	*p*-value
Age	Intercept	0 (0, 0.01)	ref	0 (0, 0)	ref	0 (0, 0)	ref
	2015	0.9 (0.72, 1.12)	0.34	0.77 (0.57, 1.02)	0.068	0.88 (0.42, 1.82)	0.73
	age [25,34]	3.04 (2.18, 4.25)	<0.0001	5.55 (3.3, 9.31)	<0.0001	2.81 (1.06, 7.46)	0.039
	age [35,49]	1.99 (1.39, 2.84)	0.00018	3.69 (2.16, 6.31)	<0.0001	1.44 (0.48, 4.33)	0.51
Community type	Intercept	0.01 (0, 0.01)	ref	0 (0, 0.01)	ref	0 (0, 0)	ref
	2015	0.86 (0.69, 1.08)	0.2	0.74 (0.56, 0.98)	0.038	0.84 (0.4, 1.73)	0.63
	Fishing	3.34 (2.48, 4.51)	<0.0001	3.19 (2.15, 4.73)	<0.0001	5.33 (1.91, 14.87)	0.0014
	Trading	1.04 (0.73, 1.48)	0.82	1.08 (0.69, 1.69)	0.74	1.68 (0.53, 5.34)	0.38
Sex	Intercept	0.01 (0.01, 0.01)	ref	0.01 (0.01, 0.01)	ref	0 (0, 0)	ref
	2015	0.89 (0.71, 1.12)	0.33	0.76 (0.57, 1.02)	0.065	0.87 (0.42, 1.81)	0.71
	Male	0.74 (0.57, 0.96)	0.022	0.68 (0.48, 0.96)	0.028	0.88 (0.4, 1.92)	0.74

Estimates were generated using bivariate Poisson regression with robust standard errors with survey round and an epidemiological covariate as predictor variables. Generalized estimating equations with correlation structure selection by Quasi Information Criterion value based on the univariate analysis (NNRTI: independent, NRTI independent, PI: independent) were used to account for repeat participants across study rounds. 95% confidence intervals indicate the Wald confidence interval around the mean value in each category. p-values that coefficients are different from 0 at the $\alpha = 0.05$ level were calculated using the Wald method. NNRTI = non-nucleoside reverse transcriptase inhibitors. NRTI = nucleoside reverse transcriptase inhibitors. PI = protease inhibitors.

Supplementary Table 23: Population prevalence of viraemic NNRTI, NRTI, and PI resistance stratified by survey round and age, community type, and sex

		NNRTI					NRTI					PI				
Covariate	Strata	*n*	Obs (%)	Prev. (95% CI)	Prev. ratio (95% CI)	*p*-value	*n*	Obs (%)	Prev. (95% CI)	Prev. ratio (95% CI)	*p*-value	*n*	Obs (%)	Prev. (95% CI)	Prev. ratio (95% CI)	*p*-value
Age category	age[15,24]															
	2015	6791	29 (0.43%)	0.51 (0.35, 0.73)	ref	ref	6786	10 (0.15%)	0.19 (0.1, 0.35)	ref	ref	6792	1 (0.01%)	0.01 (0, 0.09)	ref	ref
	2017	6958	20 (0.29%)	0.33 (0.21, 0.51)	0.65 (0.37, 1.15)	0.14	6958	8 (0.11%)	0.14 (0.07, 0.27)	0.73 (0.29, 1.86)	0.51	6959	5 (0.07%)	0.08 (0.03, 0.19)	6.49 (0.76, 55.53)	0.09
	age [25,34]															
	2015	6334	63 (0.99%)	1.22 (0.95, 1.56)	ref	ref	6301	42 (0.67%)	0.91 (0.67, 1.24)	ref	ref	6327	8 (0.13%)	0.16 (0.08, 0.31)	ref	ref
	2017	6321	72 (1.14%)	1.34 (1.06, 1.68)	1.1 (0.81, 1.5)	0.55	6315	45 (0.71%)	0.87 (0.65, 1.16)	0.95 (0.65, 1.39)	0.79	6320	6 (0.09%)	0.11 (0.05, 0.24)	0.7 (0.26, 1.88)	0.49
	age [35,49]															
	2015	6050	45 (0.74%)	0.94 (0.7, 1.26)	ref	ref	6040	35 (0.58%)	0.77 (0.55, 1.07)	ref	ref	6054	5 (0.08%)	0.1 (0.04, 0.24)	ref	ref
	2017	6419	38 (0.59%)	0.73 (0.53, 1)	0.78 (0.52, 1.17)	0.23	6414	21 (0.33%)	0.42 (0.28, 0.65)	0.55 (0.32, 0.94)	0.028	6420	2 (0.03%)	0.04 (0.01, 0.15)	0.38 (0.07, 1.98)	0.25
Comm. type	Agrarian															
	2015	8757	42 (0.48%)	0.6 (0.45, 0.82)	ref	ref	8742	27 (0.31%)	0.43 (0.29, 0.62)	ref	ref	8757	4 (0.05%)	0.06 (0.02, 0.16)	ref	ref
	2017	8714	34 (0.39%)	0.47 (0.34, 0.66)	0.78 (0.51, 1.2)	0.26	8709	19 (0.22%)	0.28 (0.18, 0.43)	0.65 (0.37, 1.11)	0.12	8713	1 (0.01%)	0.01 (0, 0.1)	0.22 (0.02, 1.99)	0.18
	Fishing															
	2015	4164	66 (1.59%)	1.88 (1.48, 2.39)	ref	ref	4135	36 (0.87%)	1.14 (0.82, 1.59)	ref	ref	4158	7 (0.17%)	0.2 (0.09, 0.41)	ref	ref
	2017	4706	68 (1.44%)	1.71 (1.35, 2.16)	0.91 (0.67, 1.23)	0.54	4703	42 (0.89%)	1.09 (0.8, 1.47)	0.95 (0.64, 1.41)	0.8	4707	8 (0.17%)	0.19 (0.1, 0.39)	0.99 (0.38, 2.54)	0.98
	Trading															
	2015	6254	29 (0.46%)	0.6 (0.41, 0.86)	ref	ref	6250	24 (0.38%)	0.5 (0.34, 0.75)	ref	ref	6258	3 (0.05%)	0.05 (0.02, 0.16)	ref	ref

	2017	6278	28 (0.45%)	0.53 (0.36, 0.76)	0.88 (0.52, 1.48)	0.64	6275	13 (0.21%)	0.26 (0.15, 0.44)	0.51 (0.26, 1)	0.048	6279	4 (0.06%)	0.07 (0.03, 0.2)	1.45 (0.32, 6.63)	0.63
Sex	F											..	..	..	..	..
	2015	10368	85 (0.82%)	1.01 (0.81, 1.24)	ref	ref	10340	54 (0.52%)	0.7 (0.53, 0.91)	ref	ref	..	..	..	..	..
	2017	10505	79 (0.75%)	0.89 (0.72, 1.11)	0.89 (0.66, 1.18)	0.41	10499	48 (0.46%)	0.56 (0.42, 0.75)	0.81 (0.56, 1.16)	0.25	..	..	..	..	..
	M										..	..	..	..	..	..
	2015	8807	52 (0.59%)	0.73 (0.56, 0.96)	ref	ref	8787	33 (0.38%)	0.51 (0.36, 0.71)	ref	ref	..	..	..	..	..
	2017	9193	51 (0.55%)	0.66 (0.51, 0.87)	0.91 (0.63, 1.3)	0.6	9188	26 (0.28%)	0.35 (0.24, 0.52)	0.69 (0.43, 1.11)	0.13	..	..	..	..	..

Estimates were generated using bivariate Poisson regression with robust standard errors with survey round as a predictor variable stratified by covariate. Estimates only generated for combination of drug and covariate that were significant in the bivariate analysis. Generalized estimating equations with correlation structure selection by Quasi Information Criterion value based on the univariate analysis (NNRTI: independent, NRTI independent, PI: independent) were used to account for repeat participants across study rounds. 95% confidence intervals indicate the Wald confidence interval around the mean value in each category. *p*-values that coefficients are different from 0 at the $\alpha = 0.05$ level were calculated using the Wald method. NNRTI = non-nucleoside reverse transcriptase inhibitors. NRTI = nucleoside reverse transcriptase inhibitors. PI = protease inhibitors.

Supplementary Table 24: Population prevalence of viraemic pretreatment NNRTI, NRTI, and PI resistance by survey round

Survey round	NNRTI					NRTI					PI				
	n	Obs (%)	Prev. % (95% CI)	Prev. ratio (95% CI)	*p*-value	*n*	Obs (%)	Prev. % (95% CI)	Prev. ratio (95% CI)	*p*-value	*n*	Obs (%)	Prev. % (95% CI)	Prev. ratio (95% CI)	*p*-value
2012	16153	48 (0.3%)	0.56 (0.42, 0.75)	ref	ref	16179	19 (0.12%)	0.24 (0.15, 0.37)	ref	ref	16543	29 (0.18%)	0.25 (0.17, 0.36)	ref	ref
2014	17096	28 (0.16%)	0.4 (0.27, 0.59)	0.71 (0.44, 1.16)	0.17	17091	6 (0.04%)	0.1 (0.04, 0.22)	0.41 (0.16, 1.08)	0.071	17173	8 (0.05%)	0.11 (0.05, 0.23)	0.43 (0.19, 0.96)	0.04
2015	19219	50 (0.26%)	0.3 (0.23, 0.39)	0.54 (0.36, 0.8)	0.0023	19179	13 (0.07%)	0.09 (0.05, 0.15)	0.38 (0.19, 0.75)	0.0057	19220	9 (0.05%)	0.05 (0.03, 0.1)	0.21 (0.1, 0.46)	<0.0001
2017	19740	43 (0.22%)	0.25 (0.18, 0.33)	0.44 (0.29, 0.68)	0.00015	19731	8 (0.04%)	0.05 (0.02, 0.1)	0.21 (0.09, 0.47)	0.0002	19740	9 (0.05%)	0.05 (0.03, 0.1)	0.21 (0.1, 0.43)	<0.0001
2019	19155	10 (0.05%)	0.12 (0.06, 0.22)	0.21 (0.11, 0.43)	<0.0001	19153	2 (0.01%)	0.03 (0.01, 0.11)	0.11 (0.03, 0.49)	0.0036	19158	1 (0.01%)	0.01 (0, 0.08)	0.05 (0.01, 0.34)	0.0025

Estimates were generated using Poisson regression with robust standard errors with survey round as a predictor variable. Generalized estimating equations with correlation structure selection by Quasi Information Criterion value (NNRTI: independent, NRTI: independent, PI: independent) were used to account for repeat participants across study rounds. 95% confidence intervals indicate the Wald confidence interval around the mean value in each category. *p*-values that coefficients are different from 0 at the $\alpha = 0.05$ level were calculated using the Wald method. NNRTI = non-nucleoside reverse transcriptase inhibitors. NRTI = nucleoside reverse transcriptase inhibitors. PI = protease inhibitors.

Supplementary Figure 3: Sensitivity of results to 2012 survey round viral load missingness

Estimated prevalence of viraemic pretreatment NNRTI (left), NRTI (middle), and PI (right) resistance in the 2012 survey round among all participants (top) and among viraemic pretreatment PLHIV (bottom). Participant-visits from pretreatment PLHIV with missing viral load data were assumed to be viraemic (red), randomly imputed according to the probability of being viraemic given successful sequencing (*Supplementary Methods,* imputation used in the main analysis shown in dark blue and additional imputations shown in light blue), or non-viraemic (white). Estimates were generated using a log-Binomial generalized linear model with robust (sandwich) standard errors. 95% confidence intervals indicate the Wald confidence interval around the mean value for each estimate. NNRTI = non-nucleoside reverse transcriptase inhibitors. NRTI = nucleoside reverse transcriptase inhibitors. PI = protease inhibitors. PLHIV = people living with HIV.

Supplementary Table 25: Population prevalence of viraemic treatment-experienced NNRTI, NRTI, and PI resistance by survey round

	NNRTI					NRTI					PI				
**Survey round**	***n***	**Obs (%)**	**Prev. % (95% CI)**	**Prev. ratio (95% CI)**	***p*-value**	***n***	**Obs (%)**	**Prev. % (95% CI)**	**Prev. ratio (95% CI)**	***p*-value**	***n***	**Obs (%)**	**Prev. % (95% CI)**	**Prev. ratio (95% CI)**	***p*-value**
2015	19292	87 (0.45%)	0.58 (0.47, 0.72)	ref	ref	19284	74 (0.38%)	0.53 (0.42, 0.66)	ref	ref	19289	5 (0.03%)	0.03 (0.01, 0.08)	ref	ref
2017	19761	87 (0.44%)	0.54 (0.44, 0.66)	0.92 (0.7, 1.22)	0.57	19759	66 (0.33%)	0.42 (0.33, 0.53)	0.79 (0.58, 1.07)	0.13	19762	4 (0.02%)	0.02 (0.01, 0.06)	0.7 (0.19, 2.62)	0.6

Estimates were generated using Poisson regression with robust standard errors with survey round as a predictor variable. Generalized estimating equations with correlation structure selection by Quasi Information Criterion value (NNRTI: independent, NRTI: independent, PI: independent) were used to account for repeat participants across study rounds. 95% confidence intervals indicate the Wald confidence interval around the mean value in each category. p-values that coefficients are different from 0 at the $\alpha = 0.05$ level were calculated using the Wald method. NNRTI = non-nucleoside reverse transcriptase inhibitors. NRTI = nucleoside reverse transcriptase inhibitors. PI = protease inhibitors.

Supplementary Table 26: Population prevalence of viraemic multi-class resistance by survey round

		NNRTI				NRTI				PI			
**Survey round**	***n***	**Obs (%)**	**Prev. % (95% CI)**	**Prev. ratio (95% CI)**	***p*-value**	**Obs (%)**	**Prev. % (95% CI)**	**Prev. ratio (95% CI)**	***p*-value**	**Obs (%)**	**Prev. % (95% CI)**	**Prev. ratio (95% CI)**	***p*-value**
2015	19336	50 (0.26%)	0.33 (0.25, 0.44)	ref	ref	2 (0.01%)	0.01 (0, 0.05)	ref	ref	8 (0.04%)	0.05 (0.02, 0.1)	ref	ref
2017	19803	54 (0.27%)	0.32 (0.25, 0.42)	0.97 (0.67, 1.4)	0.86	0 (0%)	0 (0, 0)	0 (0, 0)	<0.0001	7 (0.04%)	0.04 (0.02, 0.09)	0.84 (0.3, 2.35)	0.74

		NNRTI & NRTI				NNRTI & PI				NRTI & PI			
**Survey round**	***n***	**Obs (%)**	**Prev. % (95% CI)**	**Prev. ratio (95% CI)**	***p*-value**	**Obs (%)**	**Prev. % (95% CI)**	**Prev. ratio (95% CI)**	***p*-value**	**Obs (%)**	**Prev. % (95% CI)**	**Prev. ratio (95% CI)**	***p*-value**
2015	19336	74 (0.38%)	0.54 (0.43, 0.68)	ref	ref	0 (0%)	0 (NA, NA)	ref	ref	1 (0.01%)	0.01 (0, 0.04)	ref	ref
2017	19803	70 (0.35%)	0.44 (0.35, 0.56)	0.82 (0.6, 1.11)	0.2	2 (0.01%)	0.01 (0, 0.05)	NA (NA, NA)		1 (0.01%)	0.01 (0, 0.04)	0.94 (0.06, 14.96)	0.96

		NNRTI, NRTI, & PI			
**Survey round**	***n***	**Obs (%)**	**Prev. % (95% CI)**	**Prev. ratio (95% CI)**	***p*-value**
2015	19336	5 (0.03%)	0.04 (0.02, 0.09)	ref	ref
2017	19803	3 (0.02%)	0.03 (0.01, 0.07)	0.77 (0.34, 1.74)	0.53

Estimates were generated using Poisson regression with robust standard errors with survey round as a predictor variable. Generalized estimating equations with correlation structure selection by Quasi Information Criterion value (NNRTI: independent, NRTI: independent, PI: independent, NNRTI&NRTI: independent, NNRTI&PI: independent, NRTI&PI: exchangeable, NNRTI,NRTI,&PI: exchangeable) were used to account for repeat participants across study rounds. 95% confidence intervals indicate the Wald confidence interval around the mean value in each category. p-values that coefficients are different from 0 at the $\alpha = 0.05$ level were calculated using the Wald method. NNRTI = non-nucleoside reverse transcriptase inhibitors. NRTI = nucleoside reverse transcriptase inhibitors. PI = protease inhibitors.

Supplementary Table 27: Prevalence of NNRTI, NRTI, and PI resistance among viraemic pretreatment PLHIV by survey round

	NNRTI					NRTI					PI				
Survey round	*n*	Obs (%)	Prev. % (95% CI)	Prev. ratio (95% CI)	*p*-value	*n*	Obs (%)	Prev. % (95% CI)	Prev. ratio (95% CI)	*p*-value	*n*	Obs (%)	Prev. % (95% CI)	Prev. ratio (95% CI)	*p*-value
2012	971	48 (4.94%)	4.86 (3.69, 6.42)	ref	ref	997	19 (1.91%)	1.9 (1.17, 3.11)	ref	ref	1361	29 (2.13%)	2.08 (1.43, 3)	ref	ref
2014	422	28 (6.64%)	5.46 (3.77, 7.89)	1.12 (0.72, 1.76)	0.62	417	6 (1.44%)	1.27 (0.52, 3.1)	0.67 (0.23, 1.92)	0.46	499	8 (1.6%)	1.58 (0.81, 3.08)	0.76 (0.35, 1.64)	0.48
2015	705	50 (7.09%)	7 (5.46, 8.97)	1.44 (1.01, 2.05)	0.045	665	13 (1.95%)	2.09 (1.26, 3.45)	1.1 (0.56, 2.15)	0.79	706	9 (1.27%)	1.36 (0.76, 2.4)	0.65 (0.34, 1.27)	0.21
2017	439	43 (9.79%)	9.61 (7.27, 12.7)	1.98 (1.34, 2.91)	0.00058	430	8 (1.86%)	2.05 (1.11, 3.78)	1.08 (0.49, 2.38)	0.85	439	9 (2.05%)	1.97 (1.11, 3.5)	0.95 (0.49, 1.84)	0.88
2019	119	10 (8.4%)	8.24 (4.72, 14.38)	1.69 (0.91, 3.15)	0.096	117	2 (1.71%)	1.82 (0.5, 6.6)	0.96 (0.24, 3.78)	0.95	122	1 (0.82%)	0.82 (0.15, 4.45)	0.39 (0.07, 2.23)	0.29

Estimates were generated using Poisson regression with robust standard errors with survey round as a predictor variable. Generalized estimating equations with correlation structure selection by Quasi Information Criterion value (NNRTI: exchangeable, NRTI: exchangeable, PI: AR1) were used to account for repeat participants across study rounds. 95% confidence intervals indicate the Wald confidence interval around the mean value in each category. *p*-values that coefficients are different from 0 at the $\alpha = 0.05$ level were calculated using the Wald method. NNRTI = non-nucleoside reverse transcriptase inhibitors. NRTI = nucleoside reverse transcriptase inhibitors. PI = protease inhibitors.

Supplementary Table 28: Association between age, community type, and sex and the prevalence of NNRTI, NRTI and PI resistance among viraemic pretreatment PLHIV adjusted by survey round

		NNRTI		NRTI		PI	
**Covariate**	**Variable**	**Coeff. % (95% CI)**	***p*-value**	**Coeff. % (95% CI)**	***p*-value**	**Coeff. % (95% CI)**	***p*-value**
Age	Intercept	0.05 (0.03, 0.08)	ref	0.01 (0.01, 0.04)	ref	0.02 (0.01, 0.05)	ref
	2014	1.12 (0.71, 1.76)	0.62	0.66 (0.23, 1.93)	0.45	0.75 (0.35, 1.64)	0.47
	2015	1.44 (1.01, 2.05)	0.045	1.11 (0.56, 2.17)	0.77	0.66 (0.34, 1.28)	0.22
	2017	1.97 (1.34, 2.91)	0.00063	1.09 (0.5, 2.42)	0.82	0.95 (0.49, 1.85)	0.89
	2019	1.7 (0.91, 3.15)	0.094	0.97 (0.24, 3.84)	0.96	0.4 (0.07, 2.34)	0.31
	age [25,34]	0.94 (0.63, 1.39)	0.76	1.73 (0.69, 4.3)	0.24	0.92 (0.47, 1.81)	0.81
	age [35,49]	0.91 (0.59, 1.42)	0.68	0.79 (0.27, 2.31)	0.66	0.66 (0.3, 1.43)	0.29
Community type	Intercept	0.06 (0.04, 0.08)	ref	0.02 (0.01, 0.03)	ref	0.02 (0.01, 0.04)	ref
	2014	1.12 (0.72, 1.76)	0.61	0.7 (0.24, 2.07)	0.52	0.76 (0.35, 1.64)	0.49
	2015	1.43 (1, 2.05)	0.048	1.14 (0.57, 2.25)	0.71	0.65 (0.34, 1.26)	0.21
	2017	1.97 (1.34, 2.91)	0.00061	1.09 (0.49, 2.4)	0.83	0.95 (0.49, 1.83)	0.87
	2019	1.67 (0.89, 3.13)	0.11	0.9 (0.24, 3.33)	0.88	0.39 (0.07, 2.17)	0.28
	Fishing	0.83 (0.58, 1.2)	0.32	0.79 (0.37, 1.7)	0.55	0.97 (0.47, 2.01)	0.94
	Trading	0.76 (0.48, 1.2)	0.24	1.81 (0.84, 3.9)	0.13	1.05 (0.46, 2.37)	0.92
Sequencing approach	Intercept	0.05 (0.04, 0.07)	ref	0.02 (0.01, 0.03)	ref		
	2014	1.13 (0.73, 1.75)	0.58	0.76 (0.29, 1.98)	0.57		
	2015	1.51 (0.7, 3.28)	0.3	4.13 (0.12, 137.63)	0.43		
	2017	2.08 (0.95, 4.57)	0.069	4.07 (0.12, 141.58)	0.44		
	2019	1.78 (0.7, 4.5)	0.22	3.64 (0.09, 152.86)	0.5		
	Bait capture	0.95 (0.44, 2.04)	0.89	0.25 (0.01, 8.49)	0.44		
Sex	Intercept	0.06 (0.04, 0.07)	ref	0.03 (0.02, 0.04)	ref	0.02 (0.01, 0.03)	ref
	2014	1.15 (0.73, 1.79)	0.55	0.71 (0.24, 2.06)	0.52	0.76 (0.35, 1.65)	0.49
	2015	1.47 (1.03, 2.1)	0.034	1.17 (0.59, 2.31)	0.66	0.65 (0.34, 1.27)	0.21
	2017	2.03 (1.38, 3)	0.00034	1.17 (0.53, 2.6)	0.7	0.95 (0.49, 1.85)	0.89
	2019	1.73 (0.93, 3.23)	0.083	1.01 (0.25, 4.11)	0.98	0.39 (0.07, 2.26)	0.3
	Male	0.73 (0.53, 1.02)	0.063	0.42 (0.21, 0.83)	0.013	0.98 (0.54, 1.76)	0.93

Estimates were generated using bivariate Poisson regression with robust standard errors with survey round and an epidemiological covariate as predictor variables. PI resistance was not evaluated by sequencing approach due to a limited number of outcome events. Generalized estimating equations with correlation structure selection by Quasi Information Criterion value based on the univariate analysis (NNRTI: exchangeable, NRTI: exchangeable, PI: AR1)

were used to account for repeat participants across study rounds. 95% confidence intervals indicate the Wald confidence interval around the mean value in each category. p-values that coefficients are different from 0 at the $\alpha = 0.05$ level were calculated using the Wald method. NNRTI = non-nucleoside reverse transcriptase inhibitors. NRTI = nucleoside reverse transcriptase inhibitors. PI = protease inhibitors.

Supplementary Table 29: Prevalence of NRTI resistance among viraemic pretreatment PLHIV stratified by sex

		NRTI				
**Covariate**	**Strata**	***n***	**Obs (%)**	**Prev. (95% CI)**	**Prev. ratio (95% CI)**	***p*-value**
Sex	F					
	2012	541	12 (2.22%)	2.28 (1.22, 4.29)	ref	ref
	2014	213	6 (2.82%)	2.61 (1.09, 6.25)	1.14 (0.37, 3.49)	0.82
	2015	314	10 (3.18%)	3.19 (1.76, 5.81)	1.4 (0.6, 3.28)	0.44
	2017	199	4 (2.01%)	2.22 (0.93, 5.29)	0.97 (0.32, 2.92)	0.96
	2019	60	1 (1.67%)	1.57 (0.23, 10.66)	0.69 (0.09, 5.15)	0.71
	M					
	2012	456	7 (1.54%)	1.4 (0.68, 2.87)	ref	ref
	2014	204	0 (0%)	0 (0, 0)	0 (0, 0)	<0.0001
	2015	351	3 (0.85%)	1.06 (0.41, 2.74)	0.76 (0.27, 2.14)	0.6
	2017	231	4 (1.73%)	1.84 (0.76, 4.48)	1.32 (0.43, 4)	0.63
	2019	57	1 (1.75%)	1.94 (0.34, 11.18)	1.39 (0.21, 9.04)	0.73

Estimates were generated using bivariate Poisson regression with robust standard errors with survey round as a predictor variable stratified by covariate. Estimates only generated for combination of drug and covariate that were significant in the bivariate analysis. Generalized estimating equations with correlation structure selection by Quasi Information Criterion value based on the univariate analysis (exchangeable) were used to account for repeat participants across study rounds. 95% confidence intervals indicate the Wald confidence interval around the mean value in each category. p-values that coefficients are different from 0 at the $\alpha = 0.05$ level were calculated using the Wald method. NNRTI = non-nucleoside reverse transcriptase inhibitors. NRTI = nucleoside reverse transcriptase inhibitors. PI = protease inhibitors.

Supplementary Table 30: Count of amino acid mutations observed among viraemic PLHIV

Mutation	Viraemic participant-visits		
	Total (N=2323)	Pretreatment (N=1952)	Treatment-experienced (N=371)
inT97A	257	218	39
rtK103N	203	94	109
rtM184V	161	25	136
rtE138A	98	83	15
rtY181C	77	15	62
rtG190A	74	22	52
rtM41L	71	49	22
rtK101E	64	25	39
prL33F	61	53	8
rtK65R	60	14	46
rtV108I	45	19	26
rtN348I	39	11	28
rtK70R	38	13	25
rtK219E	34	9	25
rtD67N	33	12	21
rtH221Y	33	7	26
rtV106I	31	27	4
prV32I	31	26	5
inE157Q	28	23	5
rtA98G	28	12	16
rtV179D	26	22	4
rtP225H	25	3	22
rtM184I	24	8	16
rtE138G	22	15	7
rtT215F	21	3	18
rtE138K	20	19	1
rtY115F	19	4	15
prQ58E	18	17	1
prM46I	18	15	3
rtK70E	16	4	12
rtK219Q	15	4	11
rtT215Y	15	3	12
inE92G	13	13	0
rtD67G	13	7	6
prL10F	12	12	0
rtV75I	12	9	3
rtL74I	12	1	11
rtL100I	12	0	12
prD30N	10	10	0
prK43T	10	6	4
prM46L	9	9	0
rtK219R	9	6	3
rtV106M	9	5	4
rtK238T	9	3	6
rtA62V	9	2	7
rtG190S	9	1	8
prK20T	8	6	2
inG163R	8	5	3
rtV179E	8	5	3
rtK103S	8	4	4
rtL210W	8	2	6
rtG190E	7	6	1
inQ95K	7	5	2
rtY181I	6	4	2
rtK70T	6	2	4
rtK70Q	6	1	5
rtM230L	6	0	6
rtK70N	5	5	0
rtT215S	5	5	0
rtK103T	5	4	1
rtY188L	5	4	1
prL24I	5	3	2
rtE138Q	5	3	2
rtT215I	5	3	2
rtV106A	5	1	4
rtT215V	5	0	5
rtK101P	4	4	0
inG163K	4	3	1
prG48A	4	2	2
rtF227L	4	1	3
rtY318F	4	0	4
prF53L	3	3	0
prG73T	3	3	0
rtM230I	3	3	0
rtL74V	3	2	1
rtD67T	3	1	2
rtT69D	3	0	3
prI54V	2	2	0
prL89V	2	2	0
rtT215A	2	2	0
rtT69G	2	2	0
rtV179L	2	2	0
rtV75A	2	2	0
inE138K	2	1	1
inS147G	2	1	1
prI47A	2	1	1
prI47V	2	1	1
rtK219N	2	1	1
rtL234I	2	1	1
rtV75M	2	1	1
rtY188C	2	1	1
rtK101H	2	0	2
rtK238N	2	0	2
inH51Y	1	1	0
inN155T	1	1	0
inQ148R	1	1	0
prI50L	1	1	0
prL23I	1	1	0
prN83D	1	1	0
prV82A	1	1	0
prV82L	1	1	0
rtK65E	1	1	0
rtT215E	1	1	0
rtT215L	1	1	0
rtY181F	1	1	0
rtY188H	1	1	0
inR263K	1	0	1
prV82M	1	0	1
rtD67E	1	0	1
rtD67S	1	0	1
rtG190Q	1	0	1

Supplementary Table 31: Prevalence of amino acid mutations among viraemic pretreatment PLHIV by survey round

		inT97A				rtK103N				rtE138A			
**Survey round**	***n***	**Obs (%)**	**Prev. % (95% CI)**	**Prev. ratio (95% CI)**	***p*-value**	**Obs (%)**	**Prev. % (95% CI)**	**Prev. ratio (95% CI)**	***p*-value**	**Obs (%)**	**Prev. % (95% CI)**	**Prev. ratio (95% CI)**	***p*-value**
2012	482	44 (9.13%)	9.97 (7.3, 13.61)	ref	ref	11 (2.28%)	1.67 (0.9, 3.09)	ref	ref	9 (1.87%)	2.03 (1.02, 4.02)	ref	ref
2014	267	29 (10.86%)	10.88 (7.33, 16.15)	1.09 (0.66, 1.8)	0.73	10 (3.75%)	2.46 (1.26, 4.79)	1.47 (0.59, 3.65)	0.4	7 (2.62%)	2.7 (1.18, 6.21)	1.33 (0.47, 3.78)	0.59
2015	656	62 (9.45%)	9.57 (7.53, 12.17)	0.96 (0.65, 1.42)	0.84	26 (3.96%)	3.93 (2.68, 5.76)	2.35 (1.18, 4.72)	0.016	25 (3.81%)	4.03 (2.74, 5.95)	1.99 (0.94, 4.2)	0.072
2017	430	45 (10.47%)	10.56 (8.01, 13.93)	1.06 (0.7, 1.61)	0.78	31 (7.21%)	7.11 (5.06, 10)	4.26 (2.11, 8.59)	<0.0001	12 (2.79%)	2.77 (1.59, 4.85)	1.37 (0.57, 3.3)	0.49
2019	117	24 (20.51%)	20 (13.89, 28.8)	2.01 (1.24, 3.24)	0.0044	7 (5.98%)	6.01 (2.9, 12.46)	3.6 (1.39, 9.33)	0.0085	3 (2.56%)	2.39 (0.78, 7.34)	1.18 (0.32, 4.38)	0.81

		rtM41L				prL33F				rtV179D			
**Survey round**	***n***	**Obs (%)**	**Prev. % (95% CI)**	**Prev. ratio (95% CI)**	***p*-value**	**Obs (%)**	**Prev. % (95% CI)**	**Prev. ratio (95% CI)**	***p*-value**	**Obs (%)**	**Prev. % (95% CI)**	**Prev. ratio (95% CI)**	***p*-value**
2012	482	4 (0.83%)	2.15 (0.8, 5.79)	ref	ref	4 (0.83%)	1.02 (0.36, 2.86)	ref	ref	2 (0.41%)	0.74 (0.17, 3.25)	ref	ref
2014	267	2 (0.75%)	0.69 (0.17, 2.77)	0.32 (0.06, 1.77)	0.19	4 (1.5%)	1.81 (0.51, 6.45)	1.77 (0.34, 9.08)	0.49	2 (0.75%)	1.07 (0.24, 4.76)	1.45 (0.18, 11.84)	0.73
2015	656	18 (2.74%)	2.77 (1.75, 4.39)	1.29 (0.43, 3.83)	0.65	6 (0.91%)	0.8 (0.36, 1.8)	0.78 (0.21, 2.9)	0.71	7 (1.07%)	0.91 (0.43, 1.92)	1.23 (0.24, 6.47)	0.8
2017	430	10 (2.33%)	2.41 (1.3, 4.45)	1.12 (0.35, 3.58)	0.85	7 (1.63%)	1.69 (0.81, 3.53)	1.66 (0.47, 5.87)	0.43	7 (1.63%)	1.65 (0.79, 3.45)	2.24 (0.43, 11.68)	0.34
2019	117	7 (5.98%)	5.66 (2.72, 11.77)	2.63 (0.77, 9)	0.12	2 (1.71%)	1.53 (0.38, 6.05)	1.49 (0.27, 8.34)	0.65	2 (1.71%)	2.1 (0.51, 8.65)	2.84 (0.37, 22.01)	0.32

		rtK101E				rtV108I				prQ58E			
**Survey round**	***n***	**Obs (%)**	**Prev. % (95% CI)**	**Prev. ratio (95% CI)**	***p*-value**	**Obs (%)**	**Prev. % (95% CI)**	**Prev. ratio (95% CI)**	***p*-value**	**Obs (%)**	**Prev. % (95% CI)**	**Prev. ratio (95% CI)**	***p*-value**
2012	482	4 (0.83%)	0.73 (0.27, 1.94)	ref	ref	5 (1.04%)	0.84 (0.35, 2.04)	ref	ref	3 (0.62%)	0.61 (0.19, 1.94)	ref	ref
2014	267	3 (1.12%)	1.29 (0.37, 4.43)	1.77 (0.37, 8.55)	0.48	1 (0.37%)	0.32 (0.05, 2.29)	0.38 (0.04, 3.29)	0.38	2 (0.75%)	0.26 (0.06, 1.03)	0.42 (0.07, 2.56)	0.35
2015	656	7 (1.07%)	1.15 (0.55, 2.4)	1.58 (0.46, 5.38)	0.47	5 (0.76%)	0.83 (0.35, 2.01)	0.99 (0.33, 2.96)	0.99	2 (0.3%)	0.35 (0.09, 1.39)	0.57 (0.09, 3.45)	0.54

2017	431	5 (1.16%)	1.21 (0.51, 2.9)	1.67 (0.45, 6.19)	0.44	4 (0.93%)	0.95 (0.36, 2.52)	1.12 (0.3, 4.2)	0.86	4 (0.93%)	0.94 (0.35, 2.5)	1.54 (0.34, 6.99)	0.58
2019	117	3 (2.56%)	2.27 (0.74, 7.04)	3.13 (0.7, 13.93)	0.13	0 (0%)	0 (0, 0)	0 (0, 0)	<0.0001	0 (0%)	0 (0, 0)	0 (0, 0)	<0.0001

		rtG190A			
**Survey round**	*n*	**Obs (%)**	**Prev. % (95% CI)**	**Prev. ratio (95% CI)**	***p*-value**
2012	482	4 (0.83%)	0.84 (0.29, 2.39)	ref	ref
2014	267	3 (1.12%)	1.07 (0.32, 3.5)	1.28 (0.26, 6.25)	0.76
2015	656	5 (0.76%)	0.68 (0.28, 1.63)	0.81 (0.2, 3.19)	0.76
2017	431	4 (0.93%)	0.94 (0.35, 2.5)	1.12 (0.27, 4.73)	0.87
2019	117	2 (1.71%)	1.51 (0.38, 6)	1.81 (0.32, 10.25)	0.5

Estimates were generated using Poisson regression with robust standard errors with survey round as a predictor variable. Generalized estimating equations with independent correlation structure to aid convergence for rare mutations were used. 95% confidence intervals indicate the Wald confidence interval around the mean value in each category. p-values that coefficients are different from 0 at the $\alpha = 0.05$ level were calculated using the Wald method. NNRTI = non-nucleoside reverse transcriptase inhibitors. NRTI = nucleoside reverse transcriptase inhibitors. PI = protease inhibitors.

Supplementary Figure 4: Within-host frequency of select resistance conferring amino acid mutations

Includes the 10 most prevalent (based on the 2018 survey round) resistance conferring mutations among pretreatment and viraemic treatment-experienced PLHIV.

Supplementary Table 32: Prevalence of NNRTI, NRTI, and PI resistance among viraemic treatment-experienced PLHIV by survey round

	NNRTI					NRTI					PI				
**Survey round**	***n***	**Obs (%)**	**Prev. (95% CI)**	**Prev. ratio (95% CI)**	***p*-value**	***n***	**Obs (%)**	**Prev. (95% CI)**	**Prev. ratio (95% CI)**	***p*-value**	***n***	**Obs (%)**	**Prev. (95% CI)**	**Prev. ratio (95% CI)**	***p*-value**
2015	160	87 (54.37%)	51.77 (46.47, 57.67)	ref	ref	152	74 (48.68%)	50.04 (42.59, 58.79)	ref	ref	157	5 (3.18%)	3.28 (1.38, 7.82)	ref	ref
2017	184	87 (47.28%)	51.49 (46.24, 57.34)	0.99 (0.97, 1.02)	0.72	182	66 (36.26%)	36.46 (30.06, 44.22)	0.73 (0.58, 0.92)	0.0084	185	4 (2.16%)	2.13 (0.81, 5.63)	0.65 (0.18, 2.36)	0.51

Estimates were generated using Poisson regression with robust standard errors with survey round as a predictor variable. Generalized estimating equations with correlation structure selection by Quasi Information Criterion value (NNRTI: exchangeable, NRTI: independent, PI: exchangeable) were used to account for repeat participants across study rounds. 95% confidence intervals indicate the Wald confidence interval around the mean value in each category. p-values that coefficients are different from 0 at the $\alpha = 0.05$ level were calculated using the Wald method. NNRTI = non-nucleoside reverse transcriptase inhibitors. NRTI = nucleoside reverse transcriptase inhibitors. PI = protease inhibitors.

Supplementary Table 33: Association between age, community type, and sex and the prevalence of NNRTI, NRTI and PI resistance among viraemic treatment-experienced PLHIV adjusted by survey round

		NNRTI		NRTI		PI	
**Covariate**	**Variable**	**Coeff. % (95% CI)**	***p*-value**	**Coeff. % (95% CI)**	***p*-value**	**Coeff. % (95% CI)**	***p*-value**
Age	Intercept	0.48 (0.4, 0.58)	ref	0.34 (0.22, 0.51)	ref	0.04 (0.01, 0.13)	ref
	2015	0.97 (0.93, 1.01)	0.094	0.7 (0.56, 0.89)	0.0036	0.66 (0.16, 2.74)	0.57
	age [25,34]	1.1 (0.93, 1.31)	0.27	1.62 (1.03, 2.56)	0.037	0.82 (0.13, 5.31)	0.84
	age [35,49]	1.09 (0.89, 1.35)	0.41	1.56 (0.98, 2.47)	0.061	0.8 (0.12, 5.12)	0.81
Community type	Intercept	0.52 (0.43, 0.64)	ref	0.54 (0.43, 0.69)	ref	0.03 (0.01, 0.14)	ref
	2015	1.04 (0.95, 1.14)	0.42	0.74 (0.58, 0.94)	0.013	0.65 (0.18, 2.3)	0.5
	Fishing	0.89 (0.69, 1.15)	0.39	0.86 (0.63, 1.16)	0.32	1.17 (0.24, 5.68)	0.84
	Trading	1.08 (0.81, 1.44)	0.59	0.94 (0.67, 1.33)	0.73	1.29 (0.18, 9.01)	0.8
Sex	Intercept	0.54 (0.46, 0.64)	ref	0.54 (0.45, 0.64)	ref	0.04 (0.01, 0.09)	ref
	2015	1.13 (0.89, 1.43)	0.32	0.74 (0.58, 0.94)	0.012	0.67 (0.18, 2.49)	0.55
	Male	0.74 (0.58, 0.93)	0.011	0.82 (0.62, 1.09)	0.17	0.74 (0.18, 3.04)	0.68

Estimates were generated using bivariate Poisson regression with robust standard errors with survey round and an epidemiological covariate as predictor variables. PI resistance was not evaluated by sequencing approach due to a limited number of outcome events. Generalized estimating equations with correlation structure selection by Quasi Information Criterion value based on the univariate analysis (NNRTI: exchangeable, NRTI: independent, PI: exchangeable) were used to account for repeat participants across study rounds. 95% confidence intervals indicate the Wald confidence interval around the mean value in each category. p-values that coefficients are different from 0 at the $\alpha = 0.05$ level were calculated using the Wald method. NNRTI = non-nucleoside reverse transcriptase inhibitors. NRTI = nucleoside reverse transcriptase inhibitors. PI = protease inhibitors.

Supplementary Table 34: Prevalence of NNRTI, NRTI, and PI resistance among viraemic treatment-experienced PLHIV by survey round and age, community type, and sex

		NNRTI					NRTI				
Covariate	Strata	*n*	Obs (%)	Prev. (95% CI)	Prev. ratio (95% CI)	*p*-value	*n*	Obs (%)	Prev. (95% CI)	Prev. ratio (95% CI)	*p*-value
Age category	age[15,24]										
	2015	..	..	..	..	..	31	8 (25.81%)	26.43 (14.37, 48.59)	ref	ref
	2017	..	..	..	..	..	23	8 (34.78%)	35.24 (19.94, 62.28)	1.33 (0.59, 3.01)	0.49
	age [25,34]										
	2015	..	..	..	..	..	62	33 (53.23%)	54.43 (43.16, 68.64)	ref	ref
	2017	..	..	..	..	..	97	38 (39.18%)	39.25 (30.57, 50.39)	0.72 (0.52, 0.99)	0.044
	age [35,49]										
	2015	..	..	..	..	..	59	33 (55.93%)	57.49 (46.01, 71.84)	ref	ref
	2017	..	..	..	..	..	62	20 (32.26%)	32.68 (22.74, 46.95)	0.57 (0.38, 0.85)	0.0063
Sex	F										
	2015	98	55 (56.12%)	57.03 (43.02, 75.6)	ref	ref	..	..	..	..	..
	2017	100	57 (57%)	58.17 (44.13, 76.68)	1.02 (0.62, 1.68)	0.94	..	..	..	..	..
	M						..	..	..	..	..
	2015	62	32 (51.61%)	42.27 (34.43, 51.9)	ref	ref	..	..	..	..	..
	2017	84	30 (35.71%)	43.16 (35.33, 52.71)	1.02 (0.94, 1.11)	0.62	..	..	..	..	..

Estimates were generated using bivariate Poisson regression with robust standard errors with survey round as a predictor variable stratified by covariate. Estimates only generated for combination of drug and covariate that were significant in the bivariate analysis. Generalized estimating equations with correlation structure selection by Quasi Information Criterion value based on the univariate analysis (NNRTI: exchangeable, NRTI: independent, PI: exchangeable) were used to account for repeat participants across study rounds. 95% confidence intervals indicate the Wald confidence interval around the mean value in each category. p-values that coefficients are different from 0 at the $\alpha = 0.05$ level were calculated using the Wald method. NNRTI = non-nucleoside reverse transcriptase inhibitors. NRTI = nucleoside reverse transcriptase inhibitors. PI = protease inhibitors.

Supplementary Table 35: Prevalence of amino acid mutations among viraemic treatment-experienced PLHIV

Survey round	*n*	rtM184V				rtK103N				rtY181C			
		Obs (%)	Prev. % (95% CI)	Prev. ratio (95% CI)	*p*-value	Obs (%)	Prev. % (95% CI)	Prev. ratio (95% CI)	*p*-value	Obs (%)	Prev. % (95% CI)	Prev. ratio (95% CI)	*p*-value
2015	143	57 (39.86%)	40.76 (33.32, 49.86)	ref	ref	43 (30.07%)	31.8 (24.83, 40.73)	ref	ref	31 (21.68%)	22.35 (16.34, 30.56)	ref	ref
2017	181	61 (33.7%)	33.89 (27.61, 41.59)	0.83 (0.63, 1.09)	0.19	47 (25.97%)	25.68 (20.03, 32.92)	0.81 (0.58, 1.13)	0.21	26 (14.36%)	14.67 (10.27, 20.95)	0.66 (0.43, 1.01)	0.056

Survey round	*n*	rtG190A				rtK65R				inT97A			
		Obs (%)	Prev. % (95% CI)	Prev. ratio (95% CI)	*p*-value	Obs (%)	Prev. % (95% CI)	Prev. ratio (95% CI)	*p*-value	Obs (%)	Prev. % (95% CI)	Prev. ratio (95% CI)	*p*-value
2015	143	19 (13.29%)	13.21 (8.64, 20.21)	ref	ref	20 (13.99%)	13.41 (8.85, 20.31)	ref	ref	13 (9.09%)	8.29 (4.89, 14.05)	ref	ref
2017	181	22 (12.15%)	12.34 (8.33, 18.27)	0.93 (0.55, 1.59)	0.8	22 (12.15%)	12.07 (8.14, 17.9)	0.9 (0.53, 1.53)	0.7	18 (9.94%)	9.96 (6.41, 15.48)	1.2 (0.61, 2.38)	0.6

Survey round	*n*	rtK101E				rtK219E				rtH221Y			
		Obs (%)	Prev. % (95% CI)	Prev. ratio (95% CI)	*p*-value	Obs (%)	Prev. % (95% CI)	Prev. ratio (95% CI)	*p*-value	Obs (%)	Prev. % (95% CI)	Prev. ratio (95% CI)	*p*-value
2015	143	15 (10.49%)	10.1 (6.21, 16.44)	ref	ref	10 (6.99%)	6.93 (3.78, 12.7)	ref	ref	11 (7.69%)	7.41 (4.17, 13.19)	ref	ref
2017	181	17 (9.39%)	9.51 (6.04, 14.97)	0.94 (0.51, 1.75)	0.85	14 (7.73%)	7.64 (4.6, 12.67)	1.1 (0.51, 2.36)	0.8	13 (7.18%)	7.35 (4.34, 12.43)	0.99 (0.45, 2.16)	0.98

Survey round	*n*	rtV108I			
		Obs (%)	Prev. % (95% CI)	Prev. ratio (95% CI)	*p*-value
2015	143	8 (5.59%)	6.33 (3.23, 12.42)	ref	ref
2017	181	13 (7.18%)	7.08 (4.18, 11.99)	1.12 (0.5, 2.52)	0.79

Estimates were generated using Poisson regression with robust standard errors with survey round as a predictor variable. Generalized estimating equations with independent correlation structure to aid convergence for rare mutations were used. 95% confidence intervals indicate the Wald confidence interval around the mean value in each category. p-values that coefficients are different from 0 at the $\alpha = 0.05$ level were calculated using the Wald method. NNRTI = non-nucleoside reverse transcriptase inhibitors. NRTI = nucleoside reverse transcriptase inhibitors. PI = protease inhibitors.

Supplementary References

1 Monod M, Brizzi A, Ssekubugu R, *et al.* Growing gender disparity in HIV infection in Africa: sources and policy implications. .

2 Gall A, Ferns B, Morris C, *et al.* Universal Amplification, Next-Generation Sequencing, and Assembly of HIV-1 Genomes. *J Clin Microbiol* 2012; **50**: 3838–44.

3 Bonsall D, Golubchik T, de Cesare M, *et al.* A Comprehensive Genomics Solution for HIV Surveillance and Clinical Monitoring in Low-Income Settings. *J Clin Microbiol* 2020; **58**: e00382-20.

4 Bolger AM, Lohse M, Usadel B. Trimmomatic: A flexible trimmer for Illumina sequence data. *Bioinformatics* 2014; **30**: 2114–20.

5 Broad Institute. Picard tools. Https://Broadinstitute.Github.Io/Picard/. 2016. https://broadinstitute.github.io/picard/%5Cnhttp://broadinstitute.github.io/picard/.

6 McGinnis S, Madden TL. BLAST: at the core of a powerful and diverse set of sequence analysis tools. *Nucleic Acids Res* 2004; **32**: W20–5.

7 Ji H, Enns E, Brumme CJ, *et al.* Bioinformatic data processing pipelines in support of next-generation sequencing-based HIV drug resistance testing: the Winnipeg Consensus. *J Int AIDS Soc* 2018; **21**: e25193.

8 Fogel JM, Bonsall D, Cummings V, *et al.* Performance of a high-throughput next-generation sequencing method for analysis of HIV drug resistance and viral load. *J Antimicrob Chemother* 2020; **75**: 3510–6.

9 Rhee S-Y. Human immunodeficiency virus reverse transcriptase and protease sequence database. *Nucleic Acids Res* 2003; **31**: 298–303.

10 Shafer RW. Rationale and Uses of a Public HIV Drug-Resistance Database. *J Infect Dis* 2006; **194**: S51–8.

11 Liu TF, Shafer RW. Web Resources for HIV Type 1 Genotypic-Resistance Test Interpretation. *Clin Infect Dis* 2006; **42**: 1608–18.

12 Zou G. A Modified Poisson Regression Approach to Prospective Studies with Binary Data. *Am J Epidemiol* 2004; **159**: 702–6.

13 R Core Team. R: A Language and Environment for Statistical Computing. Vienna, Australia, 2020 https://www.R-project.org/ (accessed Dec 11, 2020).

14 Halekoh U, Højsgaard S, Yan J. The *R* Package **geepack** for Generalized Estimating Equations. *J Stat Softw* 2006; **15**. DOI:10.18637/jss.v015.i02.

15 Lenth RV. emmeans: Estimated Marginal Means, aka Least-Squares Means. 2024 https://CRAN.R-project.org/package=emmeans.

16 Wickham H, Averick M, Bryan J, *et al.* Welcome to the Tidyverse. *J Open Source Softw* 2019; **4**: 1686.

17 Wickham H, Chang W, Henry L, *et al.* ggplot2: Elegant Graphics for Data Analysis. Springer-Verlag New York, 2021 http://ggplot2.org (accessed Feb 8, 2022).

18 Wilke CO. cowplot: Streamlined Plot Theme and Plot Annotations for ‘ggplot2’. 2020. https://CRAN.R-project.org/package=cowplot.

19 Lin Pedersen T. patchwork: The Composer of Plots. 2023. https://cran.r-project.org/web/packages/patchwork/index.html.

20 FC M, Davis TL, ggplot2 authors. ggpattern: ‘ggplot2’ Pattern Geoms. 2022. https://github.com/coolbutuseless/ggpattern.

21 Wickham H, Bryan J. readxl: Read Excel Files. 2023. https://readxl.tidyverse.org.

22 Wickham H, Miller E, Smith D. haven: Import and Export ‘SPSS’, ‘Stata’ and ‘SAS’ Files. 2023. https://github.com/tidyverse/haven.
