# Supplementary material for: HIV drug resistance during antiretroviral therapy scale-up in Uganda, 2012–19: a population-based, longitudinal study
Source: Lancet Microbe. 2025 Dec;6(12):None. doi: 10.1016/j.lanmic.2025.101218 (PMC12722182; doi:10.1016/j.lanmic.2025.101218)
Supplement: Supplementary appendix 2 [file mmc2.pdf]

THE LANCET
Microbe

Supplementary appendix 2

This appendix formed part of the original submission and has been peer reviewed. We post it as supplied by the authors.

Supplement to: Martin MA, Reynolds SJ, Foley BT, et al. HIV drug resistance during antiretroviral therapy scale-up in Uganda, 2012–19: a population-based, longitudinal study. *Lancet Microbe* 2025. https://doi.org/10.1016/j.lanmic.2025.101218

RAKAI COMMUNITY COHORT STUDY (RCCS)
ROUND 19 BASELINE MALE ENGLISH QUESTIONNAIRE ver 1.1 12TH JUNE 2018

IDENTIFICATION SHEET FOR MALE BASELINE QUESTIONNAIRE 1.1 12th JUNE 2018

INTERVIEWER # |__|__|__|__|__|{ FIELD_WRKR }

INTERVIEW DATE {INT DATE }
___/___/20____
dd mm yyyy

PLACE COMPUTER ID HERE

VISIT # |R|19|{VISITNO}
Current ID
Super Cluster # |__|__|__|{REGION}
Community # |__|__|__|{COMM_NUM}
HH |__|__|__|__|__|{HH_NUM}
Member # |__|__|__|{MEMBER_NUM}

SEX **M**
National id no: ____________________________ {NATION ID}

Names: Usually called ______________________________ {NAMES}
Religious names ______________________________
Other names ______________________________

Mother's maiden name: ________________________________ {NAME_MOT}

Q.1 How old are you? **(Age in completed years.)**
(Valid range 15-49) |__|__|{AGEYRS}

Q.2 How long have you lived in this community?

**if < 1 week code days**	DAYS	|__|__|{RESIDDYS}
**if < 1 month code weeks**	WEEKS	|__|__|{RESIDWKS}
**if < 1 year code months, else completed years**	MONTHS	|__|__|{RESIDMOS}
	YEARS	|__|__|{RESIDYRS}

[If DK for all Code 97 in days and 98 in other boxes; 99 for NR
91<2 years, 92: 2-4 years, 93:5-9 years, 94: 10 and above.

IF RESPONDENT IS AN INMIGRANT IN ALREADY EXISITNG HOUSEHOLD/STRUCTURE (BY R 12) AND INTEND TO STAY DO AN INTERVIEW

ALL original official copies MUST be kept and ONLY edited by Data Management

RAKAI COMMUNITY COHORT STUDY (RCCS)
ROUND 19 BASELINE MALE ENGLISH QUESTIONNAIRE ver 1.1 12TH JUNE 2018

INTERVIEWER # |__|__|__|__|__| {FIELD_WRKR}

INTERVIEW DATE {INT DATE}
___/___/20__
dd mm yyyy

PLACE
COMPUTER ID HERE

VISIT # |_R_|19|{VISITNO}

Current ID
Super Cluster # |__|__|__|__|{REGION}
Community # |__|__|__|__|{COMM_NUM}
HH # |__|__|__|__|__|{HH_NUM}
Member # |__|__|__|__|{MEMBER_NUM}

SEX **M**

TIME STARTED |__|__|:|__|__| am/pm {START}

Q.1 How old are you? **(Age in completed years.)**
(Valid range 15-49) |__|__|{AGEYRS}

Q.2 What is your birth date?
(check birth date vs age, reconcile the two if needed code 99 for any unknown dd, mm, yy)

Day	|__|__|{BIRTHDY}
Month	|__|__|{BIRTHMO}
Year	|__|__|__|__|{BIRTHYR}

Q.3 What is your religion?

None	1	|__|{RELIGION}
Catholic	2	
Protestant (inc. Church of Uganda)	3	
Saved/Pentecostal	4	
Muslim	5	
Other **(specify)**__ ____________________	6	{OTHRELIG}

Q.4 Have you ever gone to school?

Yes	1	|__|{EDUCATE}
No	2------------>**Q.6**	

ALL original official copies MUST be kept and ONLY edited by Data Management

Q.5a To what level **(probe if got additional training)**?

(Code highest level)

P1-P4	0 1	|__|{EDUCYRS)
P5-P7	02	
S1-S4	03	
S5-S6	04	
Technical/University	05	
Primary professional	06	
O'level professional	07	
Primary or O'level apprenticeship	10	
A'level apprenticeship	11	

FOR THOSE 15-24 years please ask 5b else skip to Q.6

Q.5b Are you currently enrolled in school?

Yes 1 ------------Q.5d |__| {SCHOOL}
No 2

Note: Code Yes in Q.5b applies for primary, secondary or university/Tertiary, not to vocational /apprenticeship or informal training programs.

Q.5c If not currently enrolled or left school before completing why did you leave school ?*[INTERVIEWER READ ALL OPTIONS AND MARK YES OR NO]*

	Yes	No	
a. Financial	1	2	{FINSCH}
b. Impregnated	1	2	{PREGSCH}
c. Moved	1	2	{MOVESCH}
f. Sexual harassment in or on the way to school	1	2	{HARASCH}
j. Bullying on the way or in to school	1	2	{BULLWSCH}
i. Parent died	1	2	{PARESCH}
j. Accomplished my educational goals	1	2	{ACCOMSCH}
k. Other	1	2	{OTHSCH}

Specify -- {SPESCH}

Q.5d Did you get any other additional training or apprenticeship such as hair dressing, mechanics carpentry welding , construction etc?

Yes 1 |__|{ADDTRAIN}
No 2

Q.5e Do you use a mobile phone to do the following?

	Yes	No	
a. Get information about health or medicine for you or your family (clinic location or contact)	1	2	{PHONEHEALTH}
b. Contact romantic partners	1	2	{PHONEPARTNER}
c. Access transportation	1	2	{PHONETRANS}
d. Work related (find employment, complete work tasks)	1	2	{PHONEWORK}
e. Access the Internet, use Whatsapp, Facebook or other apps	1	2	{PHONEMEDIA}

Q.6 What kind of work do you do, or what kind of activities keep you busy during an average day, whether you get money from them or not?

(Record answer(s) as given):________________________________ **(OCCUP AT1)**

________________________________ **(OCCUPAT 2)**

(Code up to two responses, code first mentioned occupation first)

Agriculture for home use/barter	01	|__|__|{OCCUP1}
Agriculture for selling	02	|__|__|{OCCUP2}
Housework in your own home	03	
Housekeeper (for relative or employer)	04	
Home brewing	05	
Government/clerical/teaching	06	
Fishing	07	
Student	08	
Military/police	09	
Shopkeeper	10	
Trading/vending	11	
Bar worker or owner	12	
Trucker	13	
Unemployed (**PROBE _ NO AGRIC OR HOUSE WORK?**)	14	
Other (specify)______________________	15	{OCCS}
No additional occupation (use in 2nd field if one occupation is cited)	88	
Medical worker (non-government)	16	
Casual laborer	17	
Waitress/Waiter/restaurant owner	18	
Hair dresser/Salon owner	19	
Construction (brick maker, builder, porter, painter, roofing)	20	
Mechanic (automobiles, bicycles, electronics)	21	
Boda Boda	22	
Sports betting, Gambling Machine, Ludo	24	

I WOULD LIKE TO ASK YOU SOME QUESTIONS ABOUT WAYS IN WHCH PEOPLE PLAN THEIR FAMILIES IN RAKAI AND NEIGHBOURING COMMUNITIES

Q.7a Do you have any living children? |__|{LIVECHILD}

Yes 1

No 2 -------------------->Q.8a

Q.7b How many living children do you have? |__|__|{NUMCHILD}

I WOULD LIKE TO ASK YOU SOME QUESTIONS ABOUT FAMILY PLANNING

Q.8a Are you/your partner currently using any family planning method?

Yes 1
No 2 |___|(fpusing)

Q.8b) If yes what method are you currently using? (Un prompted)

For those using calender method, code a backup method

	**Current us**			
	Yes	No	DK	
Pill	1	2	7	{fpusing1}
Condom	1	2	7	{fpusing2}
Injection	1	2	7	{fpusing4}
IUD / coil	1	2	7	{fpusing7}
Norplant	1	2	7	{fpusng12}
Tubal ligation/vasectomy	1	2	7	{fpusng11}
Spermicide	1	2	7	{fpusing3}
Abstinence	1	2	7	{fpusing5}
Calendar/Rhythm	1	2	7	{fpusing6}
Breast-feeding	1	2	7	{fpusing9}
Herbs/traditional medicine	1	2	7	{fpusng10}
Other	1	2	7	{fpusing8}
Specify ________________________	{OTHFP}			

Q.9 Have you ever been married or entered a consensual union?

(If present union is the ONLY marriage ever,

Yes 1 |__| {EVERMARR}
No 2---------->**Q.**14
DK 7---------->**Q.**14

Q.10 Are you currently married (whether traditional, civil, or religious, or in a consensual union)?

Yes 1 |__|{CURRMARR}
No 2-------------------->**Q.12**

Q.11. How many wives do you have? |__|__|{POLYMAR}

[Record actual # wherever possible, otherwise
Code 92= a few (01-02), 93=a lot/many(03+), no response= 99]
[For currently married ask]

IF YES OR EVER MARRIED ASK 12 ELSE SKIP TO 14

Q.12 Have you had any marital or consensual relationship that ended? |__| {EVERMEND}
Yes 1
No 2------------------->Q.14

Q. 13 . How did your previous marital or consensual relationship end?
(***Most Recently Ended Marriage First***)

Separated	2	|__|{PSTAT1}
Divorced	3	|__|{PSTAT2}
Widowed/Partner died	4	|__|{PSTAT3}
		|__|{PSTAT4}

Q.14 Do you currently have a relationship with someone to whom you are not officially married or in a consensual union?

Yes	1	|__| {CURRRLTN}
No	2	
DK	7	

Q.15 Have you had sexual intercourse with any person in the last 12 months?

Yes	1	|__|{SEXYEAR}
No	2-------------------->**Q.19a**	
NR	9	

For questions 16-19a if more than 69 partners code 93 and write actual number besides the box, or else code actual number

Q.16 How many different sexual partners have you had in the last 12 months, including married or consensual partners and anyone already mentioned? |__|__|{SEXP1YR}
[Record actual # wherever possible, otherwise
code 92= a few (01-02), 93=a lot/many(03+), no response= 99]

Q.18 How many partners in the last twelve months were from outside this community? |__|__|{SEXP1OUT}
[Record actual # wherever possible, otherwise
Code 92= a few (01-02), 93=a lot/many(03+), no response= 99]

Q.19a How many different sexual partners have you had in your lifetime including married or consensual partners? |__|__|{SEXPEVER}
[valid codes: Record actual # wherever possible, otherwise
code 92= a few (01-02), 93=a lot/many(03+), no response= 99]

FOR THOSE 15-24 years please ask Q.19b else skip to Q.20

Q.19b How old were you when you first developed pubic hair? **|__|__|** {PUBYEARS}
(Record completed years, Code DK=97 NR=99 no pubic hair =88)

IF NEVER MARRIED OR IN A RELATIONSHIP AND NO SEXUAL

PARTNER IN THE 12 months (i.e. Q.9-19bARE NO/00)
THEN ASK Q.20 ELSE SKIP TO Q.21

Q.20 Have you ever had a sexual relationship? Yes 1 |__|{EVERSEX}
No 2--------->**Q.120**

FIRST SEXUAL EXPERIENCE: MEN

Q.21a How old were you the first time you had sexual intercourse? |__|__| {AG1STSX}
[Record completed years, DK=97, NR=99]

BEGIN REPITITIVE SEXUAL PARTNER BLOCKS

FIRST BLOCK

(The following blocks of questions should be asked for each current and past relationship in the last 12 months up to 4 partners. Begin by asking about the most recent partner. If the most recent relationship was more than a year ago, still ask about this most recent partner).

"Now I would like to ask you about your most recent sex partners. Please remember that all of your answers are confidential. Your answers are very important to our research to help us understand health problems in Rakai and its neighbouring districts".

Q.22 Remembering the most recent time you had sex, what was your relationship to that partner at that time?

Current wife(at the time)	01	|__|__| {RLTN1}
Current consensual partner(at the time)	02	
Former wife/consensual partner	03	
Girlfriend	04	
Occasional or casual friend	05	
Visitor (incl. wedding/funeral)	06	
Stranger	07	
Workmate	08	
Boss/work supervisor	09	
Employee	10	
Fellow student	11	
Sugar mummy	12	
Relative other than spouse (specify) _________	13	{OTHRLTN1}
Other non relative **(specify)**________________	14	{OTHRLNR1}
Don't Know	97	
Client/Sex worker	16	

Insert spouse''s household curr ID if spouse is in the study area, relationship still ongoing and married at time of sex. If Spouse is not in study area code 888.888.8888.888

Spouse' current ID |__|__|__|/ |__|__|__| /|__|__|__|__| /|__|__|__| {P1CURR_ID}

COMPUTER ID

CURRENT ID |__|__|__|/ |__|__|__| /|__|__|__|__| /|__|__|__|

Q.23 What are/were your partner's main occupations?
(Record answer(s) as given):_________________(OCCUPAT11)
_________________(OCCUPAT21)
(Code up to two responses, code first mentioned occupation first.)

Agriculture for home use/barter	01	|__|__|{OCCUP11}
Agriculture for selling	02	|__|__|{OCCUP21}
Housework in own home	03	
Housekeeper (for relative or employer)	04	
Home brewing	05	
Government/clerical/teaching	06	
Fishing	07	
Student	08	
Military/police	09	
Shopkeeper	10	
Trading/vending	11	
Bar worker or owner	12	
Trucker	13	
Unemployed (**PROBE _ NO AGRIC OR HOUSE WORK?**)	14	
Other ___________________________	15	{OCCUPS1}
No additional response(use in 2nd field if one occupation is cited	88	
Do Not Know	97	
Medical worker (non-government)	16	
Casual laborer	17	
Waitress/Waiter/restaurant owner	18	
Hair dresser/Salon owner	19	
Construction (brick laying, building)	20	
Mechanic (automobiles, bicycles, electronics)	21	
Boda Boda	22	
Client/Sex worker	23	
Sports betting, Gambling Machine, Ludo	24	

Q.24 How long ago did you first have sex with this person?

**Less than 1 day code 00, Less than 1 week code**	days	|__|__|{DAYS1}
**Less than one month code weeks**	weeks	|__|__|{WEEKS1}
**Less than one year code months**	months	|__|__|{MONTHS1}
**If 1 year or more code completed Years**	Years	|__|__|{YEARS1}

(If DK Code 97 in days and 98 in other boxes; 99 for NR)

Q.25 How long ago, did you last have sex with this person?

Less than 1 day code 00 days, less than 1 week code days |__|__|{RLDYSLT1}
Less than 1 month code weeks weeks |__|__|{RLWKSLT1}
Less than 1 year code months months |__|__|{RLMOSLT1}
If one year or more code completed years years |__|__|{RLYRSLT1}
(If DK Code 97 in days and 98 in other boxes; 99 for NR)

Q.26 Are you still in a sexual relationship with her?

Yes	1	|__| {RLTONGO1}
No	2	
Don't know	7	

Q.27 Does he live (living) in this household ? |__|{RLTNHH1 }

Yes	1--------→Q29b
No	2

Q.29a Does(was)she regularly live(living) in your community? |__| {RLTNCM1}

Yes	1
No	2
DK	7

Q.29b Is/was she older, younger, or about the same age?

Older	1	|__| {RLTNAGE1}
Younger	2	
Same age	3---------------->**Q.31**	
DK	7---------------->**Q.31**	

Q.29c About how many years [older/younger]? |__|__|{RLTNYRS1}
(Record actual # or 97=don't know)

During the last time you were having sexual relations with this partner, please tell me how often you usually had intercourse.

Q.31. How many times a week do/did you usually have sex? |__|__|{SEXWK1}
[Record actual #, code 97 for DK, 99 for NR]

Q.32. How many times a month do/did you have sexual intercourse with this person? |__|__|{SEXMT1}
[Record actual #, code 97 for DK, 99 for NR]

Q.33. How many months in the past 12 months did/have you had sexual intercourse? |__|__|{ MTSMON1Y1}
(Code # of months in the past 12 months, if relationship is more than 12 month ago code 00)

Q.34 Have you and this partner ever used a condom?

Yes	1	|__|{CNDEVER1}
No	2------------------>**Q.38**	
NR	9------------------>Q.38	

Q.35 During the most recent 12 months you were having sexual relationship with this partner, how often did you use condoms? |__|{RNYRCON1}

Never	1**----------->Q.38**

ALL original official copies MUST be kept and ONLY edited by Data Management

Sometimes/inconsistent 2
Always 3
DK 7----------->Q.38

Q.36 Did you use a condom the last time you had sex with that partner?

Yes 1 |__|{RLTNLST1}
No 2
DK/DR 7

Q. 38. Were money, gifts, or favors ever exchanged for sex with this partner?

Yes, Recieved only 1 |__|{SEXGIFTP1

Yes, Gave only 2

Yes, Gave and received 3

No 4

Q.39"Is the primary reason you had a sexual relationship with this partner because you expected financial support from him (such as money for personal needs, looking after your children, paying your rent, starting a business etc.)?

Yes 1 |__|{SEXFINANC1}
No 2

Q.41 At any time in the past, has your partner informed you about her HIV status whether positive or not?

Yes 1 |__|{KNWHIVEVR1}
No 2
DK/NR 7

Q.42 Have you ever informed your partner of your HIV status whether positive or not?

Yes 1 |__|{INFYPEVER1}
No 2
Never tested/got HIV results 3
Received couple counseling 4
(Results for both individuals, marital or non-marital, should have been delivered)
DR 7

Q.43a Have you ever received HIV results with this partner? |__|{ CPLECONS1}

Yes 1
No 2

SECOND BLOCK

"Can you tell me about the sexual partner just prior to the one we just discussed?"
(In the last twelve months)

Q.44 Have you had any other sexual partners in the past 12 months other than the one we just discussed? |__|{MORE2}

Yes 1

ALL original official copies MUST be kept and ONLY edited by Data Management

No 2-------->**Q.113**

Q.45 Remembering the most recent time you had sex, what was your relationship to that partner at that time?

Current wife (at the time)	01	|__|__| {RLTN2}
Current consensual partner (at the time)	02	
Former wife/consensual	03	
Girlfriend	04	
Occasional or casual friend	05	
Visitor (incl. wedding/funeral)	06	
Stranger	07	
Workmate	08	
Boss/work supervisor	09	
Employee	10	
Fellow student	11	
Sugar mummy	12	
Relative other than spouse (specify)________	13	{OTHRLTN2}
Other non relative **(specify)________________**	14	{OTHRLNR2}
Don't Know	97	
Client/Sex worker	16	

Insert spouse' household curr ID if spouse is in the study area, relationship still ongoing and married at time of sex. If spouse is not in study area code 888.888.8888.888

Spouse's current ID |__|__|__|/ |__|__|__| /|__|__|__|__| /|__|__|__|{P2CURR_ID}

Q. 46 What are/were your partner's main occupations?
(Record answer(s) as given):__________________(OCCUPAT12)
__________________(OCCUPAT22)
(Code up to two responses, code first mentioned occupation first.)

Agriculture for home use/barter	01	|__|__|{OCCUP12}
Agriculture for selling	02	|__|__|{OCCUP22}
Housework in own home	03	
Housekeeper (for relative or employer)	04	
Home brewing	05	
Government/clerical/teaching	06	
Fishing	07	
Student	08	
Military/police	09	
Shopkeeper	10	
Trading/vending	11	
Bar worker or owner	12	
Trucker	13	
Unemployed (**PROBE _ NO AGRIC OR HOUSE WORK?**)	14	
Other ___________________________	15	{OCCUPS2}
No additional response(use in 2nd field if one occupation is cited	88	
Do Not Know	97	
Medical worker (non-government)	16	

Casual laborer 17
Waitress/Waiter/restaurant owner 18
Hair dresser/Salon owner 19
Construction (brick maker, painter, builder,roofing, porter) 20
Mechanic (automobiles, bicycles, electronics) 21
Boda Boda 22
Client/Sex worker 23
Sports betting, Gambling Machine, Ludo 24

Q.47 How long ago did you first have sex with this person?

**Less than 1 day code 00, Less than 1 week code**	days	|__|__|{DAYS2}
**Less than one month code weeks**	weeks	|__|__|{WEEKS2}
**Less than one year code months**	months	|__|__|{MONTHS2}
**If 1 year or more code completed Years**	Years	|__|__|{YEARS2}

(If DK Code 97 in days and 98 in other boxes; 99 for NR)

Q.48 How long ago, did you last have sex with this person?

**Less than 1 day code 00 days, less than 1 week code**	days	|__|__|{RLDYSLT2}
**Less than 1 month code weeks**	weeks	|__|__|{RLWKSLT2}
**Less than 1 year code months**	months	|__|__|{RLMOSLT2}

(If DK Code 97 in days and 98 in other boxes; 99 for NR)

Q.49 Are you still in a sexual relationship with her?

Yes 1 |__| {RLTONGO2}
No 2

Q.50 Does he live (living) in this household ? |__|{RLTNHH2 }

Yes 1--------→Q.52b
No 2

Q.52a. Does(was)she regularly live(living) in your community? |__| {RLTNCM2}

Yes 1
No 2
DK 7

Q.52b Is/was she older, younger, or about the same age?

Older 1 |__| {RLTNAGE2}
Younger 2
Same age 3---------------->**Q.54**
DK 7---------------->**Q.54**

Q.52c. About how many years [older/younger]? |__|__|{RLTNYRS2}
(Record actual # or 97=don't know)

DURING THE LAST TIME YOU WERE HAVING SEXUAL RELATIONS WITH THIS PARTNER, PLEASE TELL ME HOW OFTEN YOU USUALLY HAD INTERCOURSE.

Q.54. How many times a week do/did you usually have sex? |__|__|{ SEXWK2}
[Record actual #, code 97 for DK, 99 for NR]

Q.55. How many times a month do/did you have sexual intercourse with this person? |__|__|{ SEXMT2}
[Record actual #, code 97 for DK, 99 for NR]

Q.56. How many months in the past 12 months did/have you had sexual intercourse? |__|__|{ MTSMON2Y1}
(Code # of months in the past 12 months)

Q.57 Have you and this partner ever used a condom? |__|{CNDEVER2}

Yes 1
No 2---------------->**Q.60**
NR 9-------------->Q.60

Q.58 During the most recent 12 months you were having sexual relationship with this partner, how often did you use condoms? |__|{RNYRCON2}

Never 1**------------>Q.60**
Sometimes/inconsistent 2
Always 3
DK 7-------------->Q.60

Q.59 Did you use a condom the last time you had sex with that partner? |__|{RLTNLST2}

Yes 1
No 2
DK/DR 7

Q.60. Were money gifts, or favors ever exchanged for sex with this partner? |__|{SEXGIFTP2

Yes, Received only 1
Yes, Gave only 2
Yes, Gave and received 3
No 4

Q.62"Is the primary reason you had a sexual relationship with this partner because you expected financial support from him (such as money for personal needs, looking after your children, paying your rent, starting a business etc.)? |__|{SEXFINANC2}

Yes 1
No 2

Q.64 At any time in the past, has your partner informed you about her HIV status whether positive or negative? |__|{KNWHIVEVR2}

Yes 1
No 2
NR 7

Q.65 Have you ever informed your partner of your HIV status whether positive or negative?

Yes 1 |__| {INFYPEVER2}
No 2
Never tested/got HIV results 3
Received couple counseling 4
(Results for both individuals, marital or non-marital, should have been delivered)
DR 7

Q.66a Have you ever received HIV results with this partner? |__|{CPLECONS2}
Yes 1
No 2

ALL original official copies MUST be kept and ONLY edited by Data Management

THIRD BLOCK

"Can you tell me about the sexual partner just prior to the one we just discussed?"
(In the last twelve month)

Q.67 Have you had any other sexual partners in the past 12 months other than the one we just discussed?

Yes	1	|__| {MORE3}
No	2----------->**Q.1**13	

Q.68 Remembering the most recent time you had sex, what was your relationship to that partner at that time?

Current wife (at the time)	01	|__|__| {RLTN3}
Current consensual partner (at the time)	02	
Former wife/consensual	03	
Girlfriend	04	
Occasional or casual friend	05	
Visitor (incl. wedding/funeral)	06	
Stranger	07	
Workmate	08	
Boss/work supervisor	09	
Employee	10	
Fellow student	11	
Sugar mummy	12	
Relative other than spouse (specify) _________	13	{OTHRLTN3}
Other non relative **(specify)**_________________	14	{OTHRLNR3}
Don't Know	97	
Client/Sex worker	16	

Insert spouse' household Curr ID if spouse is in the study area, relationship still ongoing and married at time of sex. If spouse is not in study area code 888.888.8888.888

Spouse' current ID |__|__|__|/ |__|__|__| /|__|__|__|__| /|__|__|__| {P3CURR_ID}

Q.69 What are / were your partner's main occupations?
(Record answer(s) as given):__________________(OCCUPAT13)
__________________(OCCUPAT23)
(Code up to two responses, code first mentioned occupation first.)

Agriculture for home use/barter	01	|__|__|{OCCUP13}
Agriculture for selling	02	|__|__|{OCCUP23}
Housework in own home	03	
Housekeeper (for relative or employer)	04	
Home brewing	05	
Government/clerical/teaching	06	
Fishing	07	
Student	08	
Military/police	09	
Shopkeeper	10	
Trading/vending	11	
Bar worker or owner	12	
Trucker	13	
Unemployed (**PROBE _ NO AGRIC OR HOUSE WORK?**)	14	
Other ________________________	15	{OCCUPS3}
No additional response (use in 2nd field if one occupation is cited	88	
Do Not Know	97	
Medical worker (non-government)	16	
Casual laborer	17	
Waitress/Waiter/restaurant owner	18	
Hair dresser/Salon owner	19	
Construction (brick maker, builder, porter, painter, roofing)	20	
Mechanic (automobiles, bicycles, electronics)	21	
Boda Boda	22	
Client/Sex worker	23	
Sports betting, Gambling Machine, Ludo	24	

Q.70 How long ago did you first have sex with this person?

**Less than 1 day code 00, Less than 1 week code**	days	|__|__|{DAYS3}
**Less than one month code weeks**	weeks	|__|__|{WEEKS3}
**Less than one year code months**	months	|__|__|{MONTHS3}
**If 1 year or more code completed Years**	years	|__|__|{YEARS3}

(If DK Code 97 in days and 98 in other boxes; 99 for NR)

Q.71 How long ago, did you last have sex with this person?

**Less than 1 day code 00 days, less than 1 week code**	days	|__|__|{RLDYSLT3}
**Less than 1 month code weeks**	Weeks	|__|__|{RLWKSLT3}
**Less than 1 year code months**	months	|__|__|{RLMOSLT3}

(If DK Code 97 in days and 98 in other boxes; 99 for NR)

Q.72 Are you still in a sexual relationship with her?

Yes	1	|__| {RLTONGO3}
No	2	
DK	7	

ALL original official copies MUST be kept and ONLY edited by Data Management

Q.73 Does he live (living) in this household ? |__|{RLTNHH3 }

Yes 1--------→Q.75b
No 2

Q.75a Does(was)she regularly live(living) in your community? |__| {RLTNCM3}

Yes 1
No 2
DK 7

Q.75b Is/was she older, younger, or about the same age?

Older 1 |__| {RLTNAGE3}
Younger 2
Same age 3---------------->**Q.77**
DK 7---------------->**Q.77**

Q.75c. About how many years [older/younger]? |__|__|{RLTNYRS3}
(Record actual # or 97=don't know)

During the last time you were having sexual relations with this partner, please tell me how often you usually had intercourse.

Q.77.How many times a week do/did you usually have sex? |__|__|{ SEXWK3}
[Record actual #, code 97 for DK, 99 for NR]

Q.78 How many times a month do/did you have sexual intercourse with this person? |__|__|{SEXMT3}
[Record actual #, code 97 for DK, 99 for NR]

Q.79 How many months in the past 12 months did/have you had sexual intercourse?|__|__|{ MTSMON3Y1}
(Code # of months in the past 12 months)

Q.80 Have you and this partner ever used a condom? |__|{CNDEVER3}

Yes 1---------→Q.83
No 2
NR 9

Q.81 During the most recent 12 months you were having sexual relationship with this partner, how often did you use condoms? |__|{RNYRCON3}

Never 1**------------>Q.83**
Sometimes/inconsistent 2
Always 3
DK 7--------->Q.83

Q.82 Did you use a condom the last time you had sex with that partner?

Yes 1 |__|{RLTNLST3}
No 2
DK/DR 7

ALL original official copies MUST be kept and ONLY edited by Data Management

Q.83 Were money gifts, or favors ever exchanged for sex with this partner?

Yes, Received only 1 |__|{SEXGIFTP3}

Yes, Gave only 2

Yes, Gave and received 3

No 4

Q.85 "Is the primary reason you had a sexual relationship with this partner because you expected financial support from him (such as money for personal needs, looking after your children, paying your rent, starting a business etc.)?

Yes 1 |__|{SEXFINANC3}
No 2

Q.87 At any time in the past, has your partner informed you about her HIV status whether positive or negative? |__|{KNWHIVEVR3}

Yes 1
No 2
NR 7

Q.88 Have you ever informed your partner of your HIV status whether positive or negative?

Yes 1 |__| {INFYPEVER3}
No 2
Never tested/got HIV results 3
Received couple counseling 4
DR 7

Q.89a Have you ever received HIV results with this partner? |__|{CPLECONS3}

Yes 1
No 2

FOURTH BLOCK

"Can you tell me about the sexual partner just prior to the one we just discussed?"
(In the last twelve month)

Q.90 Have you had any other sexual partners in the past 12 months other than the one we just discussed? |__|{MORE4}

Yes	1	
No	2----------->**Q.113**	

Q.91 Remembering the most recent time you had sex, what was your relationship to that partner at that time?

Current wife (at the time)	01	|__|__| {RLTN4}
Current consensual partner (at the time)	02	
Former wife/consensual	03	
Girlfriend	04	
Occasional or casual friend	05	
Visitor (incl. wedding/funeral)	06	
Stranger	07	
Workmate	08	
Boss/work supervisor	09	
Employee	10	
Fellow student	11	
Sugar mummy	12	
Relative other than spouse (specify) _________	13	{OTHRLTN4}
Other non relative **(specify)**________________	14	{OTHRLNR4}
Don't Know	97	
Client/Sex worker	15	

Insert spouse' household curr ID if spouse is in the study area, relationship still ongoing and married at the time of sex. If spouse is not in study area code 888.888.8888.888

Spouse' CURRENT ID |__|__|__|/ |__|__|__| /|__|__|__|__| /|__|__|__| **{P4CURR_ID}**

ALL original official copies MUST be kept and ONLY edited by Data Management

Q.92 What are/were your partner's main occupations?
(Record answer(s) as given):______________________________________(OCCUPAT14)
______________________________________(OCCUPAT24)
(Code up to two responses, code first mentioned occupation first.)

Agriculture for home use/barter	01	|__|__|{OCCUP14}
Agriculture for selling	02	|__|__|{OCCUP24}
Housework in own home	03	
Housekeeper (for relative or employer)	04	
Home brewing	05	
Government/clerical/teaching	06	
Fishing	07	
Student	08	
Military/police	09	
Shopkeeper	10	
Trading/vending	11	
Bar worker or owner	12	
Trucker	13	
Unemployed (**PROBE _ NO AGRIC OR HOUSE WORK?**)	14	
Other ___________________________	15	
No additional response (use in 2nd field if one occupation is cited	88	
Do Not Know	97	
Medical worker (non-government)	16	
Casual laborer	17	
Waitress/Waiter/restaurant owner	18	
Hair dresser/Salon owner	19	
Construction (brick maker, builder, painter, porter, roofing)	20	
Mechanic (automobiles, bicycles, electronics)	21	
Boda Boda	22	
Client/Sex worker	23	
Sports betting, Gambling Machine, Ludo	24	

Q.93 How long ago did you first have sex with this person?

**Less than 1 day code 00, Less than 1 week code**	days	|__|__|{DAYS4}
**Less than one month code weeks**	weeks	|__|__|{WEEKS4}
**Less than one year code months**	months	|__|__|{MONTHS4}
**If 1 year or more code completed Years**	Years	|__|__|{YEARS4}

(If DK Code 97 in days and 98 in other boxes; 99 for NR)

Q.94 How long ago, did you last have sex with this person?

**Less than 1 day code 00 days, less than 1 week code**	days	|__|__|{RLDYSLT4}
**Less than 1 month code weeks**	weeks	|__|__|{RLWKSLT4}
**Less than 1 year code months**	months	|__|__|{RLMOSLT4}

(If DK Code 97 in days and 98 in other boxes; 99 for NR)

Q.95 Are you still in a sexual relationship with her?

Yes	1	|__| {RLTONGO4}
No	2	
DK	7	

Q.96 Does he live (living) in this household ? |__|{RLTNHH4 }

Yes 1 --------→Q.98b
No 2

Q.98a. Does (was) she regularly live (living) in your community? |__| {RLTNCM4}

Yes 1
No 2
DK 7

Q.98b.Is/was she older, younger, or about the same age?

Older 1 |__| {RLTNAGE4}
Younger 2
Same age 3---------------->**Q.100**
DK 7---------------->**Q.100**

Q.98c. About how many years [older/younger]? |__|__|{RLTNYRS4}
(Record actual # or 97=don't know)

During the last time you were having sexual relations with this partner, please tell me how often you usually had intercourse.

Q.100. How many times a week do/did you usually have sex? |__|__|{ SEXWK4}
[Record actual #, code 97 for DK, 99 for NR]

Q.101 How many times a month do/did you have sexual intercourse with this person? **[Record actual #, code 97 for DK, 99 for NR]** |__|__|{ SEXMT4}

Q.102. How many months in the past 12 months did/have you had sexual intercourse?
(Code # of months in the past 12 months, if relationship is more than 12 month ago code 00) |__|__|{ MTSMON4Y1}

Q.103 Have you and this partner ever used a condom? |__|{CNDEVER4}

Yes 1
No 2------------->**Q.106**
NR 9------------->**Q.106**

Q.104 During the most recent 12 months you were having sexual relationship with this partner, how often did you use condoms? |__|{RNYRCON4}

Never 1**-------------->Q.106**
Sometimes/inconsistent 2
Always 3
DK 7------------->**Q.106**

Q.105 Did you use a condom the last time you had sex with that partner?

Yes 1 |__|{RLTNLST4}
No 2
DK/DR 7

ALL original official copies MUST be kept and ONLY edited by Data Management

Q. 106. Were money, gifts, or favors ever exchanged for sex with this partner?

Yes, Received only	1	|__|{SEXGIFTP4}
Yes, Gave only	2	
Yes, Gave and received	3	
No	4	

Q.108"Is the primary reason you had a sexual relationship with this partner because you expected financial support from him (such as money for personal needs, looking after your children, paying your rent, starting a business etc.)?

Yes	1	|__|{SEXFINANC4}
No	2	

Q.110 At any time in the past, has your partner informed you about her HIV status whether positive or negative? |__|{ KNWHIVEVER4 }

Yes	1
No	2
NR	7

Q.111 Have you ever informed your partner of your HIV status whether positive or negative?

Yes	1	|__|{INFYPEVER4}
No	2	
Never tested/got HIV results	3	
Received couple counseling	4	

(Results for both individuals, marital or non-marital, should have been delivered)

DR	7

Q.112 Have you ever received HIV results with this partner? |__|{CPLECONS4}

Yes	1
No	2

END OF REPITITVE SEXUAL PARTNER BLOCKS.

IF NOT MARRIED AND NO SEX IN LAST 12 MONTHS SKIP TO Q.117

DISAGREEMENTS ON SOME ISSUES CAN OCCUR BETWEEN MEN and WOMEN, WHICH SOMETIMES RESULT INTO VIOLENCE. I WOULD LIKE TO ASK YOU SOME QUESTIONS ON VIOLENCE.

Q.113 In the past 12 months did you do any of the following to any of your partners: **PROMPTED**

	Yes	No	NA	
Verbally abuse or shout at her?	1	2	8	{PABUSEYR}
Push her, slap her or hold her down?	1	2	8	{PPUSHYR}
Punch her with fist or with something that could hurt her?	1	2	8	{PFISTYR}
Kick her or drag her?	1	2	8	{PKICKYR}
Threatened her with a weapon (knife, gun, fire, rope)	1	2	8	{PWEAPYR}
Attacked her with a weapon?	1	2	8	{PATKNYR}
Other	1	2	8	{POTHVYR}

Specify________________________ {OTHPVYRS}

ALL original official copies MUST be kept and ONLY edited by Data Management

Q.114 In the past 12 months, did you do any of the following to any of your sexual partner :

**[PROMPTED]**	Yes	No	NA	NR	
Used threats to force her to have sex when she did not want to?	1	2	8	9	{VTHRSXPY}
Physically forced her to have sex when she did not want to?	1	2	8	9	{PFOCSXPY}
Forced her to perform sexual acts she did not want to do?	1	2	8	9	{OTHFSXPY}

Q 115 Was any of the partner pregnant at the time of the abuse?

Yes	1	|__| {PREGABU}
No	2	
NA	8	

IF RESPONDENT HAS REPORTED RECENT VIOLENCE i.e. Respondent has a "YES" in 113 or Q.114, ASK 116, ELSE SKIP TO 117

Q 116 During the most recent episode of physical or sexual violence, please tell me whether you or your partner or both of you were under the influence of alcohol (prompted)

Yes-male partner only	1	|__| {ALCPRTVIO}
Yes-female partner only	2	
Yes – both partners	3	
No – neither	4	
DK/DR	7	
No response	9	

Q.117 Do/did you drink alcohol before sex with any of your partners? |__|{ALCRBSX}

Yes	1
No	2
NR	7

Q.118 Does/did any of your partners drink alcohol before sex? |__| {ALCPBSX}

Yes	1
No	2
NR	7

Q.119 How likely do you think you are to acquire HIV from any of your partner/s? (**prompted**)

Very likely	1	|__|{SAIDS}
Somewhat likely	2	
Unlikely	3	
Not at all	4	
Don't Know	7	

I AM NOW GOING TO ASK YOU SOME QUESTIONS ABOUT HIV TESTING. PLEASE KNOW THAT WHATEVER ANSWERS YOU PROVIDE WILL BE KEPT STRICTLY CONFIDENTIAL.

Q.120 Have you ever received your HIV results from any where? |__|{ RHIVEVER}

Yes 1
No 2-------------->Q.127
N/A(Never tested) 8-------------->Q.127
NR 9-------------->Q.127

Q.121 How long ago did you last receive your last HIV results?

Within the past 1 year 1 |__|{HIVPERIOD}
1-2 years 2
3-4years 3
>4 years 4

Q. 122a What was the result of this last HIV test?

Negative – 1 |__| {HIVRSLT}
Positive – 2
Indeterminate – 3
No Response – 9
Don't know / don't remember – 7

Q.122b Have you ever heard about a way to prevent HIV which involves an HIV-negative person taking a daily pill to reduce their risk of acquiring HIV while in a sexual relationship with someone who might be HIV-positive? |__| {KNOWPEP}

Yes 1
No 2

Q.122c Have you ever used PreP? |__|{PREPEVER}

Yes 1
No 2-------------Q.123

Q.122d Are you currently using PreP? |__|{PREPUSE}

Yes 1------Q.123
No 2

Q.122e [If yes to Q122c and no to Q.22d] Why did you stop using PreP?

	YES	NO	NA
Pill burden ……………...	1	2	8 {RESNOPREP1}
Side effects………………………………………	1	2	8 {RESNOPREP2}
Did not know I had to take them ………………….	1	2	8 {RESNOPREP3}
Lack of medicine……………………………………	1	2	8 {RESNOPREP4}
Stigma …………………………………………	1	2	8 {RESNOPREP5}
Clinic too far away………………………………	1	2	8 {RESNOPREP6}
Too much time consuming………………………...	1	2	8 {RESNOPREP7}
Did not feel I was at risk for getting HIV…………	1	2	8 {RESNOPREP8}
Did not think they would work……………………..	1	2	8 {RESNOPREP9}

Did not want to be mistaken for being HIV-positive	1	2	8	{RESNOPREP10}
Using other HIV prevention methods (e.g.condoms)	1	2	8	{RESNOPREP11}
Ended a relationship/no longer having sex	1	2	8	{RESNOPREP12}
Not ready……………………………………….	1	2	8	{RESNOPREP14}
Stock out………………………………………..	1	2	8	{RESNOPREP15}
Trouble transferring from one clinic to another…..	1	2	8	{RESNOPREP16}
Other………………………………………….	1	2	8	{RESNOPREP13}

specify ---{ OTHRESNOPREP}

FOR PERSONS WHO HAVE EVER HAD VCT (I.e., RESPONSE TO Qn 120 IS YES) ASK Qn 123 ELSE SKIP TO 127

WE WANT TO ASK YOU ABOUT USE OF HEALTH CARE SERVICES AND MEDICATION. THIS INFORMATION WILL BE STRICTLY CONFIDENTIAL. YOU DO NOT HAVE TO ANSWER ANY OF THESE QUESTIONS.

Q123 Have you ever been to a clinic to receive care for HIV? |__|{HIVCARE2}

Yes 1
No 2
DK 7
NR 9

Q.124a Have you ever been on the following long term medications?
Q.124b Are you currently taking this medication?
Q.124c What is your current source of this medication?

<table>
<tr><th></th><th colspan="4">Ever</th><th></th><th colspan="4">Current</th><th></th><th colspan="3" rowspan="2">Current source of this medication?</th></tr>
<tr><th></th><th>Yes</th><th>No</th><th>DK</th><th>NR</th><th></th><th>Yes</th><th>No</th><th>DK</th><th>NR</th><th></th></tr>
<tr><td>ARVs</td><td>1</td><td>2</td><td>7</td><td>9</td><td>{ARVMED}</td><td>1</td><td>2</td><td>7</td><td>9</td><td></td><td>{CUARVMED}</td><td>|__|__|__|</td><td>{ARVSOURC2}</td></tr>
<tr><td>Septrin / Dapsone</td><td>1</td><td>2</td><td>7</td><td>9</td><td>{SEPTMED}</td><td>1</td><td>2</td><td>7</td><td>9</td><td></td><td>{CUSEPTMED}</td><td>|__|__|__|</td><td>{SEPTSOURC2}</td></tr>
</table>

Other arv current source ______________________________________ {OTHARVSOU1}
Other Septrin or dapsone current source____________________________________ {OTHSEPSOU1}
{OTHSEPSOU1}

Other clinic type for current medication

Gov't 1 |__|{ARVTYPE1}
NGO 2 |__|{SEPTTYPE1}
Private 3

FOR THOSE WHO ANSWER YES TO BEING HIV POSITIVE in Q.122a or EVER TAKEN ARVs AND NOT TO BEING CURRENTLY ON ARVS ASK Q.124d ELSE SKIP TO Q.127

Q.124d What are the 3 main reasons why you are not taking ARVs?

Felt well ……………………………………………………….02 |__|__| { RESNOARV1}
Pill burden ………….. 03 |__|__| {RESNOARV2}
Side effects…………………………………………………….04 |__|__| {RESNOARV3}
Did not know I had to take them ……………………………05
Lack of medicine……………………………………………06
Stigma ……………………………………………………….. 07
Clinic too far away…………………………………………09
Too much time consuming……………………................ 10
Other ……………………………………………… ………11
Fear of side effects……………………………………..12
Did not have my medicine ……………………………..13
Not ready…………………………………………………14
Stock out…………………………………………………. 15
Trouble transferring from one clinic to another……………16
No additional response…………………………………… 88
Not applicable………………………………….. ………98
specify ……………………………………………{OTHNOARV}

HEALTH UNITS CODES FOR RAKAI DISTRICT 2008 TO DATE

NAME OF HEALTH UNIT	CODE	OWNERSHIP
Baale Gunda	037	GOV'T
Bakka	104	GOV'T
Bethrehem	010	GOV'T
Bikira	002	NGO
Bitabago	047	PRIVATE
Bugona	099	GOV'T
Bulamu	100	GOV'T
Butembe	078	GOV'T
Butiti	098	GOV'T
Buyamba Dwaniro	028	GOV'T
Buyamba NGO	050	NGO
Buyiisa	072	GOV'T
Buziranduulu	015	GOV'T
Byakabanda	076	GOV'T
Byerima	095	GOV'T

Gayaaza	005	GOV'T
Gwanda	085	GOV'T
Heal the nation	044	NGO
Kabira	012	GOV'T
Kabusota	070	GOV'T
Kabuwoko H/C	006	GOV'T
Kabuwoko NGO	007	NGO
Kacheera	031	GOV'T
Kagamba	027	GOV'T
Kakundi	033	GOV'T
Kakuuto	016	GOV'T
Kaleere	034	GOV'T
Kalisizo H/C	087	NGO
Kalisizo T.C/Hosp	001	GOV'T
Kalisizo/Kyango	014	GOV'T
Kasaali	004	GOV'T
Kasankala	055	GOV'T
Kasankala NGO	059	NGO
Kasasa	023	GOV'T
Kasensero	089	GOV'T
Katatenga	049	GOV'T
Kayanja	063	GOV'T
Kayanja Prison	045	GOV'T
Kayayumbe	048	NGO
Kayonza	035	GOV'T
Kayonza Ddwaniro	103	GOV'T
Kibaale H/C II	030	GOV'T
Kibaale Home	067	NGO
Kibanda	022	GOV'T
Kibanda Clinic	068	PRIVATE
Kibuuka	011	GOV'T
Kifamba	021	GOV'T
Kijjeja	086	GOV'T

Kijonjo	097	GOV'T
Kimuli	041	GOV'T
Kirumba	101	GOV'T
Kiziba	051	GOV'T
Kyabigondo	057	GOV'T
Kyakanyomozi	079	GOV'T
Kyakonda	074	GOV'T
Kyakuwa	092	GOV'T
Kyalulangira	065	GOV'T
Kyebe	018	GOV'T
Kyempewo	102	GOV'T
Kyotera G/C	071	PRIVATE
Kyotera M/C (Katwire)	080	PRIVATE
Kyotera Moslems	075	NGO
Kyotera T.C/Mitukula(Kamwanyi)	024	GOV'T
Lukerere	061	GOV'T
Lwabakooba	056	GOV'T
Lwakaloolo	054	GOV'T
Lwamaggwa NGO	069	NGO
Lwamaggwwa	029	GOV'T
Lwamba	036	GOV'T
Lwanda	025	GOV'T
Lwankoni	042	GOV'T
Lwembajjo	052	GOV'T
Lwensinga	060	GOV'T
Magabi	082	GOV'T
Mayanja	090	GOV'T
Mbuye	026	NGO
Micungiro	038	GOV'T
Minziiro	040	GOV'T
Mirigwe	094	GOV'T
Mukisa	064	PRIVATE
Mutukula	017	GOV'T

Muzito	081	PRIVATE
Nabigasa	009	GOV'T
Nabyajjwe	062	GOV'T
Nakasoga	084	GOV'T
Nakatoogo	008	GOV'T
Nangoma	020	GOV'T
Nazareth	088	NGO
Ndolo	039	GOV'T
Nkenge	053	GOV'T
No additional Response	888	
Not applicable	998	
Others	115	
Rakai Program Kalisizo Hub	105	NGO
Rakai hospital	032	GOV'T
Sanje Dom	046	PRIVATE
Sserulunda	013	GOV'T
St. Bernard Mannya	093	PRIVATE
St. Joseph	083	PRIVATE
St. Martine	043	PRIVATE
St.Gyaviira	058	PRIVATE
St.Mugagga	073	PRIVATE

IF EVER ON ARV MEDICATION ASK 125a ELSE SKIP TO 127

Q.125a Some people buy ,sale and some times share ARVS ?**[Prompted]**

	YES	NO
Have you ever bought ARVs-------------	1	2 {SHAREARVS1}
Have you ever sold ARVs -------------	1	2 {SHAREARVS2}
Have you ever shared ARVs--------------	1	2{SHAREARVS3}
Some one else shared their ARVs with you ------	1	2{SHAREARVS4}

ALL original official copies MUST be kept and ONLY edited by Data Management

Q.125b [If yes to Q125a above], who did you buy/sell/share antiretroviral drugs with? [Mark all that apply]

[Mark all that apply]

	YES	NO
Spouse/sexual partner------------------	1	2 {WHOSHARE1}
Other family member------------------	1	2 {WHOSHARE2}
Friend------------------------------------	1	2 {WHOSHARE3}
Work colleague-------------------------	1	2{WHOSHARE4}
Stranger----------------------------------	1	2{WHOSHARE5}
Other-------------------------------------	1	2{WHOSHARE6}

Specify ---{WHOSOTH}

Q.125c In the past twelve months ?**[Prompted]**

	YES	NO
Have you ever bought ARVs-------------	1	2 {SHAREARVS12M}
Have you ever sold ARVs -------------	1	2 {SHAREARVS22M}
Have you ever shared ARVs--------------	1	2{SHAREARVS32M}
Some one else shared their ARVs with you ------	1	2{SHAREARVS42M}

Q.125d [If yes to Q125c above], who did you buy/sell/share antiretroviral drugs with? [Mark all that apply]

[Mark all that apply]

	YES	NO
Spouse/sexual partner------------------	1	2 {WHOSHARE12M}
Other family member------------------	1	2 {WHOSHARE22M}
Friend------------------------------------	1	2 {WHOSHARE32M}
Work colleague-------------------------	1	2{WHOSHARE42M}
Stranger----------------------------------	1	2{WHOSHARE52M}
Other-------------------------------------	1	2{WHOSHARE62M}

Specify ---{WHOSOTH2M}

Q.127. During the last 30 days, have you had any of the following health problems? **PROMPTED:**

	Yes	No	NR	
Substantial weight loss (more than 3kgs) in the last one month	1	2	9	{SYMP02}
Fever ≥3 weeks	1	2	9	{SYMP07}
Cough ≥2 weeks	1	2	9	{SYMP10}
Tuberculosis	1	2	9	{SYMP16}
Coughed up sputum stained with blood	1	2	9	{SYMP26}
Excessive night sweats for ≥3 weeks	1	2	9	{SYMP27}
Genital ulcer	1	2	9	{SXM1}

if yes to any of the following(weight loss, fever, cough, TB, cough with blood, or night sweats refer for sputum and TB testing)

Q.128 Has anyone in your household or place of work had TB in the last 30 days? |__| {HOUSETB}

Yes 1 (If yes to refer for TB testing)

No 2
DK 7

I WOULD LIKE TO ASK YOU A FEW QUESTIONS ABOUT TAKING ALCOHOL

Q.130 Have you drunk any alcohol in the past year, for instance, beer, wine, waragi or other spirits, or home-made beer?

Yes 1 |__| {DRINKALC}
No 2-------------->Skip to Q.133

Q131. When is the last time you took alcohol?

Less than 1 day code 00, Less than 1 week code Days |__|__| {ALCDAY}
Less than one month code weeks Weeks |__|__|{ALCWEEK}
Less than one year code months Months |__|__|{ALCMONTH}
(If DK Code 97 in days and 98 in other boxes; 99 for NR)

Q132. In the past year, when you drank alcohol, do you ever have/get the following? PROMPT

	Yes	No	N/R	
Unsteady gait	1	2	9	{SALCGAIT}
Fell over	1	2	9	{SALCFALL}
Got angry	1	2	9	{SALCVIOL}
Got violent/got into a fight	1	2	9	{SALCFIGHT}
Had difficulty speaking	1	2	9	{SALCSPEA}
Forgot some of the things that you did or that happened while you were drinking	1	2	9	{ SALCFORG }
Have shaking hands the next morning	1	2	9	{SALCSHAKE}
Felt ashamed of something that you did while drinking	1	2	9	{SALCSHAME}

MALE CIRCUMCISION

NOW I WOULD LIKE TO ASK YOU SOME QUESTIONS ABOUT MALE CIRCUMCISION. THESE QUESTIONS WILL HELP US TO UNDERSTAND PEOPLE'S OPINIONS ABOUT MALE CIRCUMCISION, AND TO IMPROVE MALE CIRCUMCISION SERVICES

Q. 133 If there was a free program to have babies circumcised in their first 6 weeks of life to protect them against future HIV acquisition, would you be willing to have your baby circumcised?

Yes 1 |__|{BABYCIRCM}
No 2

Q. 134.Are you circumcised? |__|{CIRCUM}

Yes 1
No 2 → **Q.142**

Q. 135 Where were you circumcised from? |___| {MCIRCPLA}

Rakai Program 1
Gov't Health Unit 2
Private practitioner 3
Other (specify)____ 4 {OMCIRCPL}
DK 7

Q. 136 What were the main reasons you wanted to be circumcised? |___|{WHYCIRC}

Traditional/Religious 1
Health 2
Other (specify)_____________ 3
Personal hygiene 4 {OTHWHYC}
DK/NR 7

Q.137. How long ago were you circumcised?

Days |__|__| PDCIRCY1}
Weeks |__|__| {PWCIRCY1}
Months|__|__| {PMCIRCY1}
Year |__|__| {PYCIRCY1}

Q.138 Have you had sex with any one since you were circumcised? |__|{SEXOPY1}

Yes 1
No 2. → Q.140
NR 9

Q.139. How soon after circumcision did you resume sex?

DAYS |__|__| [SEXSONDYY1]
WEEKS |__|__| [SEXSONWKY1]
MONTHS |__|__| [SEXSONMOY1]
YEAR |__|__| [SEXSONYR Y1]

ALL original official copies MUST be kept and ONLY edited by Data Management

For all men circumcised

Q.140, Before getting MC did you discuss MC with that partner at that time

Yes 1 |__| {PARTDISCUS}
No 2……………………………………………….>Q.146
NA ((Short term partner/no partner at that time) 8…………..>Q.146

Q.141 Was (were) the partner (partners)

Supportive	1	|__| {CIRCPART}
Neutral	2	
Negative towards MC	3	
Some partners encouraged others discouraged -------	4	
NR	9	

For uncircumcised males ask;

Q.142, Have you ever discussed male circumcision with your partner?

Yes	1	|__|{CIRCDISCUS}
No	2 ——————► Q144	
NA(no partner)	8 ………..> Q144	

Q.143 Was the partner

Supportive	1	|__| {CIRCWIFE}
Neutral	2	
Negative towards MC	3	
Some partner supported others did not support	4	
NR	9	
DK	7	

Q.144 You have indicated that you are not circumcised; would you be willing to accept circumcision since the procedure is offered free through the Rakai Program? |__| {ACCPTCCMF}

Yes	1-------------- > 146 Refer to surgical team.
No	2
Unsure / Undecided	3 -------------- > 146
Parent to decide	4 -------------- > 146
No Response	9 -------------- > 146

Q.145 Why wouldn't you be willing to accept circumcision although the procedure is offered free through the Rakai Program?

	Yes	No
Fear of injury	1	2 {HCIRCNORE1}
Fear of pain	1	2 {HCIRCNORE2}
Against religious beliefs	1	2{HCIRCNORE3}
I am not at risk of HIV	1	2{HCIRCNORE4}
Against the traditional beliefs	1	2{HCIRCNORE5}
Other	1	2{HCIRCNORE12}

Specify______________________________________ {HCIRCNOROT}

I WOULD LIKE TO ASK YOU A QUESTION CONCERNING SMOKING.

Q. 146 Do you smoke cigarettes, tobacco or a pipe?

Yes 1 |__|{SMOKE}
No 2

Q147 Have you used any of these drugs in the past 12 months?

	YES	NO
Marijuana	1	2{UNARC12M1}
Amphetamines	1	2{UNARC12M2}
Aero fuels ("glue")	1	2{UNARC12M3}
Amayirungi(Khat)	1	2{UNARC12M4}
Heroin	1	2{ UNARC12M6}
Kubber	1	2{ UNARC12M7}
Sisha	1	2{ UNARC12M8}
Other	1	2{UNARC12M5}

(specify) ______________________ {OTNARC12M}

FOR RESPONDENTS AGED 35-49 YEARS ASK 148 ELSE SKIP TO PARTNER IDENTIFICATION

I WOULD LIKE TO ASK QUESTIONS ABOUT DISEASES / ILLNESS WHICH MAKES A PERSON TAKE DRUGS FOR A LONG TIME

Q148. Have you ever been diagnosed with High blood pressure?

Yes 1 |__| {PRESSURE}
No 2-------→ Q150
DK 7-------→ Q150

For those diagnosed with high blood pressure ask

Q.149 Are you taking antihypertensive medication regularly? |__|{HYPERMED}

Yes 1
No 2

Q.150. Have you ever been diagnosed with diabetes?

Yes 1 |__| {DIABETES}
No 2-------→ Q152
DK 7-------→ Q152

For those diagnosed with high blood sugar ask

Q.151 Are you taking any medicine against diabetes regularly? |__|{MEDDIABET}

Yes 1
No 2

Q. 152. Have any of your family members been diagnosed withdiabetes or high blood sugar?

If yes Probe (Father, Mother, Biological Children, Siblings)

Yes 1 |__| {FDIABETES }
No 2

ALL original official copies MUST be kept and ONLY edited by Data Management

Q. 153. Are you physically active for more than 30 minutes every day? This includes physical activity during work, leisure, or regular daily routine.

Yes 1 |__| {PHYACTIVE}
No 2

Q.154. How often do you eat fruit or vegetables?

Every day 1 |__| {DAYSVEGFRUITS}
Not every day 2
Does not take 3

Q. 155. How many servings of vegetables/fruits do you eat on one of those days?

|__|__| {VEGFRUITSSERVE}

COMPUTER 1D	CURRENT ID

TIME ENDED |__|__|:|__|__| am/pm{TEND}

Team Leader # |___|___|___|{TEAMLEAD) Editor |____|____|____|{EDITOR}QC# |____|____|___|{QC}

Data entry clerk #|__|__|__|__|{DATA CLERK} {DE DATE dd mm yy} ___/___/___/

ALL original official copies MUST be kept and ONLY edited by Data Management

PARTNER IDENTIFICATION

**PLACE COMPUTER ID HERE**

Int_date ___/___/20___
dd mm yyyy

VISIT # |R|19| {VISITNO}

Current ID

SuperCluster # |__|__|__|{REGION}

Community # |__|__|__|{COMM_NUM

HH # |__|__|__|__|{HH_NUM}

Member # |__|__|__|{MEMBER_NUM

FOR ALL RESPONDENTS IN MARRIAGE OR CONSENSUAL UNION TRY TO OBTAIN INFORMATION THAT WILL ALLOW US TO LINK THE INDIVIDUAL TO HER MARRIAGE OR CONSENSUAL PARTNERS WITHIN THE SAME HOUSEHOLD OR WITHIN THE SAME COMMUNITY. PAY ATTENTION TO CASES OF POLYGAMY WHERE WIVES MAY BE LIVING WITHIN ONE HOUSE OR IN DIFFERENT HOUSEHOLDS. USE ADDITIONAL PAGES IF >3 PARTNERS.

FOR SAME HOUSEHOLD PARTNERS, TRY TO FIND THE MEMBER # FROM THE HOUSEHOLD CENSUS LISTING. FOR PARTNERS IN DIFFERENT HOUSEHOLDS, THE INFORMATION WILL HAVE TO BE COMPLETED LATER.

"Previously, you told me that you are married/you have a wife even though you might not be officially married".

Partner 1 **BLOCK #** |__|{**BLOCKNO1**}

Do you have a wife with whom you live in this house?

If yes, What are her names? **(Please let me know all the names by which she is commonly known)**

____

Current ID # for partner 1: |____|____|____|/|____|____|____|/|____|____|____|___|/|____|____|____|
(Supercluster /community /household /member)

If no, Does she commonly reside in this community?
Yes ...1 No...2 Out spouse.....3

What is her name?

____

EDITOR: TRY TO LOCATE ID FROM HOUSEHOLD CENSUSES IN COMMUNITY.

Current ID # for partner 1: |____|____|___|/|____|____|____|/|____|____|____|___|/|____|____|___|
(supercluster/ community/ household/ member)

(If not) Can you please tell me where she stays?

If polygamous ask,

You have reported having ______wives (mention number in Q.11). Thinking about the order in which you married these______ (number) wives, what number is this wife among the wives you currently have?
|__|__| {MARTORP1}

ALL original official copies MUST be kept and ONLY edited by Data Management

Partner 2 BLOCK # |__|{BLOCKNO2}
Do you have a wife with whom you live in this house?

If yes, What are her names? **(Please let me know all the names by which she is commonly known)**

____

Current ID # for partner 1: |____|____|____|/|____|____|____|/|____|____|____|___|/|____|____|____|
(Supercluster /community /household /member)

If no, Does she commonly reside in this community?
Yes ...1 No...2 Out spouse 3
What is her name?

____

EDITOR: TRY TO LOCATE ID FROM HOUSEHOLD CENSUSES IN COMMUNITY.
Current ID # for partner 1: |____|____|___|/|____|____|____|/|____|____|____|___|/|____|____|___|
(supercluster/ community/ household/ member)
(If not) Can you please tell me where she stays?

If polygamous ask,
You have reported having ______wives (mention number in Q.11). Thinking about the order in which you married these______ (number) wives, what number is this wife among the wives you currently have?
|__|__| {MARTORP2}

Partner 3 BLOCK # |__|{BLOCKNO3}
Do you have a wife with whom you live in this house?

If yes, What are her names? **(Please let me know all the names by which she is commonly known)**

____

Current ID # for partner 1: |____|____|____|/|____|____|____|/|____|____|____|___|/|____|____|____|
(Supercluster /community /household /member)

If no, Does she commonly reside in this community?
Yes ...1 No...2 Out spouse 3
What is her name?

____

EDITOR: TRY TO LOCATE ID FROM HOUSEHOLD CENSUSES IN COMMUNITY.
Current ID # for partner 1: |____|____|___|/|____|____|____|/|____|____|____|___|/|____|____|___|
(supercluster/ community/ household/ member)
(If not) Can you please tell me where she stays?
If polygamous ask,
You have reported having ______wives (mention number in Q.11). Thinking about the order in which you married these______ (number) wives, what number is this wife among the wives you currently have?
|__|__| {MARTORP3}

Partner 4 **BLOCK # |__|__|{BLOCKNO4}**

Do you have a wife with whom you live in this house?

If yes, What are her names? **(Please let me know all the names by which she is commonly known)**

____

Current ID # for partner 1: |____|____|____|/|____|____|____|/|____|____|____|___|/|____|____|____|
(Supercluster /community /household /member)

If no, Does she commonly reside in this community?
Yes ...1 No...2 Out spouse 3
What is her name?

____

EDITOR: TRY TO LOCATE ID FROM HOUSEHOLD CENSUSES IN COMMUNITY.
Current ID # for partner 1: |____|____|___|/|____|____|____|/|____|____|____|___|/|____|____|___|
(supercluster/ community/ household/ member)
(If not) Can you please tell me where she stays?

If polygamous ask,
You have reported having ______wives (mention number in Q.11). Thinking about the order in which you married these______ (number) wives, what number is this wife among the wives you currently have?
|__|__| {MARTORP4}

ALL original official copies MUST be kept and ONLY edited by Data Management
